# Development of Low Temperature Activatable Aryl Azide Adhesion Promoters as Versatile Surface Modifiers

**DOI:** 10.1021/acsaenm.5c00002

**Published:** 2025-04-04

**Authors:** Alexandros A. Atzemoglou, Niccolò Bartalucci, Felix Donat, Mark W. Tibbitt, Samuele G. P. Tosatti, Stefan Zürcher

**Affiliations:** † SuSoS AG, Lagerstrasse 14, Dübendorf 8600, Switzerland; ‡ Department of Mechanical and Process Engineering, Macromolecular Engineering Laboratory, ETH Zurich, Zurich 8092, Switzerland; § Department of Mechanical and Process Engineering, Laboratory of Energy Science and Engineering, ETH Zurich, Zurich 8092, Switzerland

**Keywords:** azide, nitrene, adhesion promoters, low temperature activation, coating, DFT, C–H insertion, surface functionalization

## Abstract

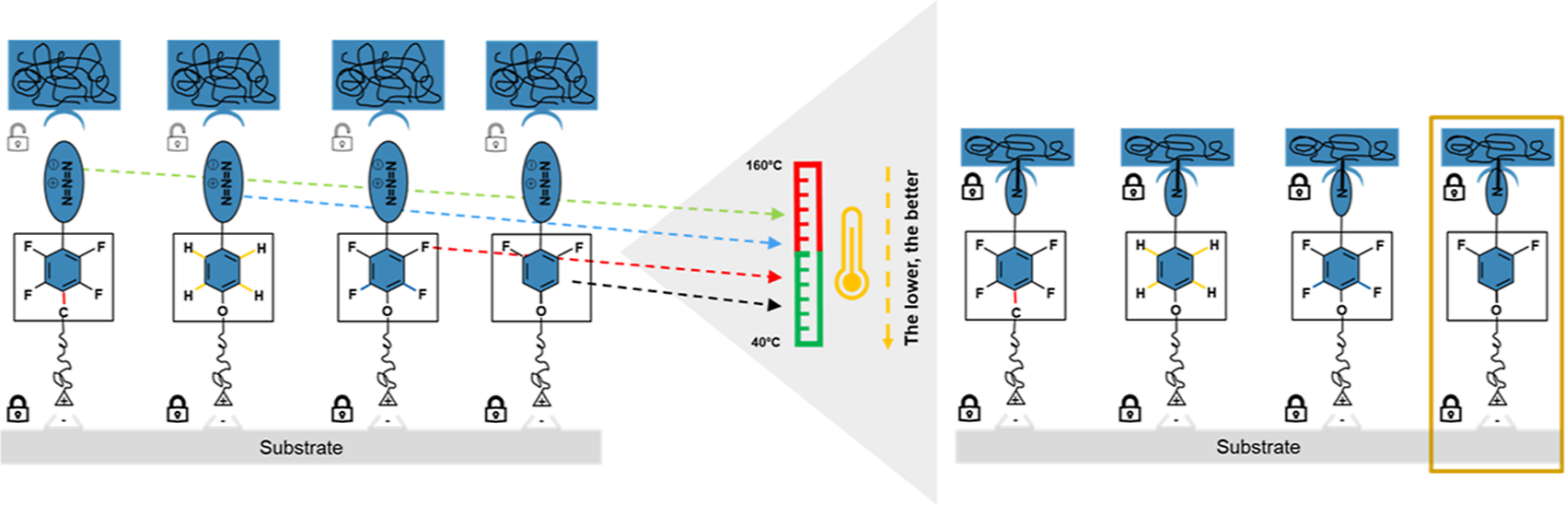

An innovative approach for the immobilization of polymeric films is the use of bifunctional compounds called adhesion promoters, that create a stable chemical “bridge” between materials, allowing for versatile and permanent surface modification. To connect coating materials that lack reactive groups, the “bridge” forming adhesion promoter needs a highly reactive group that can insert in otherwise unreactive chemical bonds. Activatable perfluoro-aryl azides are commonly used to achieve this, with the limitation that their thermal activation is constrained to elevated temperatures—typically far above 100 °C—and photolytic activation is often unfeasible due to geometry or opaque materials. To overcome this limitation, we designed and synthesized three small organic molecules based on substituted aryl azides with the aim of lowering the activation temperature that restricts the use of existing aryl azides. We demonstrate both computationally via density functional theory (DFT) calculations and experimentally that the activation temperature of an aryl azide can be tuned by varying its substituents, giving access to mild activation temperatures (below 100 °C). The most reactive compound was the ο,ο-difluoro substituted *p*-phenoxy azide, which forms a nitrene and undergoes C–H insertion reactions at temperature of around 70 °C. This allows functionalization of surfaces with polymers that have no reactive groups under gentle conditions. The synthesized molecules were incorporated into a polymeric backbone to form adhesion promoters allowing covalent attachment of polymeric films to substrates by thermal activation below 100 °C. As an example, we successfully generated monomolecular films of polyvinylpyrrolidone (PVP), a polymer used and approved for medical devices due to its hydrophilic and lubricious properties. The effectiveness of attachment was assessed qualitatively and quantitatively using spectroscopic ellipsometry (ELM) and X-ray photoelectron spectroscopy (XPS).

## Introduction

**Figure 1:**
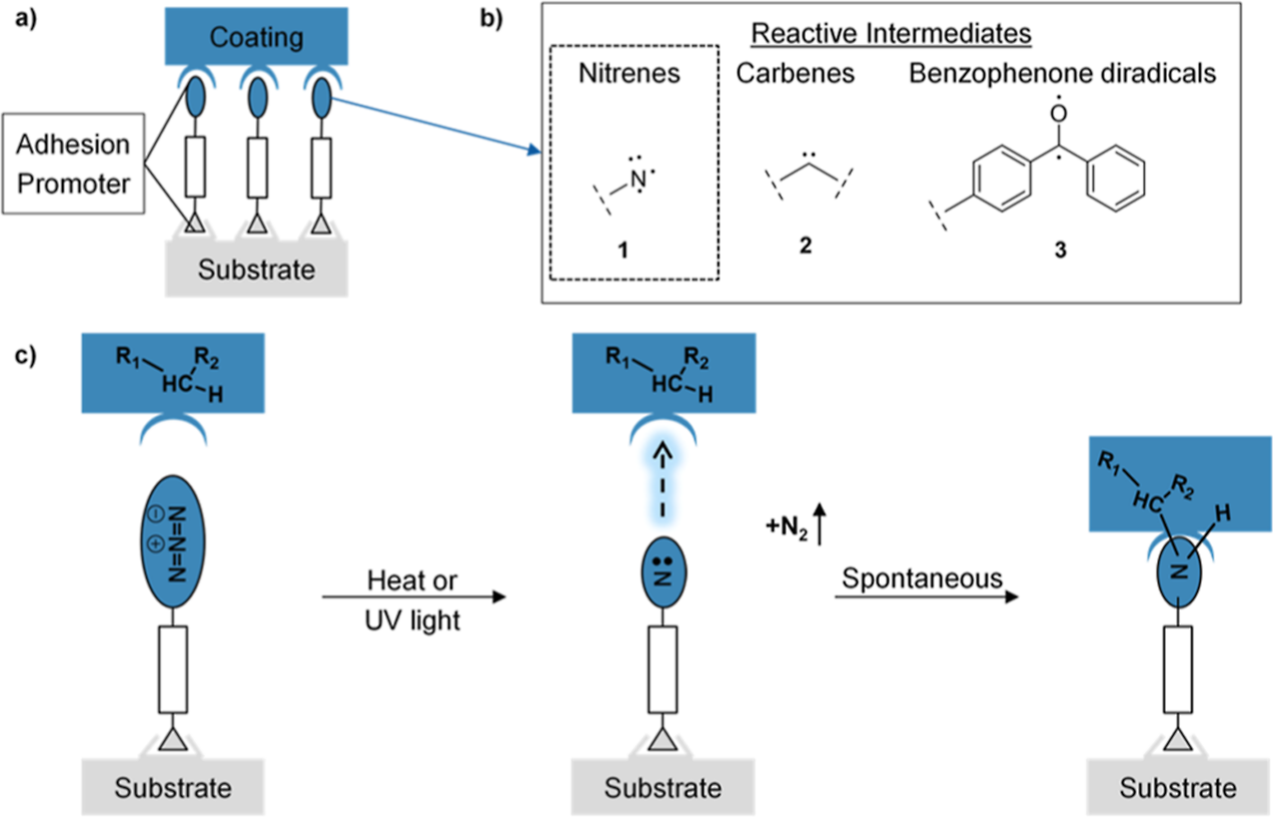
(a) Attachment of a coating material on a chemically non-compatible substrate is possible by using a bifunctional adhesion promoter which acts as a chemical “bridge”, (b) three main reactive intermediates used as universal adhesion promoters: Nitrenes (**1**), carbenes (**2**) and benzophenone diradicals (**3**), (c) adhesion promoter containing aryl azide, decomposition to nitrene formation and C–H insertion reaction.

In the field of surface engineering, reactive bifunctional molecules, such as adhesion promoters, are used for permanent surface modification with polymeric coatings that would not bind otherwise by creating a chemical “bridge” to the substrate (Figure 1). These processes are valuable as they can introduce a desired surface functionality without compromising the properties of the underlying substrate material.^1^ However, they are not always straightforward since state of the art adhesion promoters are only effective for a limited number of classes of substrates and coatings. Further, the activation of surface modifying molecules are often induced either photolytically in the UV range or thermally.^2^ Photolytic activation can be a limiting factor, as not all materials are transparent or stable to the activating wavelengths, often in the deep UV-range (<300 nm). In these cases, adhesion promoters that can be activated thermally at low temperature (<100 °C) would be highly advantageous to prevent degradation and deformation of the substrate and/or degradation of the top-coating, of any involved polymer.

Currently, carbenes, nitrenes and benzophenone diradicals are commonly used reactive intermediates for insertion reactions (Figure 1b).^3^ Nitrenes are the most prominent as their precursors are more stable than the respective carbenes for efficient bimolecular chemistry,^4^ while the chemical modification of benzophenone derivatives^5^ to influence their activation barrier is more difficult.^6,7^ Thus, we selected nitrene reactive groups, which are the product of the decomposition of the respective azide, for this work.^8^

Phenylazide and its derivatives are widely used as reactive compounds, as they can universally react via nitrogen elimination of the azide into highly reactive singlet nitrene species that can undergo a plethora of intra- or intermolecular reactions, including insertion reactions into C–H/N–H bonds to form new covalent linkages (Figure S1).^9,10^ However, this reaction is classically induced either by UV-light or thermally (typically well above 100 °C), which constrains their use for the permanent modification of low glass transition temperature polymeric materials.^11^ Thus, there is a need to generate azides for efficient crosslinking at low temperature (below 100 °C) and for cases where photolytic activation is unfeasible.

The activation temperature of the unsubstituted phenylazide is above 140 °C.^12^ Further, the decomposition of the perfluorinated phenylazide starts at temperatures above 130 °C, as used for the immobilization of ultrathin polymer films.^13^ There are only a few phenylazides with known thermal decomposition below 100 °C, and they decompose in a concerted intramolecular mechanism.^14,15^ Such concerted intramolecular mechanisms are also observed in other ortho-substituted phenylazides unless the substituent is a halogen (Figure S2).^16^ For unsubstituted phenylazides, the main decomposition path is also intramolecular via azirine intermediate to azepine.^17^ As a result, these azides have very low to undetectable yields of intermolecular bonds formed via insertion reactions to nearby molecules (Figure S1).

Experimental and theoretical studies have extensively investigated the stability and reactivity of nitrenes, especially of the singlet nitrenes that are initially formed upon decomposition. Di-ortho-substitution of the phenylazide with a halogen (fluorine, chlorine, or bromine) increases the lifetime of singlet nitrene to 260 ns at 25 °C. The corresponding unsubstituted compound has a lifetime of less than 1 ns at 25 °C. A longer lifetime of the produced nitrene increases the likelihood of intermolecular insertion reactions over intramolecular rearrangements.^18−22^ Even though the effect of the ortho substituents towards nitrene formation and stability has been well studied in the literature, only a few reports have investigated the influence of different substitution patterns on the activation energy of an aryl azide and the stability of the formed nitrene intermediate.^23^ We hypothesized that strategic chemical functionalization of parent aryl azides may provide a handle to synthesize low temperature activatable adhesion promoters.

We assumed that the presence of a π-donating substituent in para position to the aryl azide will influence the activation barrier for nitrene formation and stabilize the generated nitrene. We also wanted to investigate if the combination of π-donation and electronegativity/electron withdrawing effects lead to stabilization of the benzene ring and destabilization of the azide group, leading to a decreased activation barrier to nitrene formation. It can be safely assumed that a lower energy barrier for the N^1^–N^2^ bond cleavage in Aryl-N^1^–N^2^N^3^, will correlate with a lower temperature necessary to thermally activate the azide, and generate the highly reactive nitrene intermediate.

Here, we report the development of reactive adhesion promoters capable of creating covalent bonds with polymeric materials at temperatures below 100 °C. Three differently substituted aryl azides were synthesized, and their thermal decomposition properties were investigated using thermogravimetric analysis (TGA/DSC), mass spectroscopy (MS) and infrared spectroscopy (IR). The azide bond-cleavage energy was calculated using density functional theory (DFT) and compared to experimental results. The differently substituted aryl azide molecules were grafted to poly(allyl amine) and the produced adhesion promoters were surface anchored onto silicon wafers by means of electrostatic self-assembly via the positive charges from protonated polyallylamine and the negatively charged SiO_2_ surface.^24^ Polyvinylpyrrolidone (PVP) was spin-coated onto the functionalized substrates and the specimens were activated by heating over a range of temperatures to assess the effectiveness of the nitrenes to covalently bond with such a polymer that has no reactive groups. The most reactive of the three compounds, ο,ο-difluoro substituted *p*-phenoxy azide, could be activated at temperatures as low as 70 °C and generated strongly bound PVP films that could not be removed by extensive rinsing. The aryl azides developed in the research reported here serve as a platform to generate adhesion promoters capable of universally bonding otherwise incompatible materials by mild temperature activation.

## Results and Discussion

### Azide to Nitrene Bond-Cleavage Energy as a Function of Aryl Substitution

Based on a comprehensive literature review and supported by chemical intuition, the hypothesis that the thermal bond-cleavage of an azide can be tuned by introducing a π-donating group in para position, as well as supporting groups in ortho positions to the azide, was tested on a series of compounds constituted of a benzene ring bearing an azide group. The advantages of this architecture are among others, the broad access to molecules containing a benzene ring and the plethora of extensively studied reactions. Further, due to the delocalized electrons of the aromatic benzene ring, substitution leads to long range electronic effects that can influence the properties of other substituents, such as the azide in our case, which is not possible for non-conjugated systems.^25,26^

**Figure 2:**
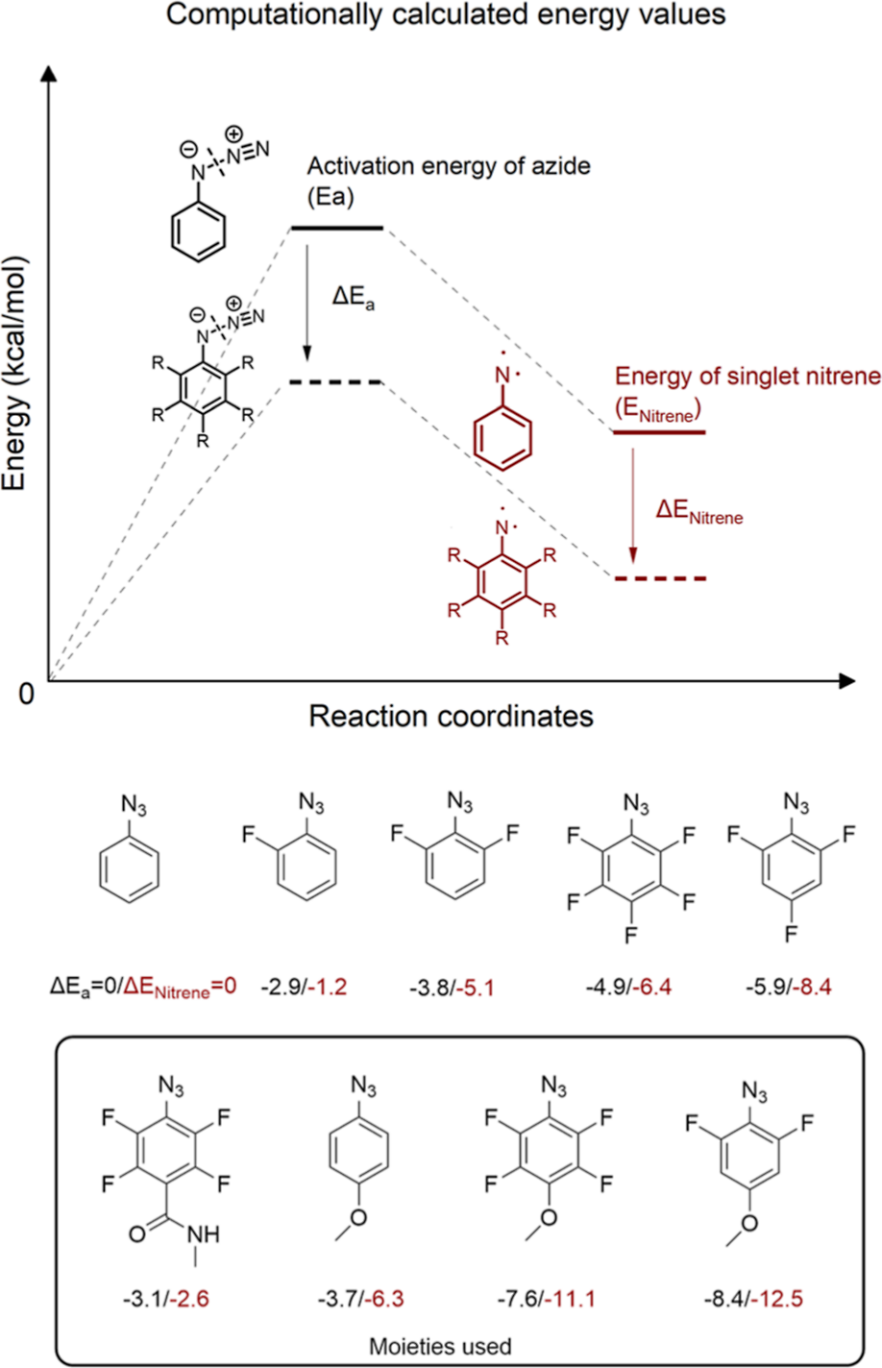
The bond-cleavage energy (activation energy of azide *E*_*a*_) of the bond breakage of the ArylN^1^–N^2^N^3^ bond to form the corresponding singlet nitrene RN^•^ + N_2_↑ was calculated alongside with the energy of the formed singlet nitrene for differently substituted phenylazides. The difference in energy values relative to the reference phenylazide are shown below each structure, on the left side of the slash symbol it represents the ΔE_a_ of the azide while on the right side the energy difference of the formed nitrene Δ*E*_Nitrene_ is reported. Negative values mean decrease and positive values mean increase, relative to the reference compound. The lower the calculated bond-cleavage energy is, the lower will be the effective starting decomposition temperature.

Prior to synthesizing new species, we used density functional theory (DFT) to quantify the effect of different potential substitutions of aryl azides on the bond-cleavage energy towards nitrene formation, as well as on the energy of formation of the singlet nitrene and, thus, its stability.^27,28^ The surface energy potential was calculated for different compounds as a function of the distance between the first and second nitrogen atom in the aryl azide group (ArylN^1^–N^2^N^3^). The maximum occurring along this reaction coordinate defines the bond-cleavage energy *E*_a_ (Figure S3). Separating the generated N_2_ molecule more than 3 Å from the remaining nitrene leads to a plateau in the surface potential curve defining the energy of the generated nitrene (plus N_2_). The unsubstituted phenylazide with calculated *E*_a_ = 40.9 kcal/mol was used as our reference and we then determined Δ*E*_a_ (*E*_a_ of phenylazide reference minus *E*_a_ of substituted aryl azide compound) and Δ*E*_Nitrene_ (nitrene energy of phenylazide reference minus nitrene energy of substituted aryl azide compound) for a series of substituted compounds (Figures 2 and S4a).

DFT calculations corroborated the hypothesis that the presence of π-donating F-substituents in the ortho and para positions lowered the bond-cleavage energy for nitrene formation. The π-electrons of the substituents can donate their electrons and contribute via resonance. The di-ortho fluorine substitution pattern was found to effectively reduce the bond-cleavage energy (*E*_a_) of the azide (−3.8 kcal/mol) and the energy of the formed nitrene (−5.1 kcal/mol) compared with the reference phenyl azide (Figure 2). Moreover, the presence of a π-donating group such as fluorine or oxygen in the para position, also decreased the bond-cleavage energy of the azide decomposition (−1.4 kcal/mol for F and −3.7 kcal/mol for a methoxy substituent) by further stabilizing the benzene ring, making the breakage of the ArylN^1^–N^2^N^3^ bond even easier. One oxygen in para position leads to a slightly lower azide bond-cleavage energy (−3.7 kcal/mol) compared with fluorine atoms in the ortho positions and an amide group in para position, as in perfluorophenylazide (−3.1 kcal/mol). Even though the presence of a methoxy group in ortho position to the azide (−4.8 kcal/mol) would be even better than fluorine (−2.9 kcal/mol) for lowering the bond-cleavage energy (Figure S4b), it would likely lead to an intramolecular decomposition path that consumes the generated nitrene through C–H insertion to form a 5-membered dihydro–oxazole ring before enabling intermolecular coupling reactions.^29,30^ In contrast, a fluorine substitution in the meta position has a counterproductive effect on the activation energy (+0.2 kcal/mol, Figure S4a), and thus it is suggested that π-substituents in meta positions should be avoided. The combination of two F atoms in ortho position and a methoxy group in the para position gave the greatest energy reduction for both the bond-cleavage energy and the generated nitrene (Δ*E*_a_ of −8.4 kcal/mol and Δ*E*_Nitrene_ of −12.5 kcal/mol; Figure 2).

### Synthesis of Bifunctional Linker

To generate aryl azide functionalized adhesion promoters, typically bifunctional linkers such as the commercially available succinimidyl-4-azido-2,3,5,6-tetrafluorobenzoate (PFPA-NHS) are widely used as starting materials.^24,31,32^ To experimentally test the outcome of the computational calculations, we synthesized three NHS functionalized aryl azides (4-azido-3,5-difluorophenoxy) butanoic acid NHS ester (**5a**), (4-azido-2,3,5,6-tetrafluorophenoxy) butanoic acid NHS ester (**5b**), and (4-azido-phenoxy) butanoic acid NHS ester (**5c**) differing in their substitution pattern based on the calculated structures in Figure 2 but having an additional butanoic acid linker with an NHS ester for further possibility to link it to any amine functionalized adhesion promoter. PFPA-NHS was used as the commercially available reference lacking the π-donating phenyl ether in para position. With this design, the aryl azide moieties can be easily functionalized with different surface-active groups containing amines to generate adhesion promoters tailored to attach to different substrates.

**Scheme 1:**
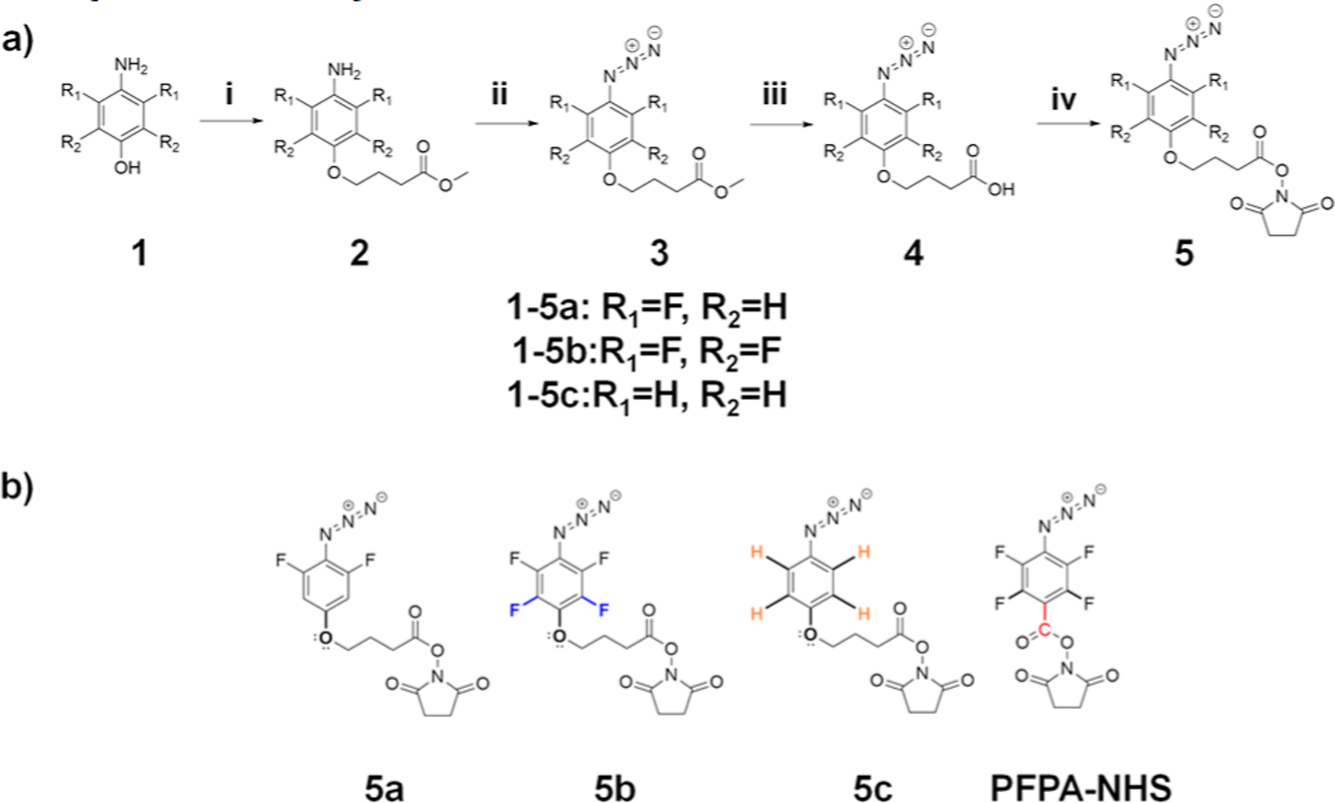
Synthesis of the Three Aryl Azides, (4-Azido-3,5-difluorophenoxy) Butanoic Acid NHS Ester (**5a**), (4-Azido-2,3,5,6-tetrafluorophenoxy) Butanoic Acid NHS Ester (**5b**) and (4-Azido-phenoxy) Butanoic Acid NHS Ester (**5c**) and Comparison of the Three Structures with Succinimidyl-4-Azido-2,3,5,6-Tetrafluorobenzoate (PFPA-NHS) Reagents and conditions: (i) DMF, methyl-4-bromobutyrate, K_2_CO_3_, 85 °C for 2a/100 °C for 2b/60 °C for 2c, 4 h, (ii) TFA, NaNO_2_, NaN_3_, 0 °C to RT, 1 h. Column chromatography silica gel, hexane:Et_2_O (3:1), (iii) MeOH, NaOH, RT, overnight, HCl, (iv) DCM, DCC, RT, overnight. (b) Molecular structures of DFPxA-NHS (**5a**), PFPxA-NHS (**5b**), PxA-NHS (**5c**) and PFPA-NHS with the differences highlighted by color.

To obtain the three compounds of interest (**5a**, **5b**, and **5c**), a retrosynthetic analysis was performed to design the synthetic protocols and identify starting materials and reagents. Differently fluorine substituted 4-amino-phenols were chosen as starting materials (**Synthetic procedures**) based on the capability of the amino group (–NH_2_) to be converted to the desired azide group, and the hydroxyl group (–OH) to be modified accordingly for the formation of a second functional group. Because of the expected low activation energies of the azides, the modification of the hydroxyl group, which requires elevated temperatures (>60 °C), was performed at first. Further, all the reaction steps involving the azide were performed at the lowest temperature possible to avoid any potential decomposition, assuming the activation barrier was successfully decreased. All three compounds (**5a**, **5b**, and **5c**) were successfully synthesized following a four-step synthesis starting with an alkylation of the phenol using methyl-4-bromobutyrate, followed by the conversion of the aniline into the azide, basic ester hydrolysis, and conversion of the generated carboxylic acid into the reactive *N*-hydroxy succinimide (NHS) ester (Scheme 1). In Scheme 1b, the molecular structures of the 4 aryl azides are depicted highlighting the differences between DFPxA-NHS (**5a**), which consists of two fluorine atoms in the ortho positions and more importantly a π-donating oxygen atom in para position to the azide; PFPxA-NHS (**5b**), which has two additional fluorine atoms in meta position to the azide; PxA-NHS (**5c**), which has only hydrogen atoms in ortho and meta positions to the azide; and succinimidyl-4-azido-2,3,5,6-tetrafluorobenzoate (PFPA-NHS), which besides the two additional fluorine atoms in meta position has a carboxylic group in para position to the azide.

### Decomposition Temperature and Decomposition Kinetics

**Figure 3:**
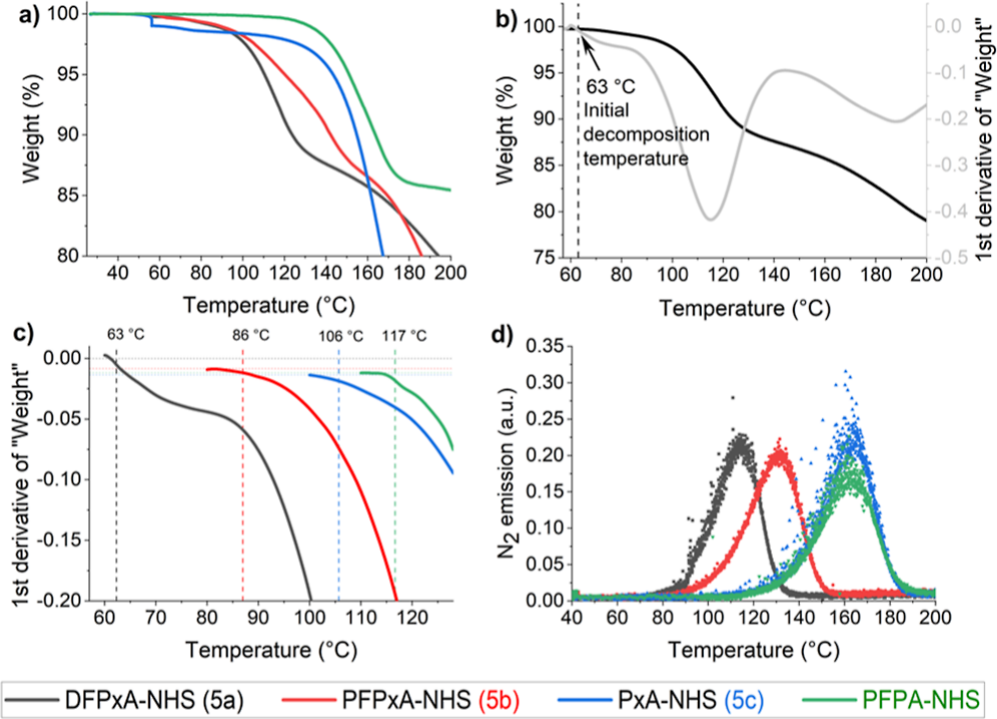
(a) Comparative TGA figure of the normalized sample weight as function of temperature for all four compounds (**5a**, **5b**, **5c**, PFPA-NHS) shows the expected trend from low to high activation energy, (b) TGA data of weight loss and the first derivative of weight loss (DTG) plotted as function of temperature of DFPxA-NHS (**5a**) in a *xyy* TGA-DTG figure. By plotting the first derivative of weight, it is possible to identify the initial decomposition point of a compound since even minor changes can be identified.^34^ The stated value represents the initial decomposition temperature of DFPxA-NHS, (**5a**). (c) Comparative DTG figure of the first derivative of all four compounds over increasing temperature. The initial azide decomposition temperature is determined as the point where a deflection is first observed from its respective baseline.^33^ A different baseline was used for each compound as different amounts of entrapped solvents were present, (d) N_2_ decomposition product measured by mass spectrometry over increasing temperature, verifying the decomposition trend determined by TGA.

Since the decomposition of azides is an exothermic reaction with release of dinitrogen (Figure 1c), Thermogravimetric Analysis (TGA) measurements were performed to observe the changes in the weight of the sample with increasing temperature (Figure 3). Upon thermal degradation of the compounds, the first group to decompose is the azide (–N_3_) as the most heat sensitive group in each of the molecules tested and, thus, it was feasible to identify the onset temperature, considered as the initial decomposition temperature, for each of the three synthesized molecules (**5a**, **5b**, and **5c**) and PFPA-NHS (Figure 3a). The onset temperature was determined according to ASTM E2550,^34^ described as the temperature where the first deflection from the established baseline prior to the thermal event can be identified. This deflection is easiest to identify in the first derivative of the weight vs. temperature curve (Figure 3b,c).

The decomposition of DFPxA-NHS (**5a,** black curve) initiated at the lowest temperature with an initial decomposition point at around 63 °C (Figure 3a,c). This corroborated our hypothesis that the combination of the two electronegative fluorine atoms in ortho position to the azide as well as the π-donating oxygen atom in para position lowered the activation barrier of the azide towards nitrene formation. The initial decomposition point of the azide of PFPxA-NHS (**5b,** red curve) is higher (around 86 °C), indicating that the presence of the two extra fluorine atoms in meta positions to the azide increase its initial decomposition temperature compared to **5a**. PxA-NHS (**5c**) started to decompose at even higher temperature (around 106 °C), showing that the absence of any fluorine atom as aromatic substituents further increases the activation barrier and thus the initial decomposition temperature. PFPA-NHS was the compound with the highest initial decomposition point (around 117 °C) of the four azides studied, indicating that the presence of a carbon atom in para position that lacks a π-donating lone pair, is the critical factor that leads to the increased decomposition barrier. Further, the experimental results aligned well with the DFT calculations and supported the hypotheses on the impact of select chemical modifications to the activation temperature of the aryl azides.

To further validate our findings, mass spectrometry (MS) measurements were performed over a range of increasing temperatures to observe the emission of dinitrogen (N_2_), which is the decomposition product of the reactive azides. The results of the MS experiments confirmed that the experimentally observed mass loss in the TGA analysis was indeed due to the loss of N_2_ (Figure 3d). The same trend for the decomposition temperature, as found during the TGA analysis and from the DFT calculations, was observed for the four studied compounds with **5a** decomposing at the lowest temperature, followed by **5b** < **5c** and PFPA-NHS.

**Figure 4:**
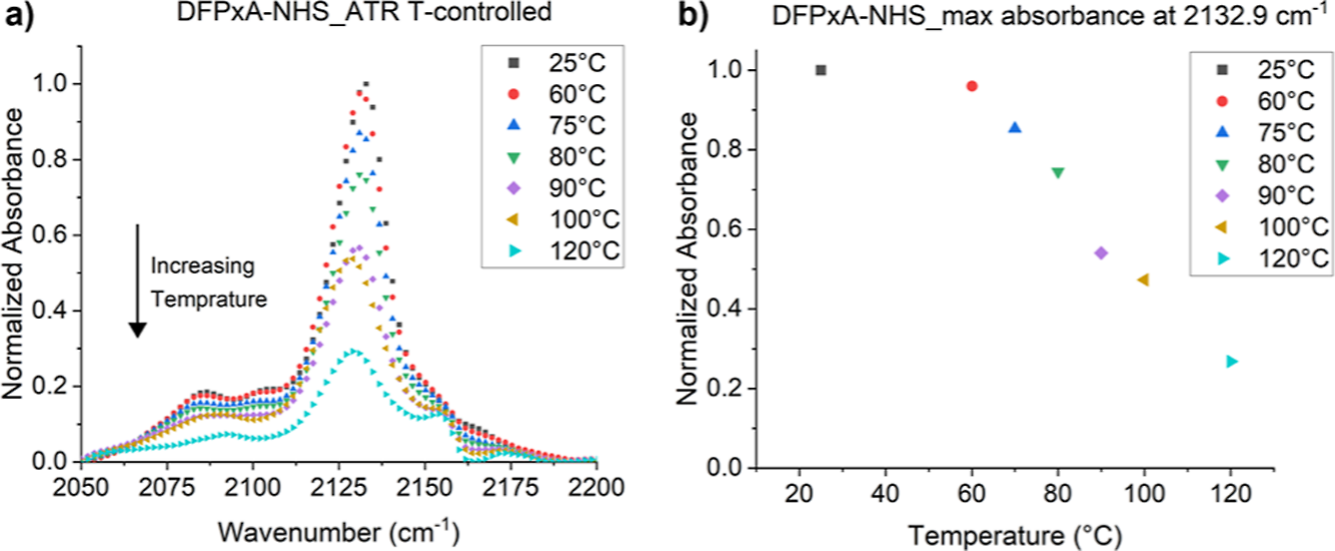
(a) In Situ ATR-IR spectra of the azide stretching of DFPxA-NHS (**5a**) plotted after heating at different temperature for 15 min. (b) Peak intensity normalized to the room temperature absorbance value as function of heating at increased temperature.

Azides display a characteristic asymmetric stretching vibration in the IR-spectrum at ∼2130 cm^–1^. The quantification of this peak intensity as a function of temperature can be used to follow the decomposition of the azide group. The decomposition of DFPxA-NHS (**5a**) was measured using heat-controlled ATR-infrared spectroscopy. The intensity of the azide peak began to decrease above 60 °C again demonstrating the low decomposition temperature of this compound (Figure 4).

**Figure 5:**
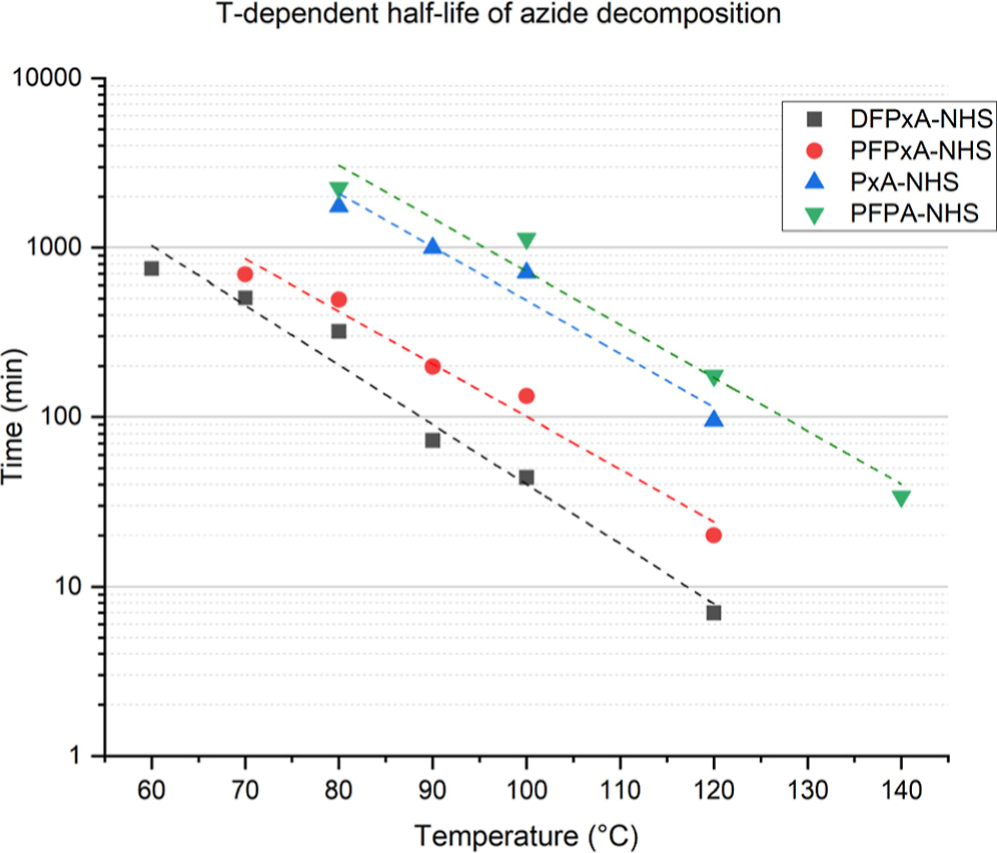
Measured and curve fitted (dotted lines) half-life of the azide decomposition determined form isothermal TGA measurements. The higher the temperature, the faster the reaction proceeds in all cases as expected for a unimolecular decay (note the logarithmic time scale).

Having demonstrated that chemical substitutions can lower the onset temperature of decomposition in aryl azides, we next quantified the decomposition kinetics of the four compounds via TGA. By isothermal heating of the compounds at different temperatures and monitoring the weight change, the decay of the azide concentration can be plotted logarithmically versus time at each investigated temperature verifying the first order reaction kinetics of the azide decomposition (Figure S5). The reaction rate *k* at each temperature was calculated from the slopes of the respective curves (Table S1). The exact procedure is described in the Materials and Methods section. This analysis provided the half-life of the four compounds as a function of temperature (Figure 5). The half-life provides an understanding of how long a chemical process needs to react the azides into nitrenes for cross-coupling reactions at a given temperature. At 100 °C, DFPxA-NHS (**5a**) had a half-life of 44 min, PFPxA-NHS (**5b**) of 133 min, PxA-NHS (**5c**) of 711 min, and PFPA-NHS of 1130 min. The absolute “large” difference (711 min for 5c vs. 1130 min for PFPA-NHS) is due to the exponential nature of the kinetic dependance (note the logarithmic scale in Figure 5). It can be expected that the deviation to the fitted curve gets larger the slower the reaction is and therefore also the uncertainty of the measured slope in Figure S5, respectively the calculated *k*(*T*) in Table S1.Further, all compounds exhibited an exponential increase (linear in the logarithmic presentation) in the reaction rate as function of temperature as it is expected for a unimolecular reaction following first order kinetics (Figure 5). The same order of stability with DPFPxA-NHS (**5a**) as the fastest decomposing compound from the series was again observed.

**Figure 6:**
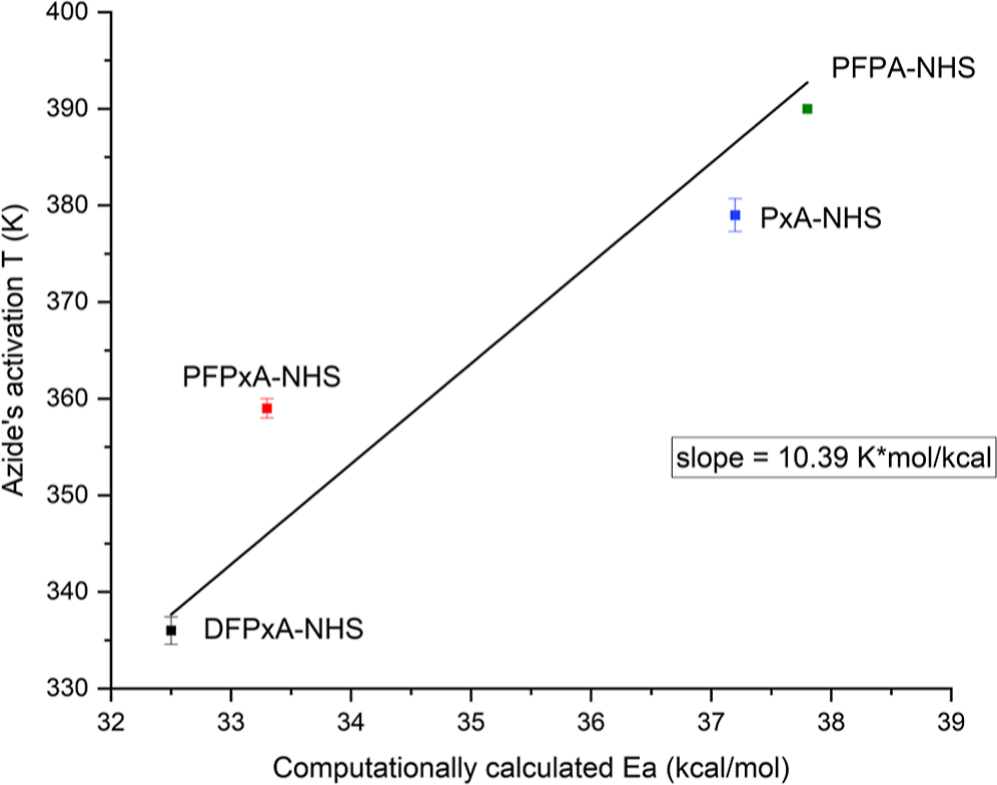
Correlation of the experimentally measured activation temperature versus the computationally calculated azide bond-cleavage energies *E*_a_ from DFT. The data are in line and the pattern is as expected, meaning increasing bond-cleavage energy as computed corresponds to increasing activation barrier. The straight line corresponds to a linear regression through the origin.

Finally, we compared the computed bond-cleavage energies from DFT calculations with the experimentally determined azide activation temperatures. The experimental and computational data aligned well for all compounds (Figure 6), demonstrating that DFT calculations are a powerful predictor and indispensable tool for chemical synthesis, especially for small organic molecules and for common parameters. This comparison validated that increased azide bond-cleavage energy corresponded to increased activation temperature, as expected given that the higher the azide bond-cleavage energy, the more thermal energy needs to be provided to the system for an azide to decompose.

It is generally considered that no chemical reactions occur at absolute zero (0 K). By fitting the data in a linear regression intercepting at 0 K, the resulting fitting line sufficiently describes the data points. The slope of the fitted linear regression suggests that for the specific data set consisting of differently substituted aryl azides, an increase of 1 kcal/mol in the value of bond-cleavage energy resulted in a 10 K increase in decomposition temperature.

### Adhesion Promoter Synthesis

**Figure 7:**
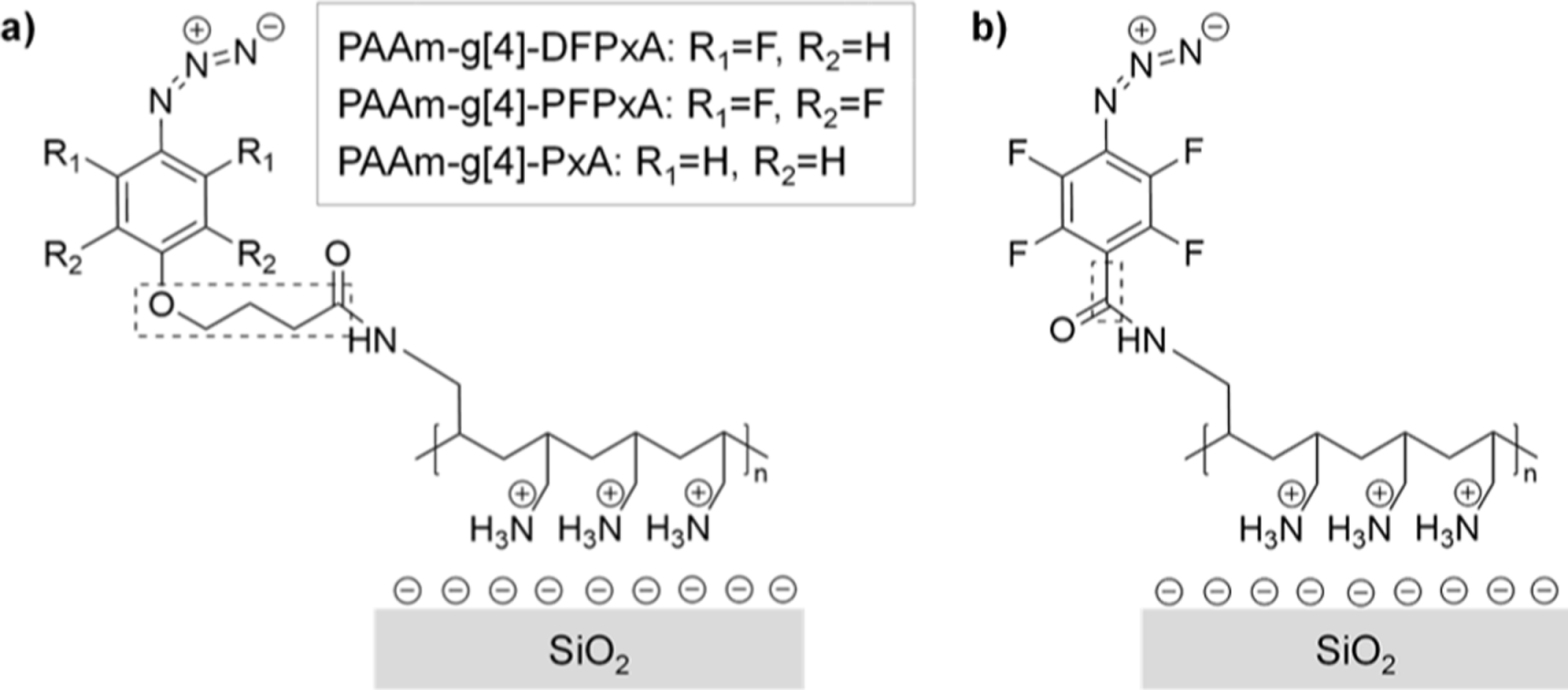
(a) Polymeric structure of PAAm-*g*[4]-DFPxA/PFPxA/PxA and (b) of PAAm-*g*[4]-PFPA adhesion promoters, electrostatically adsorbed onto negatively charged SiO_2_ substrate.^31^ Note that the indicated spacers connecting the aromatic ring to the amide group are different.

To test the synthesized reactive aryl azides for low temperature surface functionalization, a series of four adhesion promoters were synthesized. As proof of principle and to directly compare with previous results using PFPA-NHS,^24,31^ polyallylamine hydrochloride (PAAm HCl) was used as a stable model polymeric backbone to which each of the four aryl azide moieties were grafted. Using a simple dip and rinse process from neutral aqueous buffer solution, these graft copolymers (PAAm-*g*-arylazide) were then electrostatically self-assembled onto SiO_2_ substrates. The excess amine groups of the polyallylamine backbone polymer become protonated in neutral buffer and therefore positively charged, while SiO_2_ at pH = 7 is negatively charged, resulting in strong electrostatic attraction between the adhesion promoter and the substrate (Figure 7).

Coating of homogeneous monomolecular adhesion promoter films is critical for the accurate quantitative characterization of the activation of the aryl azides, which can be verified by Ellipsometry (ELM) and X-ray Photoelectron Spectroscopy (XPS).^35^ The thicknesses of the adsorbed films for the three newly synthesized adhesion promoters (PAAm-*g*-DFPxA, PAAm-*g*-PFPxA and PAAm-*g*-PxA) were similar and all slightly higher (1.8–2 nm) than the layer thickness of PAAm-g-PFPA (1.4 nm) as they each contained the same additional propyloxy spacer in the para position while PFPA is linked directly via its carboxylic group to the polyallylamine backbone (Table 1).

**Table 1:** Surface Characterization of the Coated Adhesion Promoter Layers by Ellipsometry (ELM) Measurements and X-ray Photo-Electron Spectroscopy (XPS) Analysis

Adhesion Promoter	ELM		XPS (normalized At.-%)				
	d [nm]		Si 2p	C 1s	F 1s	N 1s	O 1s
PAAm-*g*[4]-DFPxA	2.03 ± 0.21	apparent At %^a^	24.1	28.0	1.4	5.2	41.3
		overlayer At %^b^		72.3	3.7	13.5	10.5
		theor. At %^c^		65.6	6.2	21.9	6.3
PAAm-*g*[4]-PFPxA	1.84 ± 0.23	apparent At %^a^	25.2	26.8	2.4	5.6	39.9
		overlayer At %^b^		74.4	6.7	15.6	3.3
		theor. At %^c^		61.8	11.7	20.6	5.9
PAAm-*g*[4]-PxA	1.91 ± 0.25	apparent At %^a^	24.4	30.5	0.0	5.8	39.3
		overlayer At %^b^		80	0.0	15.3	4.7
		theor. At %^c^		70	0.0	23.3	6.7
PAAm-*g*[4]-PFPA	1.4 ± 0.18	apparent At %^a^	27.0	22.7	2.8	5.0	42.6
		overlayer At %^b^		71.7	8.7	15.6	3.9
		theor. At %^c^		60	13.3	23.4	3.3

a Apparent atomic concentration including signals originating from the Si/SiO_2_ substrate.

b Normalized atomic concentration for the deposited adhesion promoter after subtraction of the substrate contributions from Si and SiO_2_.

c Theoretically calculated atomic composition.

Quantitative XPS verified the successful assembly of the adhesion promoting layers. The main differences observed were in the fluorine (F) and carbon (C) content of the various structures. Fluorine was not present in the PAAm-*g*-PxA composition and therefore was not detected. Additionally, the F atomic percentage (at %) of PAAm-*g*-PFPxA and PAAm-*g*-PFPA was double that of PAAm-*g*-DFPxA, as they contain four fluorine atoms versus two, respectively. Regarding the C content, the value of the PAAm-*g*-PFPA was lower than for the other three compositions, which had comparable values, as it contains a shorter linker in the para position confirming the thinner overlayer as measured by ELM. The high-resolution C 1s spectrum for the four adhesion promoter films can be deconvoluted into three components assigned to aliphatic carbon (C–C) at 285 eV, carbon bound to one N or oxygen and carbon bound to fluorine (C–F), which is found at the same binding energy as the amide carbon (C=O). The measured peak areas for the three components were in good agreement with the theoretically stoichiometric calculated numbers for the synthesized adhesion promoters with aryl azide grafting ratio of 4. The main deviation originated from an excess in the aliphatic peak, which can be explained by some residual atmospheric contamination. The carbon component assigned to the aromatic C–F groups at 287.8 eV scaled with the same pattern as expected for the amount of F present in the molecules (highest for PFPxA and PFPA containing adhesion promoters with four F per aryl azide, followed by DFPxA with only two F per aryl azide and lowest for PxA without F atoms). Overall, the elemental analysis supported the successful deposition of the four adhesion promoters on the SiO_2_ substrates (Tables 1 and 2).

**Table 2:** XPS C 1s High Resolution Spectra Deconvoluted into Three Carbon Components^a^

adhesion promoter		relative peak area (%)		
peak assignment		C–C	C–N, C–O	C–F, C= O
binding energy		285 ± 0.0 (eV)	286.1 ± 0.2 (eV)	287.8 ± 0.3 (eV)
PAAm-g[4]-DFPxA	At %^b^	63.6	24.6	11.8
	theor. At %^c^	66.7	24.2	9.1
PAAm-g[4]-PFPxA	At %^b^	58.6	22.9	18.5
	theor. At %^c^	62.8	22.9	14.3
PAAm-g[4]-PxA	At %^b^	67.8	25.2	7.0
	theor. At %^c^	71.0	25.8	3.2
PAAm-g[4]-PFPA	At %^b^	58.7	22.5	18.8
	theor. At %^c^	64.5	19.4	16.1

a The peak areas of the three components are compared to theoretically calculated ones for the corresponding aryl azide grafting ratios.

b Apparent atomic concentration of the three observed carbon components. Aliphatic carbon (C–C), carbon bound to nitrogen or oxygen (C–N, C–O), carbon bound to F or amide (C–F, C= O).

c Theoretically calculated atomic composition from their respective stoichiometry for the three components.

### PVP Monolayer Formation

**Figure 8:**
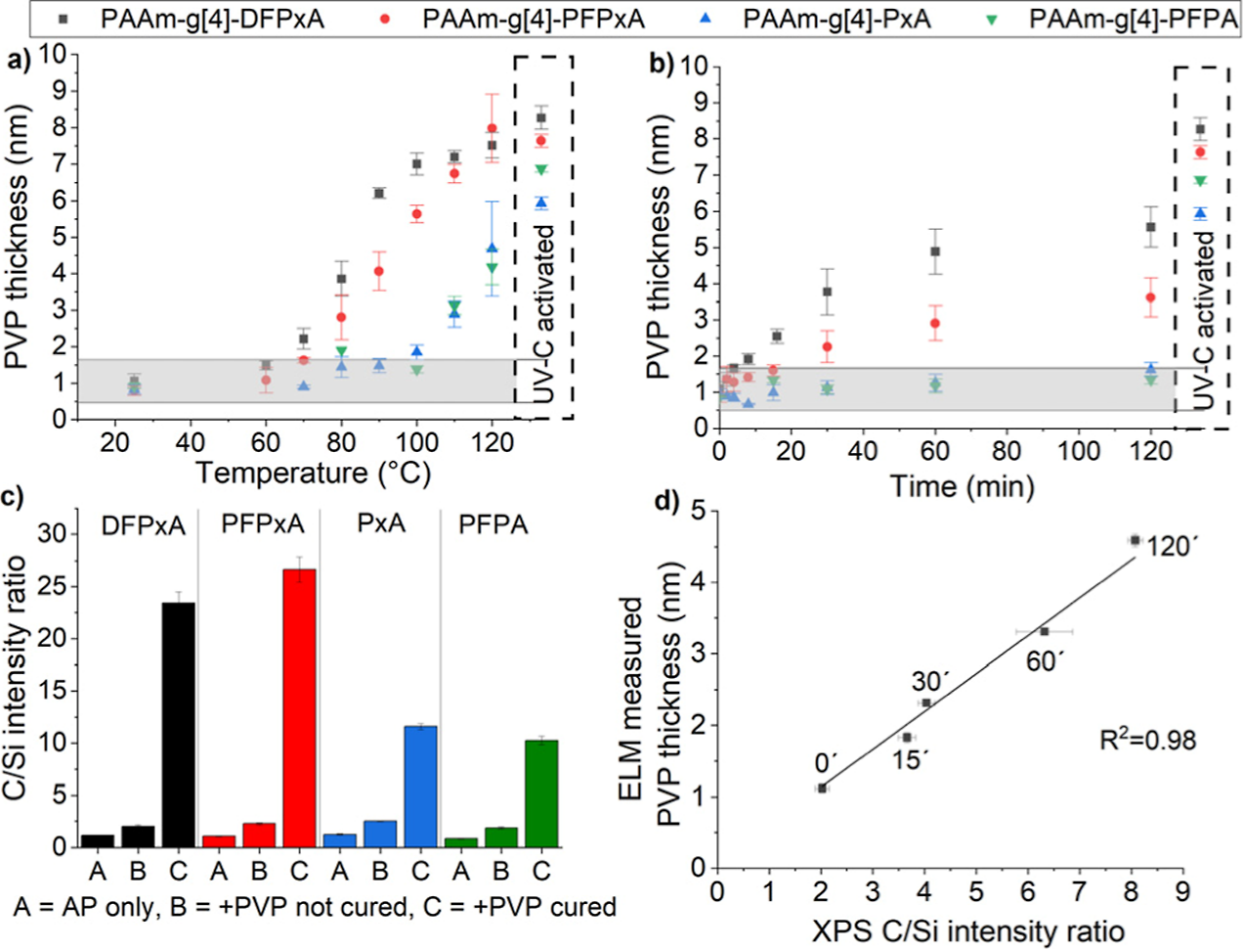
(a) Top-layer thickness of PVP after thermal activation of the samples containing an adhesion promoter with differently substituted reactive azides. On the right side, the thickness of the UV-C immobilized films is presented, demonstrating the maximum monolayer coverage of PVP for each adhesion promoter. The grey area indicates the 3× noise level of the uncured control samples where attachment is insignificant and thus, effective attachment of PVP has been defined as the thickness which is greater than 3× the deviation of level from the control samples. The temperature profile for 30 min activation displays increasing binding with increasing temperature and the order of activation agrees with the expected trend from low to high activation energy. (b) The time profile at 80 °C activation displays increasing binding with increasing exposure time. Again, the thickness of UV-C activated samples are plotted at the right as reference (c) comparative C/Si intensity ratio measured by XPS for samples coated with the four different adhesion promoters (AP), and additionally with PVP as top coating both uncured and cured at 120 °C for 30 min, agree with the ELM measurements. (d) Correlation of ELM thickness measurements and XPS C/Si intensity ratio of samples coated with PAAm-*g*-DFPxA adhesion promoter and PVP as top coating, cured at 80 °C for different time intervals. The fitted linear regression line shows an excellent correlation between the thickness values measured by ellipsometry (ELM) and the C/Si intensity ratio measured by XPS (*R*^2^ = 0.98).

For the quantitative characterization of the activation and successful covalent binding of the adhesion promoters with a polymeric coating, ELM was used to measure the dry layer thickness. Briefly, a 2-layer coating approach was used for the sample preparation, consisting of the assembly of a PAAm-*g*-arylazide adhesion promoter monolayer on SiO_2_ substrates forming strong ionic bonds, followed by coating of a polyvinylpyrrolidone (PVP) film that cannot react with the SiO_2_ surface and was used as model polymer known for its non-fouling properties.^24,31,32^ Provided that the adhesion promoters are not activated, the deposited PVP film interacts only via weak Van der Waals interactions and can be simply washed off. The activation of the azide groups towards formation of highly reactive nitrenes was performed either with UV-C light (as a positive control) or temperature, while non-activated samples (25 °C) were used as a negative control. Thorough rinsing ensured that the final thin film consisted of only covalently bound polymers to the surface. Upon complete activation, the polymer monolayer is expected to be bound by covalent bonds and remains after rinsing. If only part of the azide groups is activated, only a sub-monolayer of the polymer film is formed, explaining the increase in the observed apparent layer thickness by ELM with either increasing temperature or increasing curing time. A similar effect was achieved by Sterner et al.^31^ where partial curing by controlled UV-C illumination was used to form chemical polymer gradient surfaces (Figure 8a,b).

On the uncured reference samples (25 °C), the PVP polymer layer was washed away except for a thin remaining physisorbed film in the order of 1 nm. In contrast, the increased PVP layer thickness of the UV-C activated samples that remained attached after rinsing verified the successful insertion of the generated nitrenes to the PVP film and represented the full monolayer coverage for each adhesion promoter as all azide groups were expected to have been activated. Interestingly, the lower immobilized thickness of the sample containing the PAAm-*g*[4]-PxA adhesion promoter supports the hypothesis that the absence of fluorine atoms as ortho substituents yields less efficient insertion reactions, since intermolecular reactions compete with the intramolecular counterparts.

Samples prepared with the 2-layer coating approach were thermally activated for 30 min between 60 and 120 °C (Figure 8a). The data from this temperature profile demonstrated that effective immobilization of the PVP film began at a temperature of approximately 70 °C for the adhesion promoter containing the DFPxA moiety (for lower temperatures the azide decomposition reaction will be very slow, and thus would require a much longer curing period for attachment with PVP). Accordingly, we observed a steep increase in thickness transitioning from 80 °C to 90 °C as more azide groups (–N_3_) are expected to get activated, depicted as well in the azide decomposition rate constant from the kinetic analysis. As the temperature increased further, the PVP thickness slowly saturated around 7–8 nm as the rate of azide decomposition was very high and enough of the azide groups had been decomposed during the 30 min heating period to bind a full PVP monolayer. The PVP layer thickness increased similarly with temperature for all the adhesion promoters, following the anticipated differences in the initiation temperatures. Effective attachment for the adhesion promoter containing the PFPxA moiety occurred at slightly higher temperatures, at roughly 80 °C and for both adhesion promoters containing the PxA or PFPA moiety at substantially higher temperatures, above 110 °C. The saturation regions for PxA and PFPA were not visible in the curves, and it is safe to assume that it would be at further elevated temperatures.

Additional samples were also compared after activation for various time points at 80 °C, the temperature identified as the lower limit where PAAm-*g*[4]-DFPxA and PAAm-*g*[4]-PFxA adhesion promoters displayed substantial activation after 30 min (Figure 8b). Regarding the time-profile, PAAm-*g*[4]-DFPxA was the first adhesion promoter that exhibited effective attachment with the PVP film at 80 °C. We observed a linear continuous increase up to approximately 50% of the full monolayer reached at 30 min curing, where significant increase in thickness was observed even after the first 15 min of heating. Exposing the samples to longer heating times resulted in increased thickness of the attached PVP layer that gradually leveled off. To achieve a full monolayer coverage at 80 °C, heating periods longer than 120 min would be required. Regarding the PAA-*g*[4]-PFPxA adhesion promoter, effective attachment of PVP was monitored after 30 min of curing, but only long heating times of 120 min resulted in 50% immobilization of the respective PVP monolayer. Thus, acquisition of a full monolayer would necessitate much longer heating periods. Additionally, for the other two adhesion promoters with extremely low reaction rates at 80 °C, the decomposition was negligible and therefore not observed within the specific time scales.

To strengthen our findings, and to evaluate the differences of the quantity of the attached PVP layer, samples coated with the different adhesion promoters and PVP were characterized by XPS, after thermal activation at 120 °C for 30 min, as the lowest suitable temperature where all four adhesion promoters showed substantial activation.^36^ Upon activation, samples containing the PAAm-*g*-DFPxA and PAAm-*g*-PFPxA as adhesion promoters showed approximately double C/Si ratio compared to the other two adhesion promoters, in agreement with the ELM result. At 120 °C, almost all azide groups from PAAm-*g*-DFPxA and PAAm-*g*-PFPxA were activated resulting in a saturated PVP thick layer, whereas the azides of PAAm-*g*-PxA and PAAm-*g*-PFPA were only incompletely activated resulting in a thinner PVP layer. The top coating of the uncured samples was almost nonexistent and thus washed away (Figure 8c). The XPS data align well with the thickness measurements from ELM, and the ELM film thickness correlated to the C/Si ratio measured by XPS for the time dependent activation of the PAAm-*g*[4]-DFPxA adhesion promoter (Figure 8d). An almost perfect agreement between the two data sets (ELM vs XPS C/Si) was obtained for samples cured for different time intervals at 80 °C.

## Conclusions

Here, we identified and successfully synthesized two novel molecules, DFPxA-NHS and PFPxA-NHS, alongside two already known compounds PxA-NHS and PFPA-NHS. All four molecules consist of differently substituted aryl azides that upon activation create highly reactive nitrenes, which can undergo C–H insertion reactions. To determine the activation temperatures of the four compounds, we combined the thermal analysis data obtained by TGA and MS. Furthermore, we connected the reaction kinetics to the activation energy as determined by DFT calculations, revealing excellent correlation between theory and practice, suggesting that DFT is a good predictor for the activation energy calculations of such molecules. The novelty of the synthesized compound DFPxA-NHS is that the activation of the azide occurs thermally already at temperatures below 70 °C. This is 50 °C lower than the commercially available and widely used PFPA, making it an appropriate candidate for low temperature adhesion promoters.

We then successfully synthesized bifunctional adhesion promoter compounds for versatile permanent immobilization of polymeric films by grafting the reactive azide containing compounds to a positively charged polymeric backbone for electrostatic self-assembly. This allowed us to identify the activation temperature of the different aryl azide containing adhesion promoters for effective attachment of PVP, a polymer without adhesive functionality. We expect that other polymers would be similarly attached at the same temperatures. The outcome fully aligns with the findings from the thermal characterization of the reactive compounds and the DFT calculations, indicating that DFPxA containing adhesion promoters activate and successfully covalently bind to polymer films already at ∼70 °C. To the best of our knowledge, thermal activation of existing adhesion promoting molecules containing an azide for efficient intermolecular insertion reactions occurs at temperatures higher than 100 °C. This will enable easy surface modification of multiple ready to use products, based on polymeric materials with low glass transition temperature or low melting point, such as closed structured microfluidic chips or catheters, materials with peculiar shapes or even light-blocking materials where light activation is challenging or impossible.

## Materials and Methods

### Materials/Standard Chemicals

#### Synthetic Procedures

For all experiments, ACS grade *N*,*N*-dimethylformamide, 2-propanol, ethanol, chloroform, toluene and ethyl acetate (Merck, Germany), methanol (VWR Chemicals, Switzerland) and hexanes (Thermo Scientific, Germany) were used. Ultrapure water (UPW) was produced from a PURELAB chorus 1 complete system (≥18 MΩ/cm, ELGA). 4-amino-3,5-difluorophenol was purchased from Manchester Organics (UK), 4-amino-2,3,5,6-tetrafluorophenol from A2B Chem (USA), 4-aminophenol from Sigma-Aldrich (Switzerland), methyl 4-bromobutyrate from abcr (Switzerland), sodium nitrite, sodium azide, trifluoro acetic acid, sodium hydroxide, *N*,*N*′-dicyclohexylcarbodiimide, *N*-hydroxysuccinimide from Sigma-Aldrich (Switzerland), and used as received.

#### NMR Measurements

Deuterated dimethyl sulfoxide (DMSO-*d*_6_) was purchased from Sigma-Aldrich (Switzerland).

Silicon wafers chips for coating experiments were obtained from Powatec GmbH Switzerland.

### Instrumentation and Characterization

#### DFT Computational Calculations

Transition state energy calculations for azide to singlet nitrene decomposition were performed by starting with a geometry optimization of the corresponding phenylazide using the B3LYP functional combined with 6-31G** basis set using the Gaussian 09 (Revision D.01) software package.^28^ All calculations were performed at a temperature of 0 K in vacuum. In a second step, the N–N_2_ distance was increased stepwise by 0.2 Å steps up to 2.4 Å and at each step a geometry optimization was performed (B3LYP functional combined with 6-31G** basis set). This yields a 1-dimensional potential energy curve depending on the RN–N_2_ bond distance from which the bond-cleavage energy for bond breaking to form the singlet nitrene R–N + N_2_ is obtained (Figure S3). To determine the relative energy of the generated singlet nitrene, the N–N_2_ distance was set fixed to 50 Å, a large enough distance to have no interaction between N_2_ and the generated nitrene since above 3 Å a plateau is reached, and a further energy optimization was performed with these coordinates.^28^

#### Thermogravimetric Analysis (TGA)/Differential Scanning Calorimetry

Thermal characterization measurements were performed using a Mettler Toledo TGA/DSC 3+ thermal analysis system under atmospheric pressure with a flow rate of 50 mL/min. In a typical experiment, ∼10 mg of the sample was placed in an alumina crucible for measurement. Both weight-loss and heatflow data over a temperature range of 25–200 °C were measured to identify the changes in the weight/heatflow of the sample. The heating rate was set at 10 °C/min until 55 °C where the sample stayed for 30 min to equilibrate, and then the heating rate was decreased to 1 °C/min up to 200 °C. In that way, increased resolution of the generated curves in the temperature region of interest could be acquired compared to higher heating rates, namely the region where the decomposition reaction of the aryl azides takes place.

For the data analysis, weight loss data were preferred over the heat flow data as the focus lied in identifying temperature regions associated with mass loss due to azide decomposition, and not in phase transitions. It should also be noted that up to a temperature of 50 °C detected mass loss is due to evaporation of residual solvents and not due to azide decomposition. The data are plotted as *xyy* graphs, where the temperature is plotted in the *x*-axis, in the left *y*-axis the weight in percentage and in the right *y*-axis the first derivative of the weight (Figures S6 and S7). The first derivative curve is an essential tool to determine the point of initial and/or greatest change on the weight loss curve, since even minor changes can be identified.^37^

#### Mass Spectroscopy over Increasing Temperature

In this set of experiments, we gathered information for many different molecular weight fragments as potential decomposition products. Besides *m*/*z* = 4 corresponding to the carrier gas helium, no other molecular fragment gave MS signal until the system stabilization temperature of 55 °C. The first MS signal when the temperature further increased was measured for *m*/*z* 28, namely N_2_. In that way, it is corroborated that the primary chemical group to decompose of each structure is the aromatic azide as expected. The point where N_2_ starts emitting can be set as the initial decomposition point of the azide group.^38^

Prior to the measurements the samples were thoroughly dried under high vacuum. MS measurements were performed to identify the molecular fragments and the volatile groups that are emitted upon thermal decomposition at increasing temperatures. Thermal decomposition measurements were carried out using an AutoChem II 2920 system (Micromeritics Instrument Corporation) for the accurate control of temperature connected to an MKS Cirrus 2 quadrupole MS. In a typical experiment, ∼10 mg of sample was placed in a U-shape quartz tube (i.d. 10 mm). The sample was supported on a plug of quartz wool and covered the entire cross section of the quartz tube. The temperature inside the quartz tube was measured and controlled using a type K thermocouple that was placed ∼2 mm above the sample.

The sample was heated to 40 °C under helium flow (20 mL/min) and held there for 30 min to stabilize both the thermal conductivity detector (TCD) and the MS. Subsequently, the sample was heated to 55 °C at a rate of 10 °C/min where it was held for another 30 min for residual solvent evaporation, followed by heating to 200 °C at a rate of 1 °C/min. The following mass-to-charge ratios (*m*/*z*) were acquired to analyze the potential gaseous decomposition products: 2, 4, 14, 16, 18, 20, 28, 32, 40, 44, 49, 51, 84, 86.

#### Kinetic Analysis

The decomposition of an azide towards nitrene formation is a unimolecular reaction and should follow first-order kinetics and thus, the reaction rate is proportional to the concentration of the azide. Moreover, the proportion of molecules with kinetic energy larger than the activation energy is determined only by the reaction temperature.

The reaction rate *r* in a unimolecular reaction calculating the consumption of a substrate with concentration [*A*] and the reaction rate constant *k* is shown by eq 1.
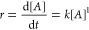
1

According to the Arrhenius first order reaction model, it is possible to write the equation in a non-exponential form as it is more straightforward to use and interpret graphically. By taking the natural logarithm on both sides, the final form is a relation of ln *k* ∼ 1/*T*. The general form of a first-order reaction after rearrangements and integration is given by eq 2
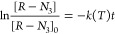
2where [*R*–*N*_3_] is the concentration of the reactant (azide) at each time point, [*R*–*N*_3_]_0_ is the initial concentration of the reactant, *k*(*T*) is the reaction rate constant at a given temperature *T* and *t* is time.^38^ The equation is in the form of a straight line and by plotting the natural logarithm of the measured reaction rate over the inverse temperature and by linear fitting of the data points, it is possible to acquire interpolated data points of the reaction rate at different temperatures and for the different reactive compounds. (Figures S5, S8 and Table S1). The half-life (*t*_1/2_) in Figure 5 (eq 3), was calculated based on eq 2, where [*R*–*N*_3_] = [*R*–*N*_3_]_0_/2
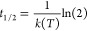
3

#### Spectroscopic Ellipsometry

To determine the thickness of the adsorbed polymer layer, spectroscopic ellipsometry (ELM, M-2000F, J.A. Woollam Co., Lincoln, Ne, USA) measurements were collected at a 70° angle of incidence in the spectral range 370–1000 nm. A three-layer model [(1) Si, (2) Si oxide, (3) polymer adlayer (Cauchy function, *A*_*n*_ = 1.45, *B*_*n*_ = 0.01, *k* = 0)] was used to fit the data using the software Complete EASE from J.A. Woollam Co. Measurements were made first on the plasma-cleaned silicon wafer, second after incubation of adhesion promoters, and third after PVP deposition, curing and rinsing.

#### X-ray Photoelectron Spectroscopy

The chemical composition of the polymer films was determined by means of X-ray photoelectron spectroscopy (XPS). Measuring conditions for XPS were as previously reported by Weydert et al.^39^ All spectra were recorded using a PHI5000 Versa probe (ULVAC-PHI, INC., Chigasaki, Japan). The spectrometer is equipped with a 180° spherical-capacitor energy analyzer and a multichannel detection system with 16 channels. Spectra were acquired at a base pressure of 5 × 10^–8^ Pa using a focused, scanning, monochromatic Al K α source (1486.6 eV) with a spot size of 200 μm and 47.6 W power. The instrument was run in FAT analyzer mode, with electrons emitted at 45° to the surface normal. The pass energies used for survey scans were 187.85 and 46.95 eV for detailed spectra for the following peaks: Si 2p, C 1s, O 1s, N 1s, and F 1s. The full width at half-maximum (fwhm) of this setup is < 0.8 eV for Ag 3d_5/2_. The XPS spectra were evaluated using CasaXPS (version 2.3.16). Binding energies are referenced relative to the hydrocarbon peak (from residual contamination in the case of the clean surfaces or the –CH_2_–CH_2_–CH_2_– contribution of the polymers), set at a binding energy (BE) of 285.0 eV. Normalized atomic percent (atom %) concentrations were calculated from the detailed spectra of each element present on the surface, corrected by the appropriate relative sensitivity factors (RSFs), the asymmetry parameter,^40^ the transmission function of the spectrometer, and inelastic mean free paths (IMFPs). The photoionization cross sections are normalized to C 1s according to Scofield,^41^ except for Si 2p, where an experimentally determined factor of 1.06 was used, measured on a clean SiO_2_ quartz reference material. This value is higher than the tabulated value.

#### Nuclear Magnetic Resonance

To confirm the successful synthesis of each intermediate product of all the synthetic steps, ^1^H NMR and for all fluorinated compounds additionally ^19^F NMR spectroscopy was performed. Data were collected with a Neo 400 MHz with BBFO smart probe spectrometer (Bruker). The residual undeuterated solvent peaks were used for references (at 2.5 ppm for DMSO-*d*_6_ as well as at 0 ppm for TMS). The following abbreviations were used to denote multiplicities: s = singlet, d = doublet, t = triplet, m = multiplets and br = broad. Relative integration is reported in number of protons (H).

^13^C spectra were collected for compounds 1a–5a, 2b, 5b, 1c–5c. The weak intensity of fluorinated aromatic carbon signals did not allow to determine accurate C–F coupling constants.

All collected NMR spectra are found in the Supporting Information (Figures S9–S45).

#### Elemental Analysis

To confirm the elemental composition and purity of the 3 synthesized molecules, a Leco Truespec CHN Elemental Analyzer equipped with Infrared Spectrometry and Thermal Conductivity (N_2_) analyzers was used. Oxygen was measured as carbon dioxide by an infrared detector and nitrogen by a thermal conductivity detector. In the DFPxA-NHS and PFPxA-NHS samples, the measurement of oxygen was not possible due to the presence of fluorine, and the value of the theoretical composition was calculated instead.

#### Attenuated Total Reflectance–Fourier-Transform Infrared Spectroscopy

ATR–FTIR was measured to confirm the formation of the azide group in the molecule. The azide group shows a characteristic IR frequency at ∼2100 (cm^–1^). The spectra were acquired on a PerkinElmer ATR Spectrum TWO equipped with an UATR single reflection diamond. Sample powders were pressed onto the diamond crystal with a pressure arm and a spectrum was recorded between 500 and 4000 cm^–1^ averaging over 32 scans. ATR spectra for the synthesized compounds **5a**, **5b** and **5c** are found in Supporting Information (Figures S44–S46).

#### Temperature-Controlled Fourier-Transform Infrared Spectroscopy

A Varian 640 Fourier transform infra-red spectrometer (FTIR) was used to acquire attenuated total reflection Fourier-transform infrared (ATR–FTIR) spectra, that were recorded at wavenumber range of 600 to 4000 cm^–1^. The instrument was equipped with a Golden Gate-diamond ATR with temperature control (electrical heater up to 200 °C) for solid samples. The powder sample was pressed onto an ATR diamond crystal and the temperature was increased to the desired value. The sample was left for 5 min to equilibrate, and an additional 15 min were allocated for the sample to undergo thermal decomposition reactions. After the 15 min, a spectrum was recorded. This procedure was repeated until completion of the target temperatures. After the collection of all the different spectra a compilation of them yielded the desired cumulative graph, where the decomposition of the azide over increasing temperature and time is plotted (Figure 4).

### Synthetic Procedures

The general protocol for the synthesis of the three compounds requires that the first reaction step was a nucleophilic substitution in basic environment. As a second step, the aromatic amine was converted into an aromatic azide in acidic environment,^42^ through diazotization of the aryl amine and subsequent reaction with sodium azide. As a third step, the ester was hydrolyzed to form the respective carboxylic acid. The fourth and last step of the synthetic protocol, consists of the formation of an *N*-hydroxysuccinimide ester by reaction of the carboxylic group with *N*-hydroxysuccinimide (NHS).^43^ The successful synthesis of each step was verified by NMR of the isolated intermediate compounds, as well as for the quantitative calculation of the purity of the final isolated molecules (96.7%, 90%, 95% for **5a**, **5b**, **5c** respectively). To verify the formation of the azide, Fourier-transform infrared spectroscopy (FTIR) was performed, displaying a characteristic peak of the bond stretching at ∼2130 cm^–1^ frequency. Elemental analysis was performed, to validate the elemental composition of the final active-ester functionalized aryl azides.

#### Synthesis of DFPxA-NHS (**5a**)

##### Synthesis of Methyl-4-(4-amino-3,5-difluorophenoxy) butanoate (**2a**)

4-amino-3,5-difluorophenol (**1a**) (1000 mg, 6.89 mmol) was dissolved in *N*,*N*-dimethylformamide (10 mL). Methyl 4-bromobutyrate (957 μL, 7.58 mmol) was added dropwise while stirring followed by the addition of potassium carbonate (1905 mg, 13.78 mmol). The solution was allowed to stir at 85 °C in heated oil bath for 4 h. Then, ultrapure water (30 mL) was added to quench the reaction and dissolve the inorganic reagents, and the reaction was extracted with ethyl acetate (2 × 30 mL). The solvent of the extract was evaporated in vacuo (40 °C) to yield a dark brown/red oil. The crude methyl-4-(4-amino-3,5-difluorophenoxy) butanoate (**2a**) (∼95% functionalization of –OH determined by ^1^H NMR and the presence of the residual aromatic peaks of the starting material after reaction) is stored in the dark below room temperature (<−10 °C) and used for the next synthetic step without further purification (yield 2269 mg, 134% calculated with excess DMF).

^1^H NMR (400 MHz, DMSO-*d*_6_): δ (ppm), 6.65–6.52 (m, 2H, Ph–H), 4.6 (s, 2H, NH_2_), 3.87 (t, 2H, O–C**H**_**2**_–), 3.59 (s, 3H, C**H**_**3**_), 2.42 (t, 2H, –C**H**_**2**_–COO–CH_3_), 1.9 (p, 2H, –C**H**_**2**_–). ^19^F NMR (376.5 MHz, DMSO-*d*_6_): δ (ppm), −129.7 (s, 2F). ^13^C NMR (100 MHz, DMSO-*d*_6_): δ (ppm), 173.42, 153.2 (d, *J* = 11.5 Hz), 150.86 (d, *J* = 11.5 Hz), 149.01 (t, *J* = 12.8 Hz), 119.192 (t, *J* = 17.3 Hz), 99.32 – 99.06 (m), 67.78, 51.77, 30.3, 24.53.

##### Synthesis of Methyl-4-(4-azido-3,5-difluorophenoxy) butanoate (**3a**)was Performed along a Protocol from Nunes et al^42^

Methyl-4-(4-amino-3,5-difluorophenoxy) butanoate (**2a**) (1690 mg, 6.89 mmol) was dissolved in trifluoroacetic acid (10 mL) in an ice bath. Sodium nitrite (570.6 mg, 8.27 mmol) was added portion wise while stirring for 5 min. During this time the color changed initially to dark green followed by a change to dark red after 5 min. After addition of sodium azide (537 mg, 8.27 mmol), production of foam was observed, explained by the release of N_2_ as expected from the reaction mechanism. The solution was allowed to stir for an additional 1 h at room temperature. Then, ultrapure water (30 mL) was added, and the reaction mixture was extracted with diethyl ether (3 × 30 mL), washed well with ultrapure water and saturated aqueous NaHCO_3_. The extract was dried over anhydrous magnesium sulphate and the solvent evaporated in vacuo (40 °C) to yield a dark red/brown oil. This residue was purified by column chromatography (silica gel; eluent: hexane/diethyl ether, 3:1) to obtain the desired methyl-4-(4-azido-3,5-difluorophenoxy) butanoate (**3a**) as a dark orange oil (yield: 700 mg, 38%). The product is stored in the dark below room temperature (<−10 °C).

^1^H NMR (400 MHz, DMSO-*d*_6_): δ (ppm), 6.9–6.82 (m, 2H, Ph–H), 3.99 (t, 2H, O–C**H**_**2**_–), 3.59 (s, 3H, C**H**_**3**_), 2.44 (t, 2H, –C**H**_**2**_–COO–CH_3_), 1.94 (p, 2H, –C**H**_**2**_–). ^19^F NMR (376.5 MHz, DMSO-*d*_6_): δ (ppm), −122.4 (s, 2F). ^13^C NMR (100 MHz, DMSO-*d*_6_): δ (ppm), 172.86, 156.74 (d, *J* = 7.6 Hz), 156.17 (t, *J* = 13.4 Hz), 154.30 (d, *J* = 7.6 Hz), 108.73 (t, *J* = 15.1 Hz), 100.18 – 99.31 (m), 67.72, 51.33, 29.69, 23.82.

##### Synthesis of (4-azido-3,5-difluorophenoxy) Butanoic Acid (**4a**)

Methyl-4-(4-azido-3,5-difluorophenoxy) butanoate (**3a**) (700 mg, 2.58 mmol) was dissolved in methanol (7 mL). Sodium hydroxide (181.7 mg, 4.54 mmol) was dissolved in 2 mL ultrapure water and added dropwise until pH∼10. The mixture was let to react by stirring overnight at room temperature, capped, and in the dark. The following day, HCl (2M) was added until pH∼1. The solvent methanol was evaporated in vacuo (50 °C). Then, ultrapure water (8 mL) was added, and the reaction mixture was extracted with chloroform (3 × 10 mL). The extract was dried over anhydrous magnesium sulphate and the solvent evaporated in vacuo (50 °C) to obtain the desired (4-azido-3,5-difluorophenoxy) butanoic acid (**4a**) as a dark red solid (yield: 538 mg, 81%). The product is stored in the dark below room temperature (<−10 °C) to minimize its decomposition.

^1^H NMR (400 MHz, DMSO-*d*_6_): δ (ppm), 12.15 (s, 1H, OH), 6.92–6.83 (m, 2H, Ph–H), 3.99 (t, 2H, O–C**H**_**2**_–), 2.35 (t, 2H, –C**H**_**2**_–COO–CH_3_), 1.9 (p, 2H, –C**H**_**2**_–). ^19^F NMR (376.5 MHz, DMSO-*d*_6_): δ (ppm), −122.4 (s, 2F). ^13^C NMR (100 MHz, DMSO-*d*_6_): δ (ppm), 173.94, 156.75 (d, *J* = 7.6 Hz), 156.23 (t, *J* = 13.4 Hz), 154.30 (d, *J* = 7.6 Hz), 108.7 (t, *J* = 15.1 Hz), 101.12 – 98.80 (m), 67.84, 29.87, 23.84.

##### Synthesis of (4-azido-3,5-difluorophenoxy) Butanoic Acid – *N*-hydroxysuccinimide Ester (**5a**)

(4-azido-3,5-difluorophenoxy) butanoic acid (**4a**) (530 mg, 2.06 mmol) was dissolved in dichloromethane (6 mL). *N*-hydroxysuccinimide (249 mg, 2.16 mmol) was added. N,N′-dicyclohexylcarbodiimide (446.4 mg, 2.16 mmol) after dissolved in 2 mL dichloromethane, was added dropwise to the above mixture. The mixture was stirred overnight at room temperature, capped, and in the dark. The following day, dicyclohexylurea produced during the overnight reaction was filtered from the mixture, and the filtrate solution was then filtered over celite. The solvent of the final filtrate solution was evaporated in vacuo (50 °C) to obtain the desired (4-azido-3,5-difluorophenoxy) butanoic acid *N*-hydroxy-succinimide ester (**5a**) as a brown solid (yield: 704 mg, 96% and purity = 96.7% calculated by ^1^H NMR). Exposure to light or keeping the product at room temperature may results in degradation and, therefore, it was stored in the dark below room temperature (<−10 °C) to minimize its decomposition.

^1^H NMR (400 MHz, DMSO-*d*_6_): δ (ppm), 6.96–6.86 (m, 2H, Ph–H), 4.06 (t, 2H, O–C**H**_**2**_–), 2.82 (t, 2H, –C**H**_**2**_–COO–), 2.81 (s, 4H, C_4_H_4_NO_2_), 2.05 (p, 2H, –C**H**_**2**_–). ^19^F NMR (376.5 MHz, DMSO-*d*_6_): δ (ppm), −122.3 (s, 2F). ^13^C NMR (100 MHz, DMSO-*d*_6_): δ (ppm), 170.23, 168.68, 156.71 (d, *J* = 7.7 Hz), 156.04 (s), 154.27 (d, *J* = 7.5 Hz), 100.34 – 99.31 (m), 67.15, 26.99, 25.44, 23.63. IR (ATR diamond crystal) wavenumber (cm^–1^): 642 (w), 809 (s), 840 (s), 875 (s), 911 (w), 1048/1073 (sbr), 1159 (s), 1208 (s), 1302 (w), 1361 (w), 1508 (w), 1579 (w), 1639 (w), 1735 (s), 1783 (w), 2100 (w), 2135 (s, as stretch N_3_) and 2937 (m). Elemental analysis %: Anal. Calcd for C_14_H_12_N_4_F_2_O_5_: C, 47.46; H, 3.41; N, 15.82; F, 10.73; O, 22.58. Found: C, 47.92; H, 3.72; N, 14.97; F, 10.18.

#### Synthesis of PFPxA-NHS (**5b**)

##### Synthesis of Methyl-4-(4-amino-2,3,5,6-tetrafluorophenoxy) butanoate (**2b**)

4-amino-2,3,5,6-tetrafluorophenol (**1b**) (700 mg, 3.87 mmol) was dissolved in *N*,*N*′-dimethylformamide (7 mL) and the solution was stirred until complete dissolution. Methyl 4-bromobutyrate (536.8 μL, 4.25 mmol) was added dropwise while stirring followed by the addition of potassium carbonate (1068.5 mg, 7.73 mmol). The solution was allowed to stir at 100 °C in a heated oil bath for 4 h. Then, ultrapure water (30 mL) was added to quench the reaction and dissolve the inorganic reagents, and the reaction was extracted with diethyl ether (2 × 30 mL). The solvent of the extract was evaporated in vacuo (40 °C) to yield a dark brown oil. The crude methyl-4-(4-amino-2,3,5,6-tetrafluorophenoxy) butanoate (**2b**) (complete functionalization of –OH determined by ^1^H NMR and the absence of any residual peaks of the starting material after reaction) is stored in the dark below room temperature (<−10 °C) and used for the next synthetic step without further purification (yield 1437 mg, 132% calculated with excess DMF).

^1^H NMR (400 MHz, DMSO-*d*_6_): δ (ppm), 5.64 (s, 2H, NH_2_), 4.01 (t, 2H, O–C**H**_**2**_–), 3.59 (s, 3H, C**H**_**3**_), 2.47 (t, 2H, –C**H**_**2**_–COO–CH_3_), 1.9 (p, 2H, –C**H**_**2**_–). ^19^F NMR (376.5 MHz, DMSO-*d*_6_): δ (ppm), −160.25 (d, 2F), −162.4 (d, 2F). ^13^C NMR (100 MHz, DMSO-*d*_6_): δ (ppm), 172.85, 142.87 (m), 140.51 (m), 137.11 (m), 134.77 (m), 126.54 – 121.95 (m), 74.31, 51.32, 29.40, 24.68.

##### Synthesis of Methyl-4-(4-azido-2,3,5,6-tetrafluorophenoxy) butanoate (**3b**)was Performed along a Protocol from Nunes et al^42^

Methyl-4-(4-amino-2,3,5,6-tetrafluorophenoxy) butanoate (**2b**) (1087 mg, 3.87 mmol) was dissolved in trifluoroacetic acid (10 mL) in an ice bath. Sodium nitrite (320 mg, 4.64 mmol) was added portion wise while stirring for 5 min. During this time the color changed initially to dark green followed by a change to dark red after 5 min. After addition of sodium azide (301.6 mg, 4.64 mmol), production of foam was observed, explained by the release of N_2_ as expected from the reaction mechanism. The solution was allowed to stir for an additional 1 h at room temperature. Then, ultrapure water (30 mL) was added, and the reaction mixture was extracted with diethyl ether (3 × 30 mL), washed well with ultrapure water and saturated aqueous NaHCO3. The extract was dried over anhydrous magnesium sulphate and the solvent evaporated in vacuo (40 °C) to yield a dark red oil. This residue was purified by column chromatography (silica gel; eluent: hexane/diethyl ether, 3:1) to obtain the desired methyl-4-(4-azido-2,3,5,6-tetrafluorophenoxy) butanoate (**3b**) as an orange/red oil (yield: 737 mg, 62%). The product was stored in the dark below room temperature until further use (<−10 °C).

^1^H NMR (400 MHz, DMSO-*d*_6_): δ (ppm), 4.2 (t, 2H, O–C**H**_**2**_–), 3.6 (s, 3H, C**H**_**3**_), 2.48 (t, 2H, –C**H**_2_–COO–CH_3_), 1.95 (p, 2H, –C**H**_**2**_–). ^19^F NMR (376.5 MHz, DMSO-*d*_6_): δ (ppm), −153.35 (d, 2F), −157.61 (d, 2F).

##### Synthesis of (4-azido-2,3,5,6-tetrafluorophenoxy) Butanoic Acid (**4b**)

Methyl-4-(4-azido-2,3,5,6-tetrafluorophenoxy) butanoate (**3b**) (737 mg, 2.4 mmol) was dissolved in methanol (7 mL). Sodium hydroxide (168.9 mg, 4.22 mmol) was dissolved in 1 mL ultrapure water and added dropwise until pH∼10. The mixture was let to react by stirring overnight at room temperature, capped, and in the dark. The following day, HCl (2M) was added until pH∼1. The solvent methanol was evaporated in vacuo (50 °C). Then, ultrapure water (10 mL) was added, and the reaction mixture was extracted with chloroform (3 × 10 mL). The extract was dried over anhydrous magnesium sulphate and the solvent evaporated in vacuo (50 °C) to obtain the desired (4-azido-2,3,5,6-tetrafluorophenoxy) butanoic acid (**4b**) as a yellow/orange solid (yield: 690 mg, 98%). The product is stored in the dark below room temperature (<−10 °C) to minimize its decomposition.

^1^H NMR (400 MHz, DMSO-*d*_6_): δ (ppm), 12.16 (s, 1H, –OH), 4.2 (t, 2H, O–C**H**_**2**_–), 2.38 (t, 2H, –C**H**_**2**_–COO–CH_3_), 1.91 (p, 2H, –C**H**_**2**_–). ^19^F NMR (376.5 MHz, DMSO-*d*_6_): δ (ppm), −153.32 (d, 2F), −157.53 (d, 2F).

##### Synthesis of (4-azido-2,3,5,6-tetrafluorophenoxy) Butanoic Acid-*N*-hydroxy-succinimide Ester (**5b**)

(4-azido-2,3,5,6-tetrafluorophenoxy) butanoic acid (**4b**) (680 mg, 2.32 mmol) was dissolved in dichloromethane (6 mL). *N*-hydroxy-succinimide (267.2 mg, 2.32 mmol) was added. *N*,*N*′-di-cyclohexyl-carbodiimide (488.7 mg, 2.37 mmol) after dissolved in 1 mL dichloromethane, was added dropwise to the mixture. The mixture was stirred overnight at room temperature, capped, and in the dark. The following day, dicyclohexylurea produced during the overnight reaction was filtered out from the mixture, and the filtrate was filtered a second time over celite. The solvent of the final filtrate was evaporated in vacuo (50 °C) to obtain the desired (4-azido-2,3,5,6-tetrafluorophenoxy) butanoic acid-*N*-hydroxy-succinimide ester (**5b**) as a brown solid (yield: 862 mg, 96%). Exposure to light or keeping the product at room temperature may result in degradation and, therefore, it was stored in the dark below room temperature (<−10 °C) to minimize its decomposition.

^1^H NMR (400 MHz, DMSO-*d*_6_): δ (ppm), 4.26 (t, 2H, O–C**H**_**2**_–), 2.86 (t, 2H, –C**H**_**2**_–COO–), 2.81 (s, 4H, C_4_**H**_**4**_NO_2_), 2.06 (p, 2H, –C**H**_**2**_–). ^19^F NMR (376.5 MHz, DMSO-*d*_6_): δ (ppm), −153.3 (d, 2F), −157.39 (d, 2F). ^13^C NMR (100 MHz, DMSO-*d*_6_): δ (ppm), 170.13, 168.50, 151.78 (m), 142.4–139.4 (m), 133.18 (m), 73.78, 26.54, 25.42, 24.63. IR (ATR diamond crystal) wavenumber (cm^–1^): 897 (w), 966 (w), 1000 (w), 1075 (w), 1107 (w), 1207 (s), 1314 (w), 1374 (w) 1498 (s), 1728 (s), 1781 (w), 1808 (w), 2129 (s, as stretch N_3_) and 2954 (m). Elemental analysis %: Anal. Calcd for C_14_H_10_F_4_N_4_O_5_: C, 43.09; H, 2.58; N, 14.36; F, 19.47; O, 20.5. Found: C, 46.13; H, 3.68; N, 14.01; F, 15.00.

#### Synthesis of PxA-NHS (**5c**)

##### Synthesis of Methyl-4-(4-amino-phenoxy) butanoate (**2c**)

4-amino-phenol (**1c**) (1000 mg, 9.16 mmol) was dissolved in *N*,*N*′-dimethylformamide (10 mL) and the solution was stirred until complete dissolution. Methyl 4-bromobutyrate (1272.5 μL, 10.8 mmol) was added dropwise while stirring followed by the addition of potassium hydroxide (1028.3 mg, 18.33 mmol). The solution was allowed to stir at 60 °C in a heated oil bath for 4 h. Then, ultrapure water (30 mL) was added to quench the reaction and dissolve the inorganic reagents, and the reaction was extracted with diethyl ether (2 × 30 mL). The solvent of the extract was evaporated in vacuo (40 °C) to yield a dark brown/red oil. The crude methyl-4-(4-amino-phenoxy) butanoate (**2c**) is stored in the dark below room temperature (<−10 °C) and used for the next synthetic step without further purification (yield 1410 mg, 74% including some DMF).

^1^H NMR (400 MHz, DMSO-*d*_6_): δ (ppm), 6.65–6.58 (m, 2H, Ph–H), 6.51–6.46 (m, 2H, Ph–H), 4.58 (s, 2H, NH_2_), 3.82 (t, 2H, O–C**H**_**2**_–), 3.59 (s, 3H, C**H**_**3**_), 2.43 (t, 2H, –C**H**_**2**_–COO–CH_3_), 1.89 (p, 2H, –C**H**_**2**_–). ^13^C NMR (100 MHz, DMSO-*d*_6_): δ (ppm), 173.10, 162.31, 149.72, 142.46, 115.38, 114.90, 66.95, 51.29, 30.01, 24.43.

##### Synthesis of Methyl-4-(4-azido-phenoxy) butanoate (**3c**)was Performed along a Protocol from Nunes et al^42^

Methyl-4-(4-amino-phenoxy) butanoate (**2c**) (1324.27 mg, 6.33 mmol) was dissolved in trifluoroacetic acid (13 mL) in an ice bath. Sodium nitrite (526.37 mg, 7.6 mmol) was added portion wise while stirring for 5 min. During this time the colour changed initially to dark green followed by a change to dark red after 5 min. After addition of sodium azide (493.44 mg, 7.6 mmol), production of foam was observed, explained by the release of N_2_ as expected from the reaction mechanism. The solution was allowed to stir for an additional 1 h at room temperature. Then, ultrapure water (30 mL) was added, and the reaction mixture was extracted with ethyl acetate (3 × 30 mL). The combined organic fractions were washed well with ultrapure water and saturated aqueous NaHCO3. The extract was dried over anhydrous magnesium sulphate and the solvent evaporated in vacuo (40 °C) to yield a dark red oil. This residue was purified by column chromatography (silica gel; eluent: hexane/diethyl ether, 3:1) to obtain the desired methyl-4-(4-azido-phenoxy) butanoate (**3c**) as a red oil (yield: 220 mg, 15%). The product is stored in the dark below room temperature (<−10 °C).

^1^H NMR (400 MHz, DMSO-*d*_6_): δ (ppm), 7.6–6.82 (m, 2H, Ph–H), 6.99–6.93 (m, 2H, Ph–H), 3.96 (t, 2H, O–C**H**_**2**_–), 3.59 (s, 3H, C**H**_**3**_), 2.46 (t, 2H, –C**H**_**2**_–COO–C**H**_**3**_), 1.95 (p, 2H, –C**H**_**2**_–). ^13^C NMR (100 MHz, DMSO-*d*_6_): δ (ppm), 172.99, 155.96, 131.46, 120.16, 115.90, 66.85, 51.32, 29.88, 24.15.

##### Synthesis of (4-azido-phenoxy) Butanoic Acid (**4c**)

Methyl-4-(4-azido-phenoxy) butanoate (**3c**) (155 mg, 0.66 mmol) was dissolved in methanol (2 mL). Sodium hydroxide (155 mg, 0.66 mmol) was dissolved in 0.5 mL ultrapure water and added dropwise until pH∼10. The mixture was stirred overnight at room temperature, capped, and in the dark. The following day, HCl (2M) was added until pH∼1. The solvent methanol was evaporated in vacuo (50 °C). Then, ultrapure water (10 mL) was added, and the reaction mixture was extracted with chloroform (3 × 10 mL). The extract was dried over anhydrous magnesium sulphate and the solvent evaporated in vacuo (50 °C) to obtain the desired (4-azido-phenoxy) butanoic acid (**4c**) as a red solid (yield: 97 mg, 67%). The product is stored in the dark below room temperature (<−10 °C) to minimize its decomposition.

^1^H NMR (400 MHz, DMSO-*d*_6_): δ (ppm), 7.06–7 (m, 2H, Ph–H), 7–6.94 (m, 2H, Ph–H), 3.96 (t, 2H, O–C**H**_**2**_–), 2.37 (t, 2H, –C**H**_**2**_–COO–CH_3_), 1.91 (p, 2H, –C**H**_**2**_–). ^13^C NMR (100 MHz, DMSO-*d*_6_): δ (ppm), 174.05, 156.02, 131.42, 120.16, 115.90, 66.95, 30.05, 24.18.

##### Synthesis of (4-azido-phenoxy) Butanoic acid-*N*-hydroxy-succinimide Ester (**5c**)

(4-azido-phenoxy) butanoic acid (**4c**) (97 mg, 0.44 mmol) was dissolved in dichloromethane (1.5 mL). *N*-hydroxy-succinimide (50.5 mg, 0.44 mmol) was added. *N*,*N*′-di-cyclohexyl-carbodiimide (92.3 mg, 0.45 mmol) after dissolved in 0.5 mL dichloromethane, was added dropwise to the above mixture. The mixture was stirred overnight at room temperature, capped, and in the dark. The following day, dicyclohexyl urea produced during the overnight reaction was filtered out from the mixture, and the filtrate was filtered a second time over celite. The solvent of the final filtrate solution was evaporated in vacuo (50 °C) to obtain the desired (4-azido-phenoxy) butanoic acid-*N*-hydroxy-succinimide ester (**5c**) as a red solid (yield: 138 mg, 100%). Exposure to light or keeping the product at room temperature may result in degradation and, therefore, it was stored in the dark below room temperature (<−10 °C) to minimize its decomposition.

^1^H NMR (400 MHz, DMSO-*d*_6_): δ (ppm), 7.07–7.02 (m, 2H, Ph–H), 7.02–6.97 (m, 2H, Ph–H), 4.03 (t, 2H, O–C**H**_**2**_–), 2.84 (t, 2H, –C**H**_**2**_–COO–), 2.81 (s, 4H, C_4_**H**_**4**_NO_2_), 2.06 (p, 2H, –C**H**_**2**_–). ^13^C NMR (100 MHz, DMSO-*d*_6_): δ (ppm), 170.20, 168.73, 155.85, 131.59, 120.16, 115.96, 66.21, 27.04, 25.43, 23.98. IR (ATR diamond crystal) wavenumber (cm^–1^): 825 (s), 889 (s), 944 (w), 1049/1068 (mbr), 1247 (s), 1276 (w), 1378 (w), 1431 (w), 1505 (s), 1196 (s), 1780 (w), 1814 (w), 2114 (s, as stretch N_3_) and 2954 (b). Elemental analysis %: Anal. Calcd for C_14_H_14_N_4_O_5_: C, 52.83; H, 4.43; N, 17.6; O, 25.23. Found: C, 53.86; H, 4.83; N, 16.70; O, 25.43.

#### Adhesion Promoter Synthesis of PAAm-g[4]- X (X = a: DFPxA; b: PFPxA; c: PxA; d: PFPA)

Polyallylamine hydrochloride (PAAm HCl) was used as polymeric backbone for the grafting of the four aryl azide moieties. PAAm repeating units are composed of a methyl amine group (R–CH_2_NH_2_) and two carbon aliphatic spacers. Under mild alkaline environment the reactive azide moieties are grafted to the PAAm backbone with a stoichiometric grafting ratio of 4 (*g* = 4). A solution of poly(allylamine) hydrochloride (14 kDa, PAAm HCl, 3 mg, 0.032 mmol monomer, 4 equivalents) and potassium carbonate (7.53 mg, K_2_CO_3_, 0.054 mmol) was prepared in ultrapure water (598.6 μL) with a magnetic stirrer and briefly heated to boiling. X (a: 2.84 mg; b: 3.13 mg; c: 2.55 mg; d: 2.66 mg, 0.008 mmol) was added to ethanol (970 μL), sonicated for 2–3 min until completely dissolved and then added dropwise to the PAAm solution under vigorous stirring to obtain a theoretical grafting ratio of 4; see Figure 7. The reaction was left to stir overnight, avoiding exposure to ambient light. The polymer solution was diluted to the final desired concentration of 0.1 mg/mL using a 2:1 volume mixture of HEPES 1 buffer (10 mM, adjusted to pH 7.4 with NaOH) to ethanol, where all the amine groups of PAA backbone are in their protonated state (R–NH_3_^+^) to be used directly for coating experiments.

#### Preparation of Homogeneous PAAm-g-X-PVP Coatings (X = a: DFPxA; b: PFPxA; c: PxA; d: PFPA)

Silicon wafers chips cut into 9 × 10 mm rectangles and cleaned by ultrasonication (10 min) twice in toluene and twice in 2-propanol were used for all surface experiments. Immediately before sample preparation, the wafers were exposed to oxygen RF plasma for 2 min. Plasma-cleaned wafers were immersed in the PAAm-g-X adhesion promoter solution for 30 min. Samples were rinsed by exchanging of the PAAm-*g*-X solution twice with HEPES 1/ethanol (2:1), further rinsed in UPW, and blown dry with filtered nitrogen gas. PVP (1300 kDa) dissolved in chloroform (25 mg/mL) was spin-coated (2000 rpm/40 s, 4000 rpm/10 s) onto the PAAm-*g*-X functionalized wafers. To covalently link the polymer with the azides, samples are either temperature (thermolysis) or UV-cured. Two experimental series were performed for temperature curing: first activation at different temperatures for 30 min, second thermolysis at constant temperature (80 °C) for different time periods. To remove excess unbound polymers, the modified surfaces were then rinsed by ultrasonication first in chloroform (5 min) and subsequently in ultrapure water (5 min) followed by immersion overnight in ultrapure water until complete removal of the non-bound excess polymer. Finally, the samples were blow-dried with filtered nitrogen gas.

#### Curing by Thermolysis

Samples were placed on a hot plate for the required time (AREX 6 Digital PRO Hot Plate Stirrer, Velp Scientifica) which has been equilibrated at the corresponding temperature for 20 min. The temperature was measured with a digital humidity and temperature sensor with SDM interface (Sensirion SHT2x).

#### UV-C Curing

Reference samples were illuminated with UV-C light (2 min, 254 nm, at 3.5 mW/cm^2^).

## Supporting Information Available

Additional figures showing the possible decomposition pathways of the aryl azides, DFT calculations and raw data for TGA/DSC, NMR and FTIR-ATR spectra (PDF).
