# Supplementary material for: Development of Low Temperature Activatable Aryl Azide Adhesion Promoters as Versatile Surface Modifiers
Source: ACS Appl Eng Mater. 2025 Apr 4;3(4):867–82. doi: 10.1021/acsaenm.5c00002 (PMC12038790; doi:10.1021/acsaenm.5c00002)
Supplement: Supplementary file 1 — em5c00002_si_001.pdf [file em5c00002_si_001.pdf]

Supporting Information:

Development of low temperature activatable aryl azide adhesion promoters as versatile surface modifiers

Alexandros A. Atzemoglou[a,b], Niccolò Bartalucci[a], Felix Donat[c], Mark W. Tibbitt[b*], Samuele G. P. Tosatti[a], Stefan Zürcher[a*]

[a]SuSoS AG, 8600 Dübendorf, Switzerland

*Email: szuercher.board@susos.com

[b]Department of Mechanical and Process Engineering, Macromolecular Engineering Laboratory, ETH Zurich, 8092 Zurich, Switzerland

*Email: mtibbitt@ethz.ch

[c]Department of Mechanical and Process Engineering, Laboratory of Energy Science and Engineering, ETH Zurich, 8092 Zurich, Switzerland

DIFFERENT AZIDE DECOMPOSITION PATHS

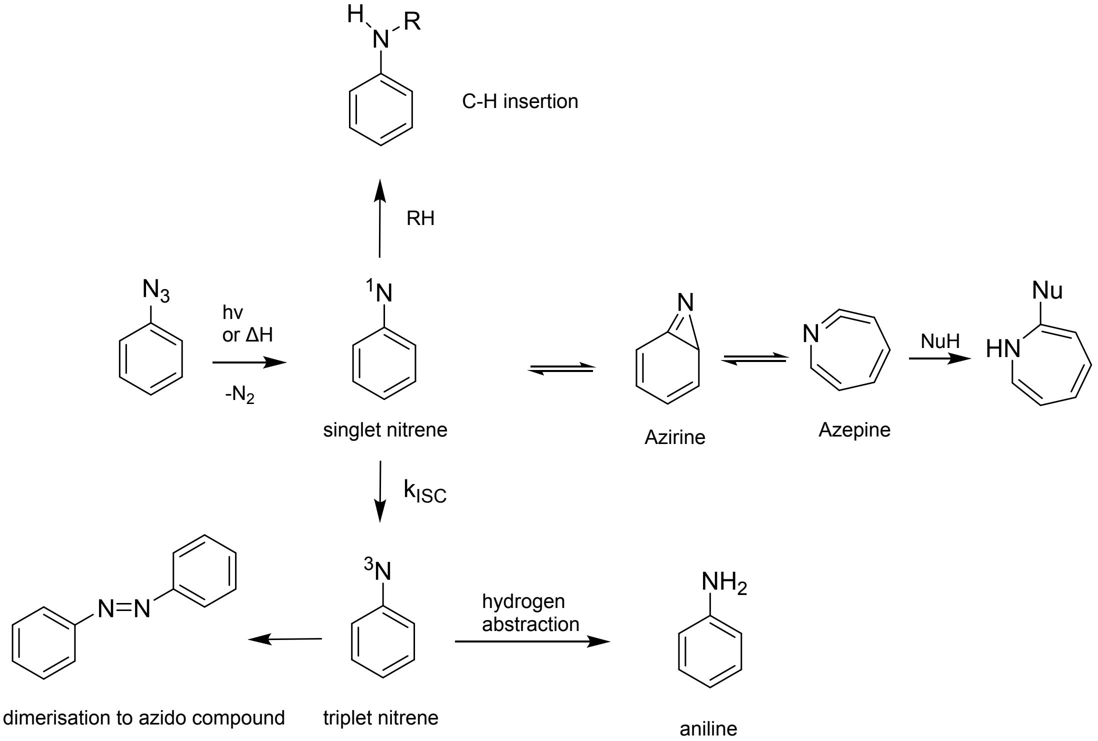

Figure S 1: Possible decomposition reactions of phenyl azide upon thermolysis or photolysis. For use in adhesion promoters, the C-H insertion reaction of the singlet nitrene into a R-H bond of a coating polymer is the preferred path to obtain a covalent binding.[1–3]

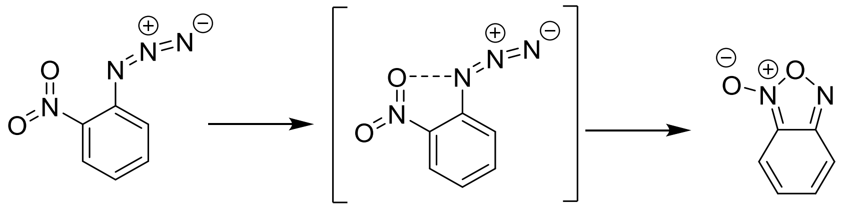

Figure S 2: Decomposition of ortho-nitrophenyl azide to benzofuroxan, through a concerted mechanism and without formation of aryl nitrene.[4,5]

DFT CALCULATIONS OF THE AZIDE ACTIVATION ENERGIES AND ENERGY OF THE FORMED NITRENE OF MONO-SUBSTITUTED PHENYL AZIDE

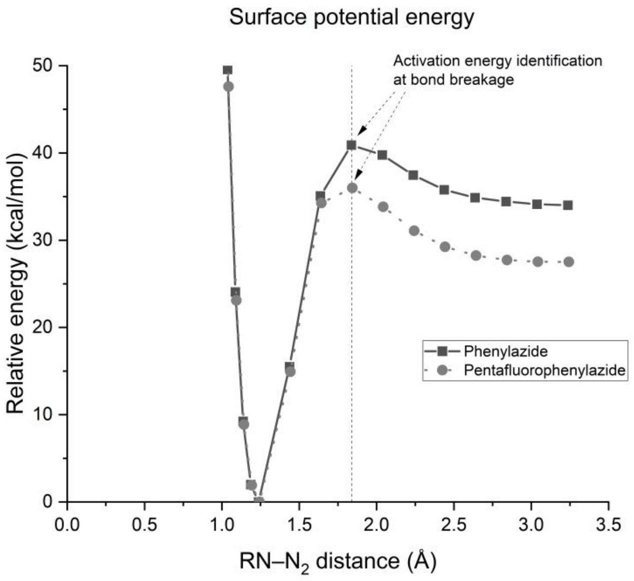

Figure S 3: Calculated surface potential for phenylazide and pentafluoro-phenylazide as function of the ArylN^1–N^{2}N^3 bond distance to determine the activation energy of bond braking to form the corresponding singlet nitrene. R–N_3 → R–N· + N_2. (DFT Theory B3LYP, Basis set 6-31G**).

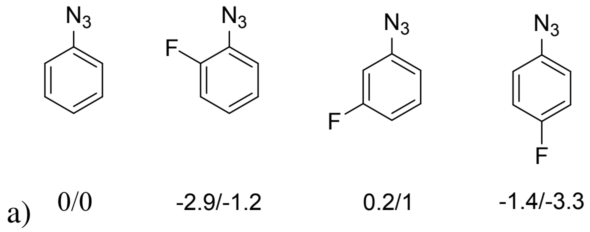

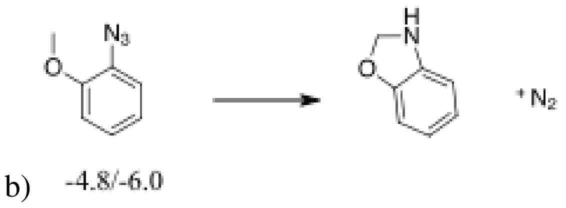

Figure S 4: a) Calculated activation energy and energy of formed singlet nitrene for fluoro-monosubstituted phenylazides in kcal/mol. The values on the left side of the slash symbol represent the ΔE_a and on the right side the energy of the formed nitrene ($\Delta E_{Nitrene}$) relative to the unsubstituted reference compound phenylazide. Negative values mean decrease and positive increase, relative to the reference compound. b) o-Methoxy phenylazide decomposing intramolecularly to form a 5-membered dihydro–oxazole.

KINETIC ANALYSIS ISOTHERMALS FROM TGA MEASUREMENTS

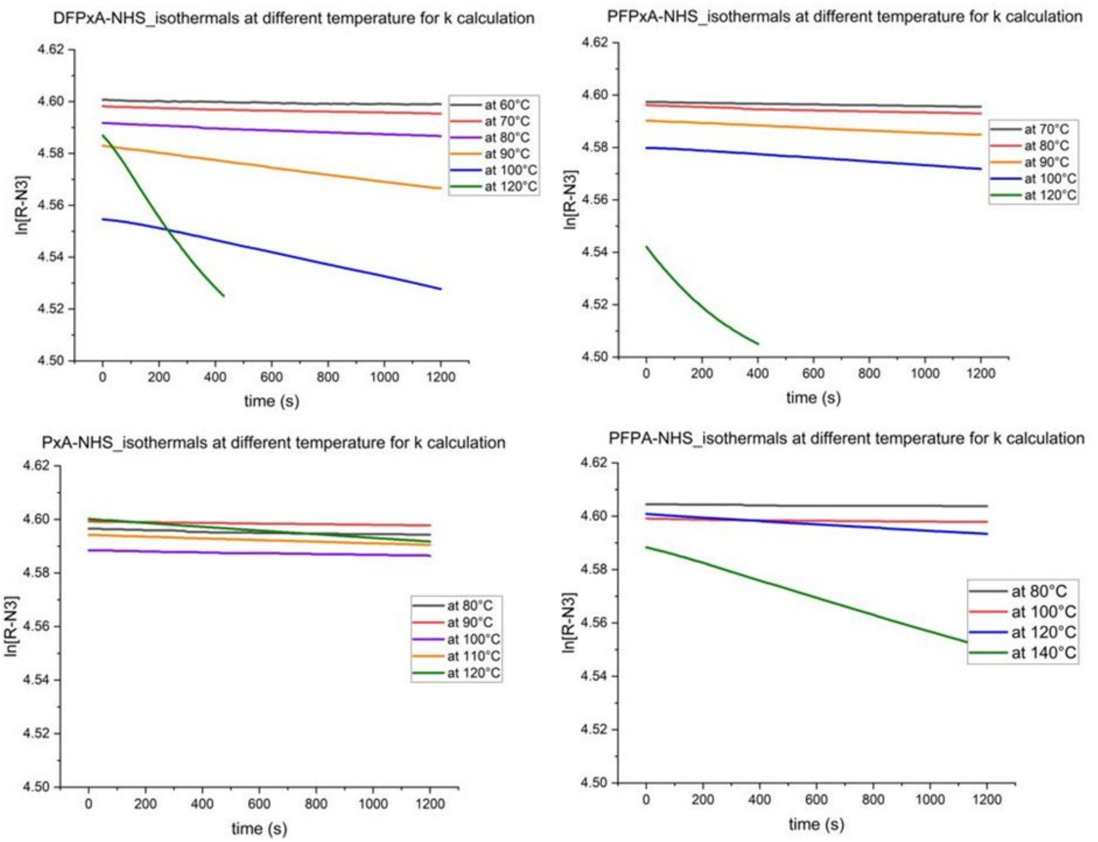

Figure S 5: Linear graph of the natural logarithm of the azide concentration (ln[R–N_3]) over time (t), verifying the first-order reaction kinetics of the azide's decomposition. The reaction rate constant k for a specific temperature is calculated from the slope of the respective curve. Regarding the curve of the isothermal TGA experiment at 120 °C for DFPxA-NHS and PFPxA-NHS, it is plotted for less time since the rate of the azides' consumption is very high and after 400 seconds the curve starts flattening since most of the azides have been decomposed. Note that the colors correspond to different temperatures for the different plots.

Table S 1: Reaction rate constants k(T) calculated for a temperature range from 60-140 °C. The values with the asterisk (*) refer to non-measured values but interpolated from the respective curve of the measured reaction rate constants k over increasing temperature, by the intercept at the desired temperature value (Figure S 5).

Material	Isothermal at 60 °C	Isothermal at 70 °C	Isothermal at 80 °C	Isothermal at 90 °C	Isothermal at 100 °C	Isothermal at 110 °C	Isothermal at 120 °C	Isothermal at 140 °C
					k *(10^{-6}) s^{-1}			
DFPxA-NHS	1.37	2.37	3.39	13.8	23	43.94*	138	-
PFPxA-NHS	-	1.5	1.45	4.6	6.9	19.79*	45.1	-
PxA-NHS	-	-	0.65	1.14	1.6	3.1	12	-
PFPA-NHS	-	-	0.48	-	0.96	2.79*	6.15	31.9

TGA AND DSC CURVES FOR DFPXA-NHS, PFPXA-NHS PXA-NHS AND PFPA-NHS

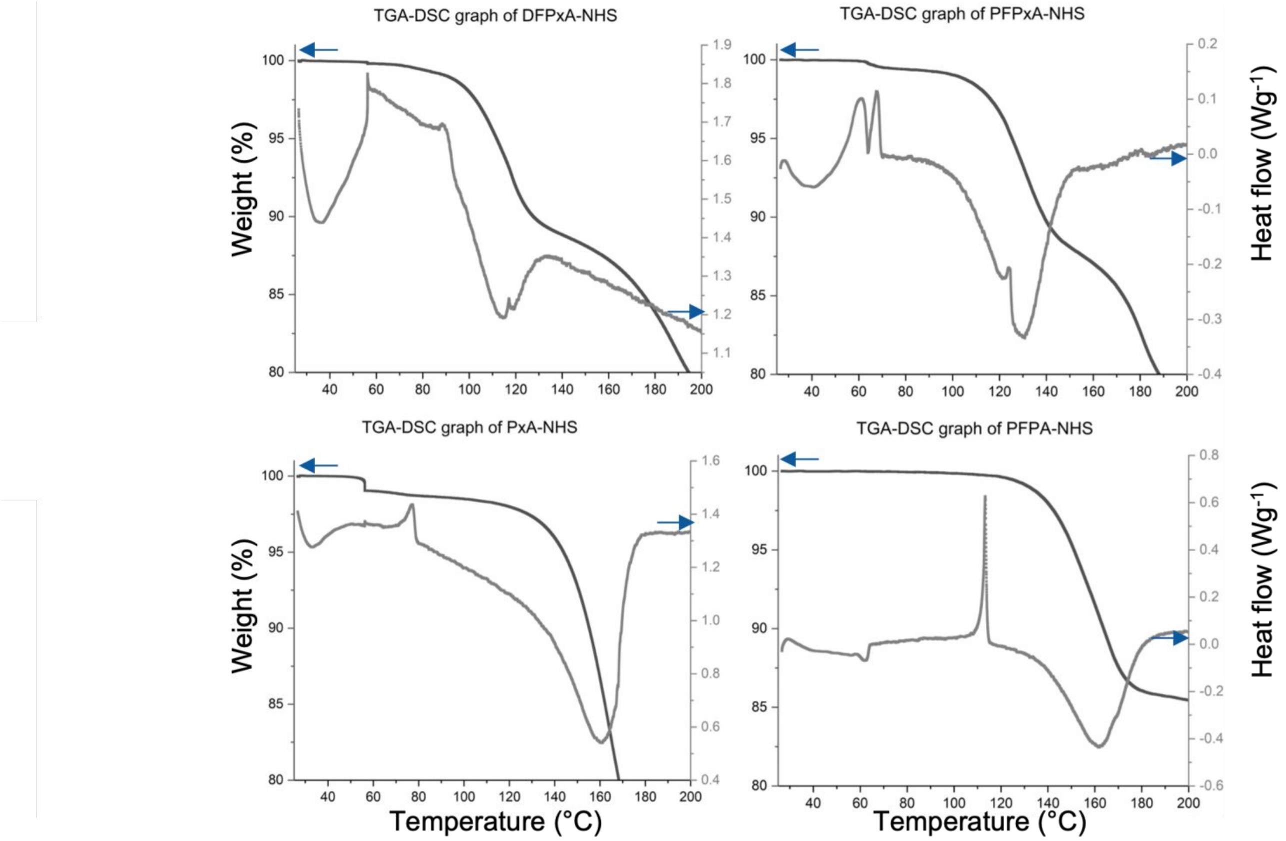

Figure S 6. Double Y-axis graphs of TGA/DSC measurements. Weight% (left) and heat flow (Wg^{-1}) (right) are plotted on the Y-axes versus increasing temperature on the X-axis. The curves indicate the endothermic (ΔH>0) and the exothermic (ΔH<0) incidents taking place throughout the thermal decomposition of the reactive aryl azides in all 4 reactive compounds.

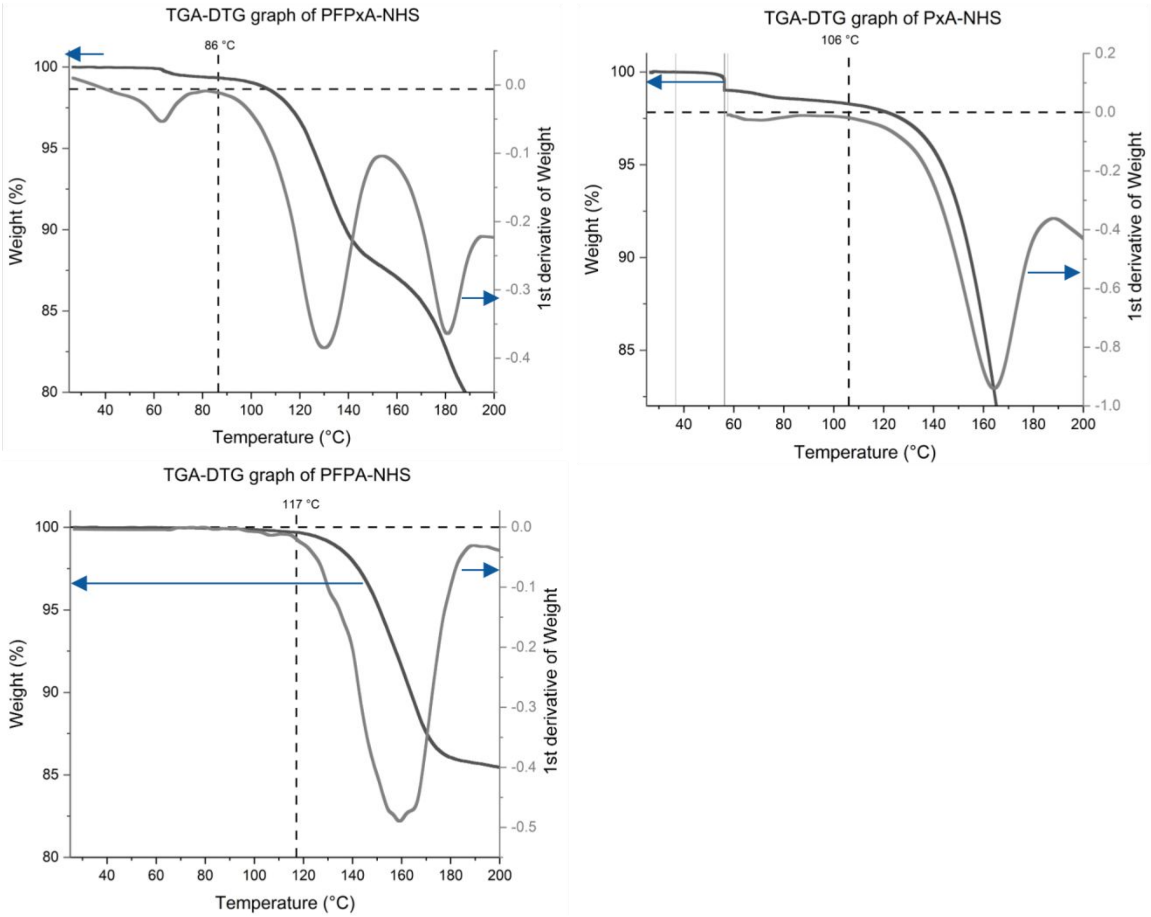

Figure S 7: Double Y-axis graphs of TGA measurements. Weight and 1st derivative of weight are plotted on the Y-axes and increasing temperature on the X-axis. The designated lines point out the initial reaction point temperature of azide decomposition in the remaining 3 reactive compounds, where the 1st derivative value deviates from the equilibrium. Each compound contained different amounts of entrapped solvents which is depicted in the 1st derivative curves but should not be confused with the azide decomposition. For that reason, it is crucial to understand each change, by careful analysis and comparison of both the TGA-DSC and TGA-DTG data. Therefore, a different baseline was used for each compound to determine the initial decomposition point.

LN(K) OVER 1/T CURVES FROM KINETIC ANALYSIS

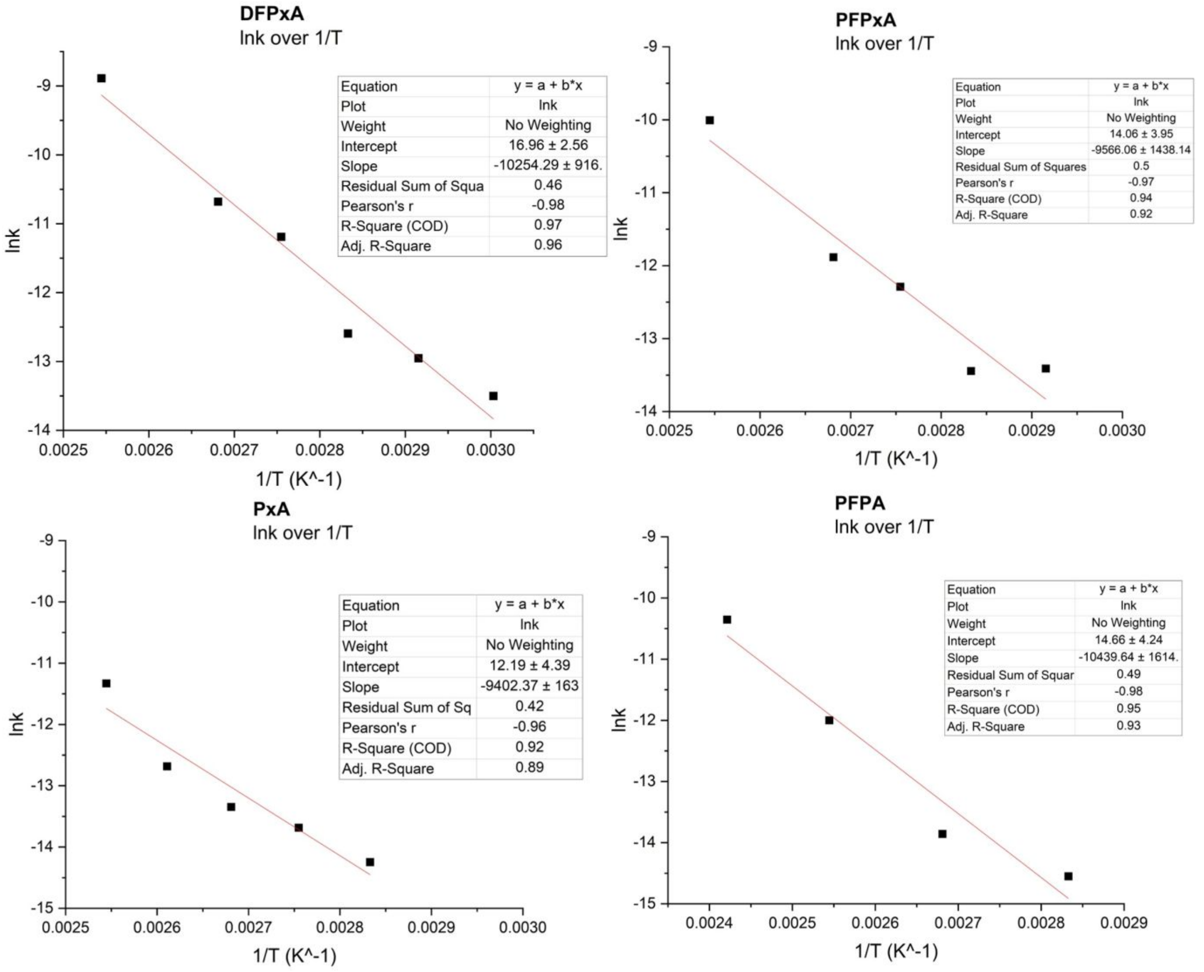

Figure S 8. Graphs of the experimental TGA data where the natural logarithm of the reaction rate constant k is plotted over the inverse temperature. The lines were used to interpolate the values of the k at some temperatures for the different reactive compounds.

NMR DATA

1D ^{1}H, ^{19}F and ^{13}C NMR spectra for synthesis of (4-azido-3,5-difluorophenoxy) butanoic acid – N-hydroxysuccinimide ester

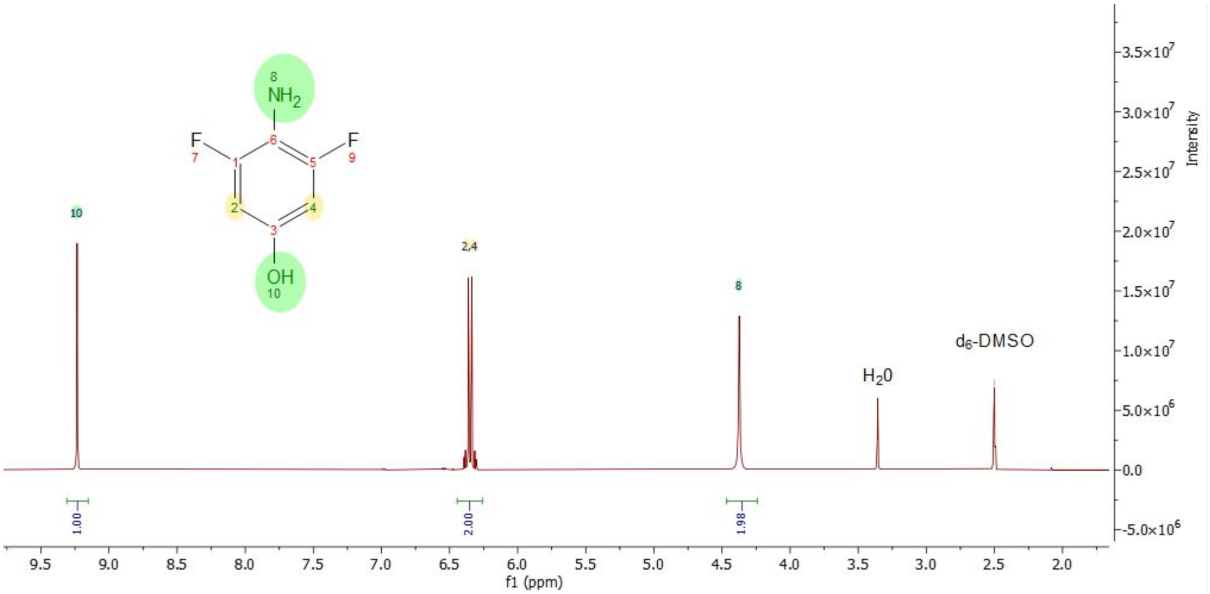

Figure S 9: ^{1}H NMR spectrum of 4-amino-3,5-difluorophenol **1a** (400MHz, DMSO-d_6). Measurement as received.

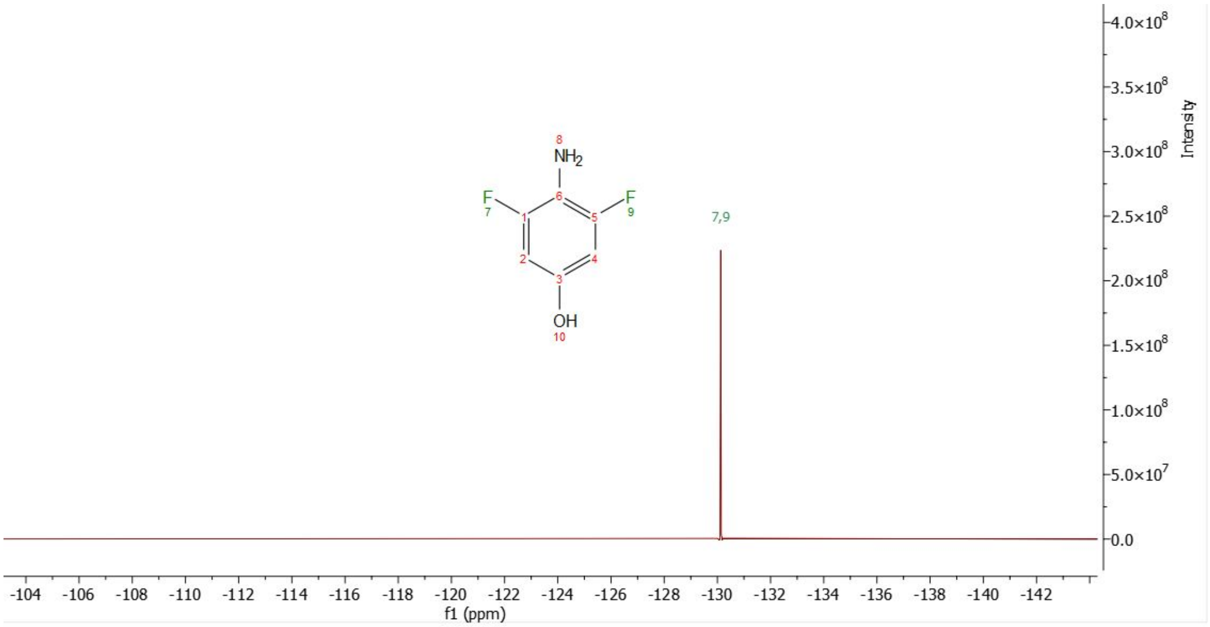

Figure S 10: ^{19}F NMR spectrum of 4-amino-3,5-difluorophenol **1a** (400MHz, DMSO-d_6). Measurement as received.

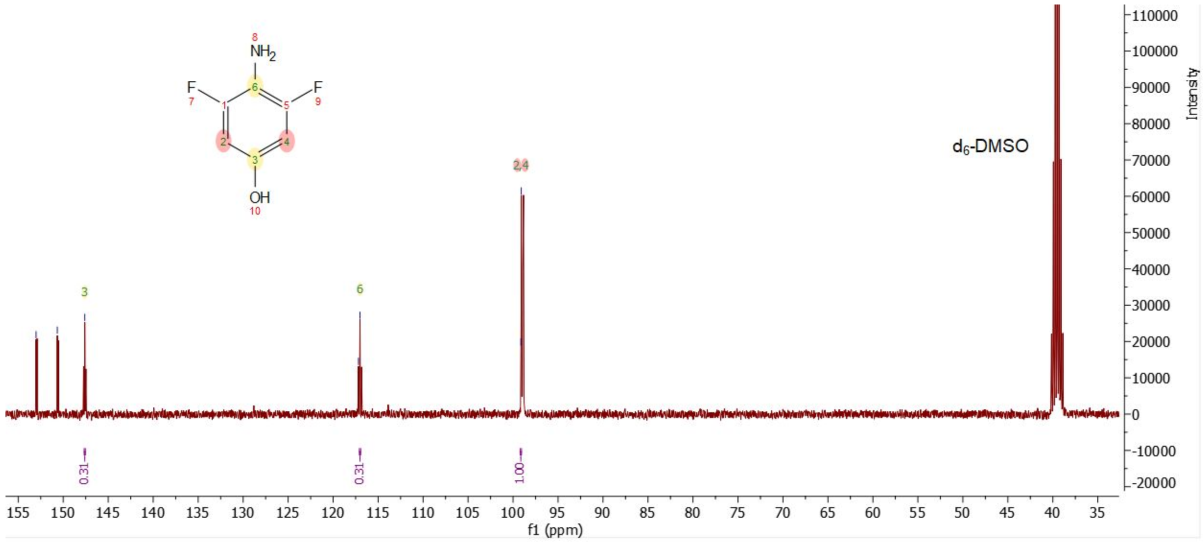

Figure S 11: ^{13}C NMR spectrum of 4-amino-3,5-difluorophenol **1a** (400MHz, DMSO-d_6). Measurement as received.

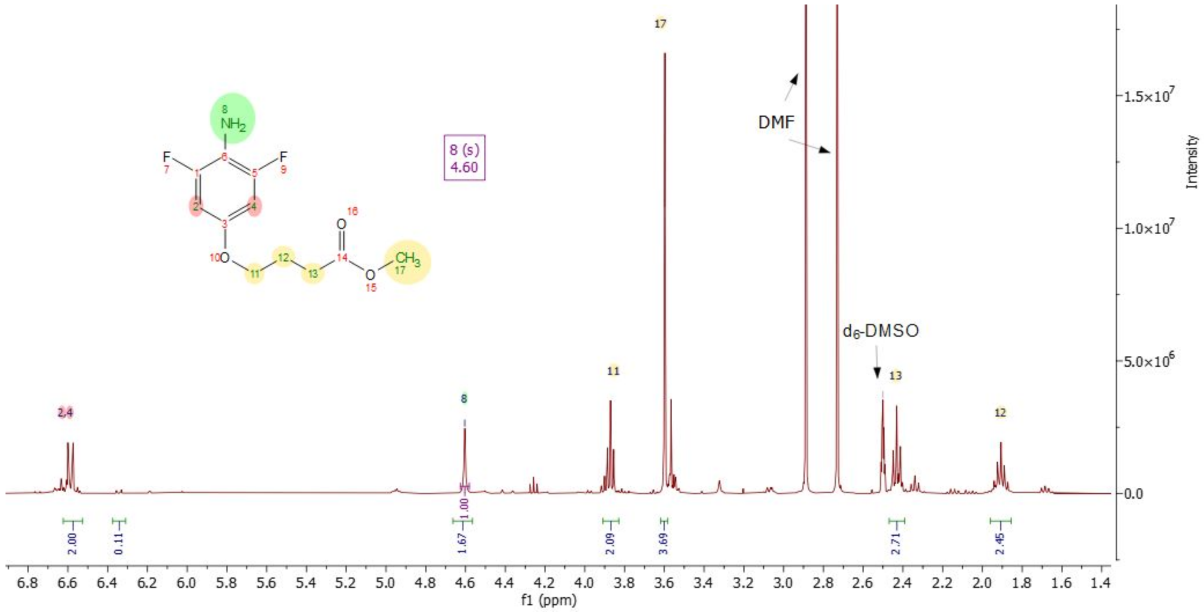

Figure S 12: ^{1}H NMR spectrum of crude methyl-4-(4-amino-3,5-difluorophenoxy) butanoate **2a** (400MHz, DMSO-d_6). ~95% functionalization of –OH of starting material, calculated by the presence of the residual aromatic peaks of the starting material after reaction.

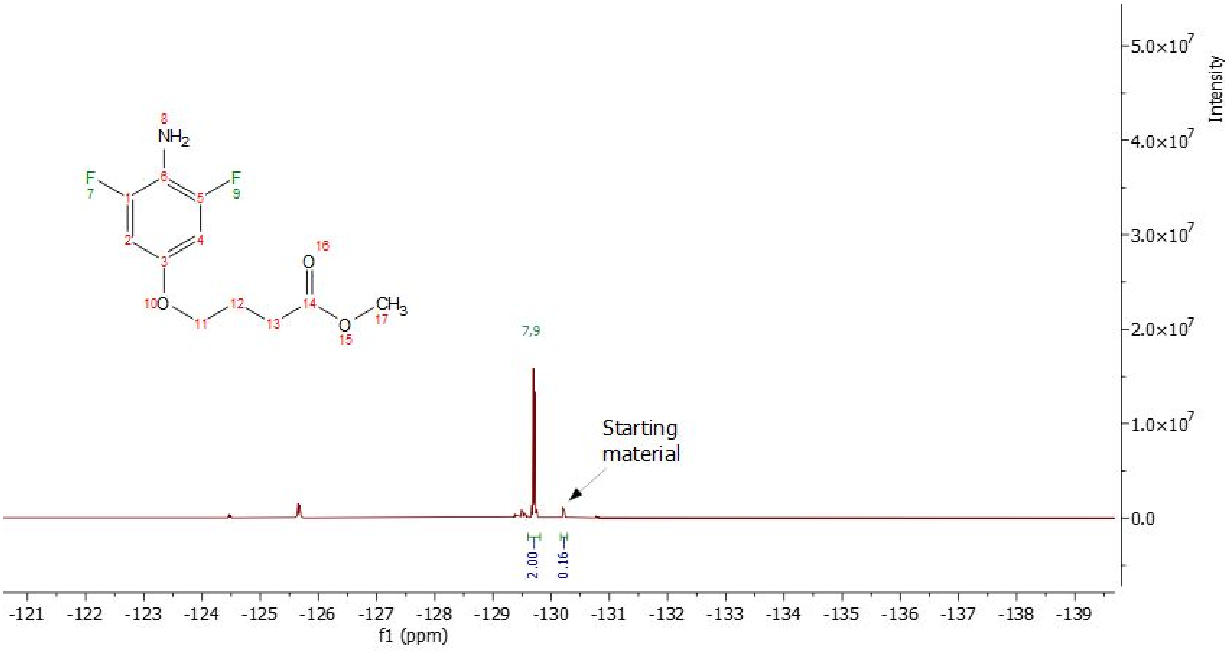

Figure S 13: ^{19}F NMR spectrum of crude methyl-4-(4-amino-3,5-difluorophenoxy) butanoate **2a** (400MHz, DMSO-d$_6$).

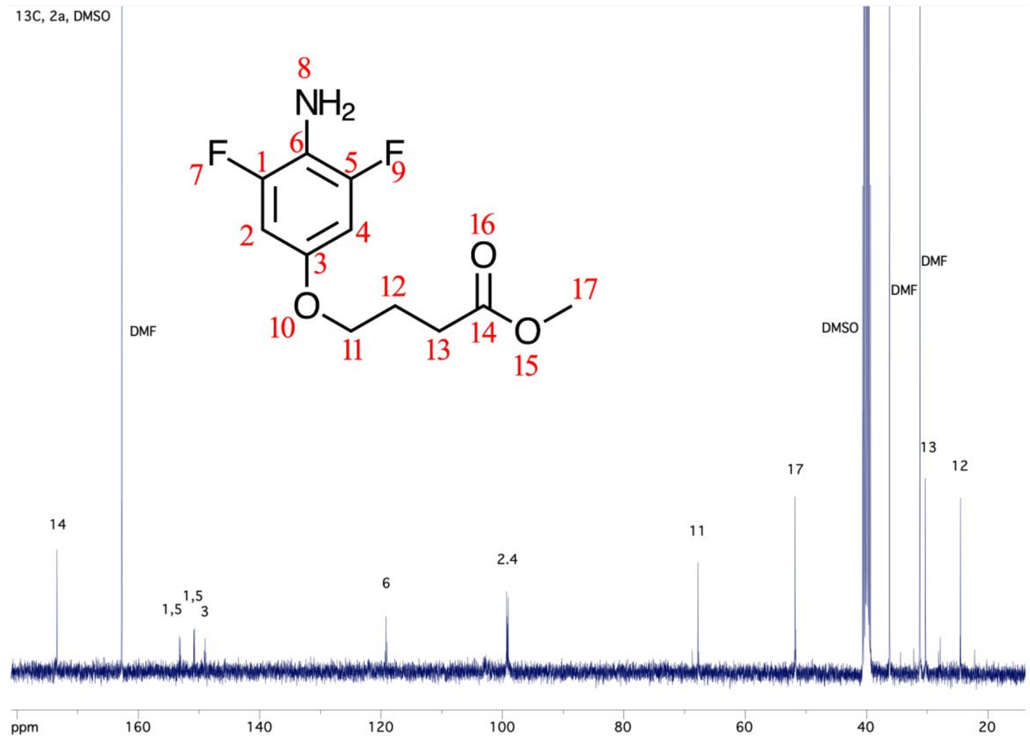

Figure S 14: ^{13}C NMR spectrum of crude methyl-4-(4-amino-3,5-difluorophenoxy) butanoate **2a** (400MHz, DMSO-d$_6$).

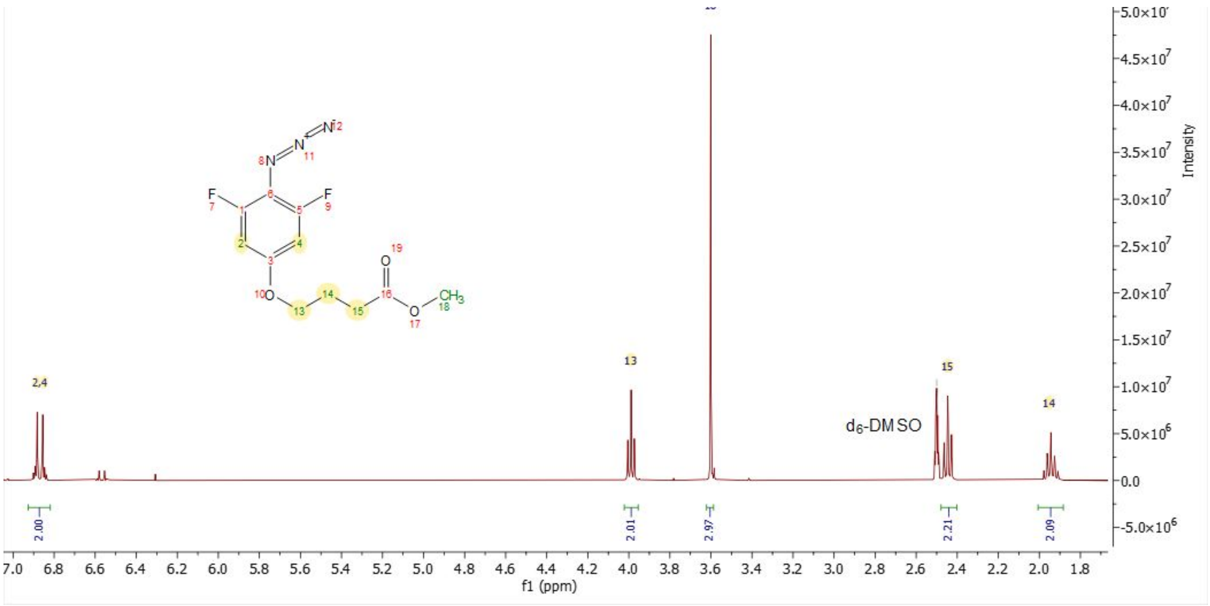

Figure S 15: ^{1}H NMR spectrum of methyl-4-(4-azido-3,5-difluorophenoxy) butanoate **3a** (400MHz, DMSO-d_6). After column purification.

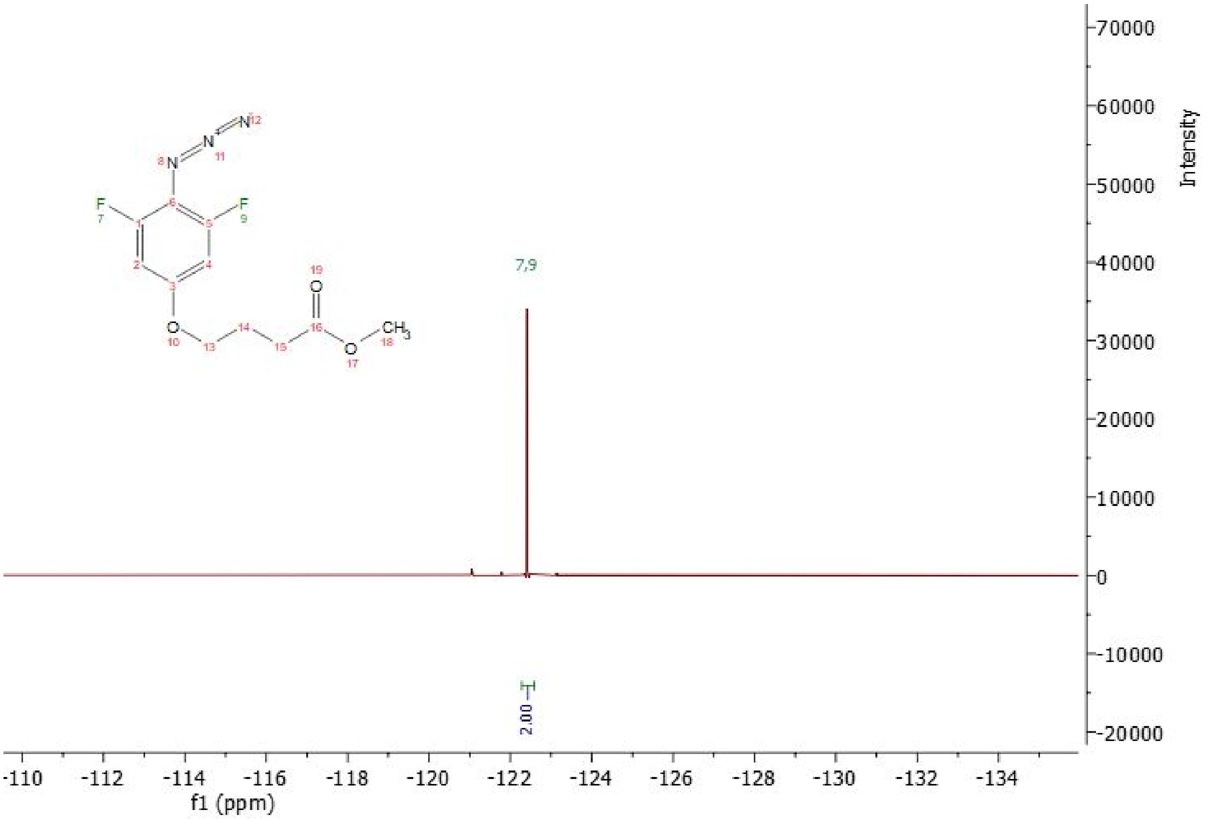

Figure S 16: ^{19}F NMR spectrum of methyl-4-(4-azido-3,5-difluorophenoxy) butanoate **3a** (400MHz, DMSO-d_6). After column purification.

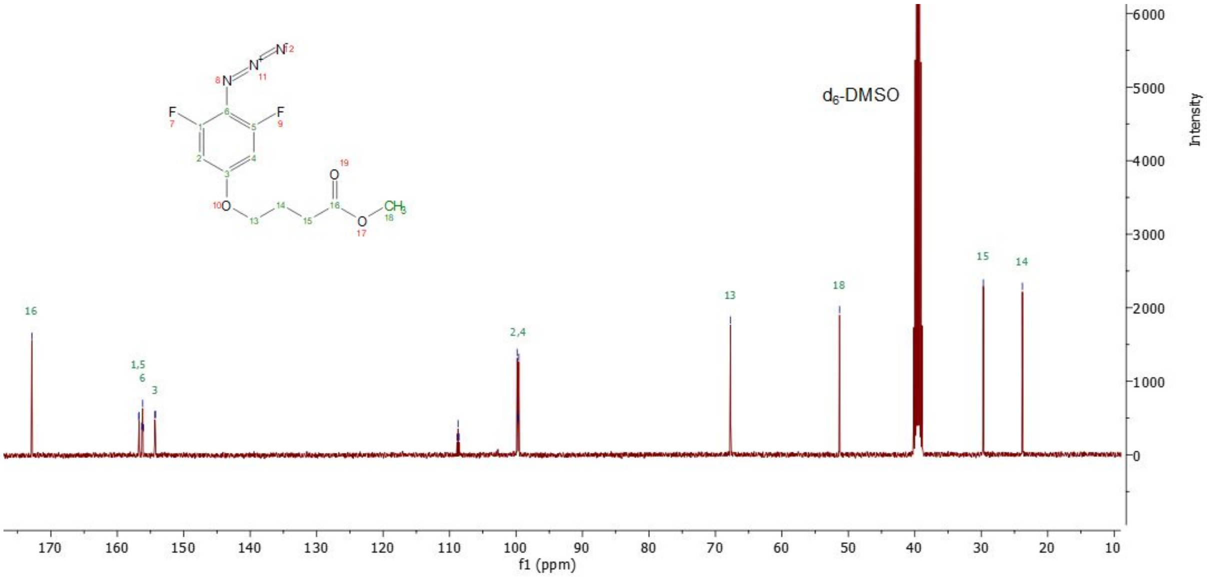

Figure S 17: ^{13}C NMR spectrum of methyl-4-(4-azido-3,5-difluorophenoxy) butanoate **3a** (400MHz, DMSO-d_6). After column purification.

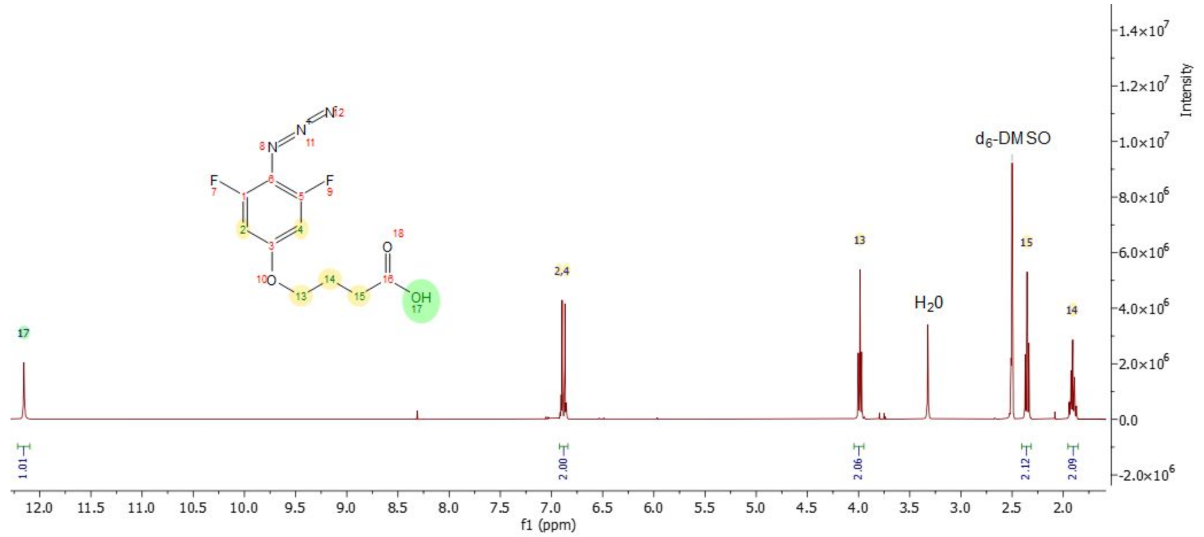

Figure S 18: ^{1}H NMR spectrum of (4-azido-3,5-difluorophenoxy) butanoic acid **4a** (400MHz, DMSO-d_6). Ester hydrolyzed completely.

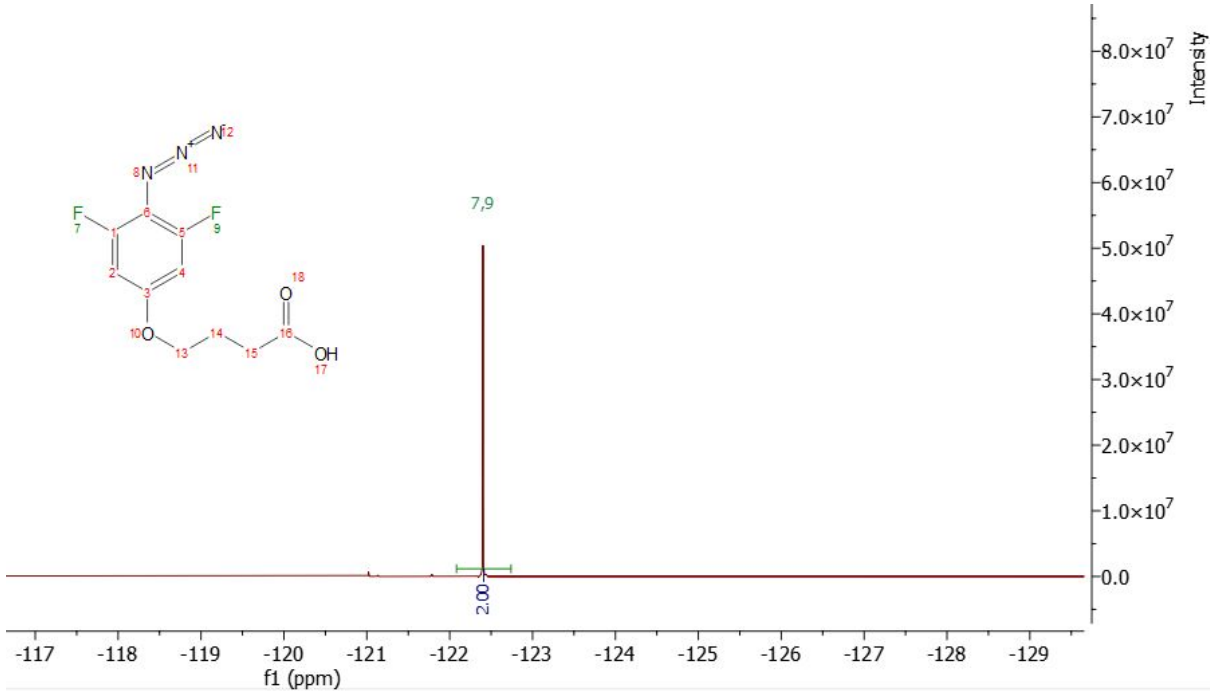

Figure S 19: ^{19}F NMR spectrum of (4-azido-3,5-difluorophenoxy) butanoic acid **4a** (400MHz, DMSO-d_6).

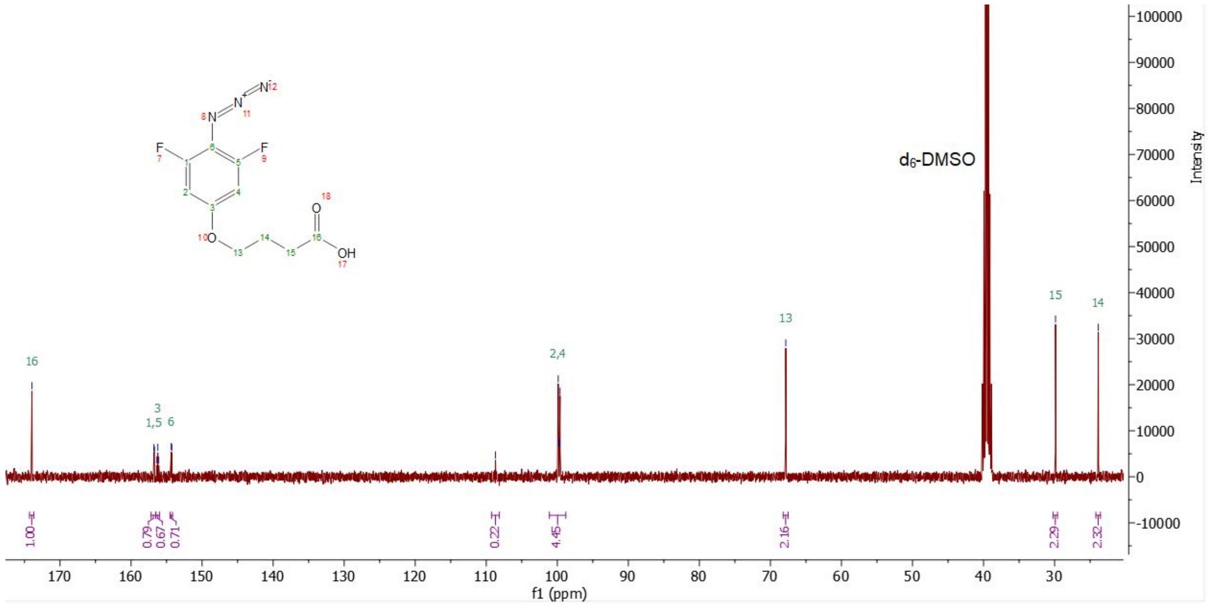

Figure S 20: ^{13}C NMR spectrum of (4-azido-3,5-difluorophenoxy) butanoic acid **4a** (400MHz, DMSO-d_6).

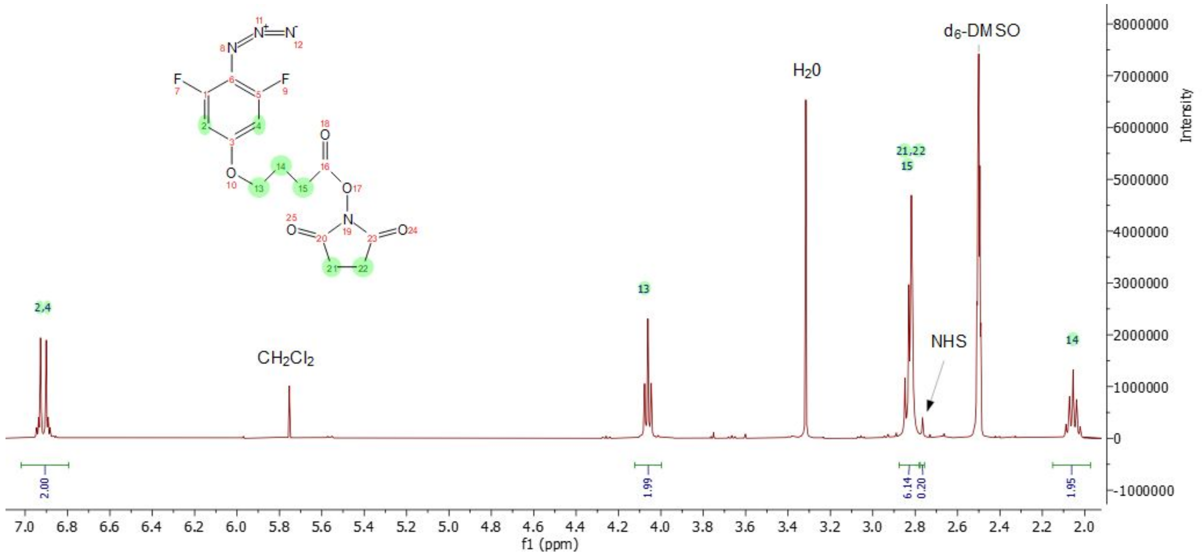

Figure S 21: ^{1}H NMR spectrum of (4-azido-3,5-difluorophenoxy) butanoic acid – N-hydroxysuccinimide ester **5a** (400MHz, DMSO-d_6). NHS ester formation 96% and 96.7% purity calculated by ^{1}H NMR.

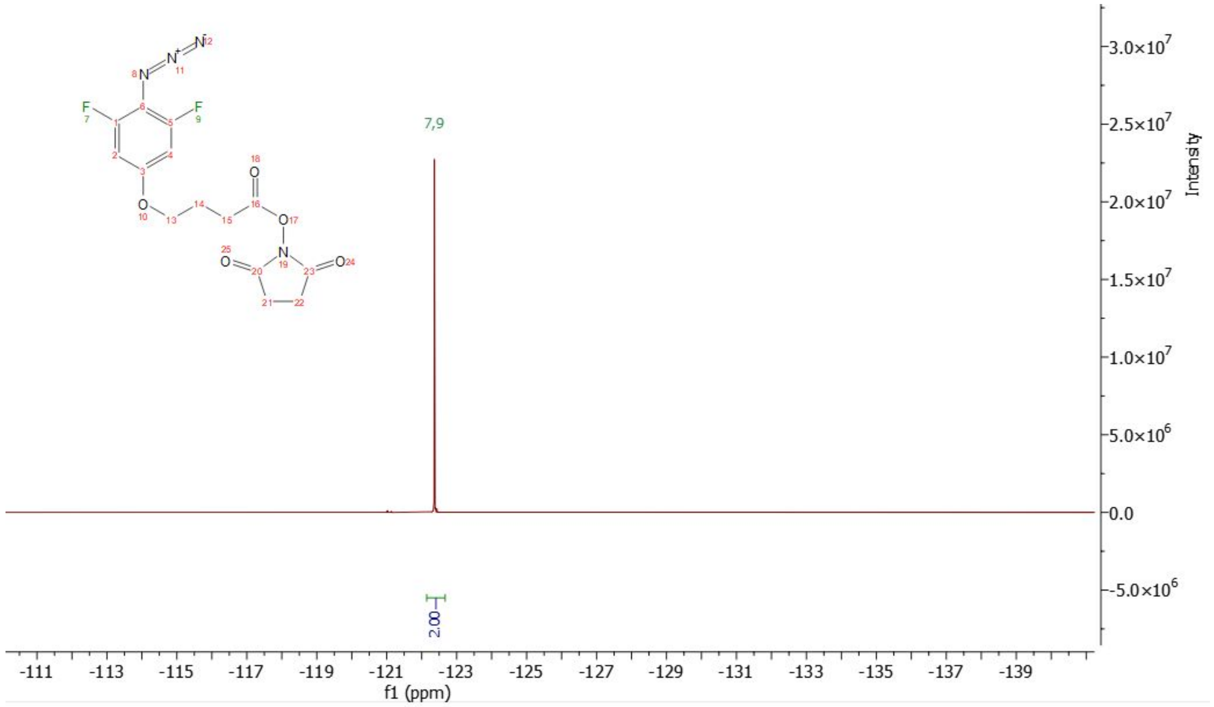

Figure S 22: ^{19}F NMR spectrum of (4-azido-3,5-difluorophenoxy) butanoic acid – N-hydroxysuccinimide ester **5a** (400MHz, DMSO-d_6).

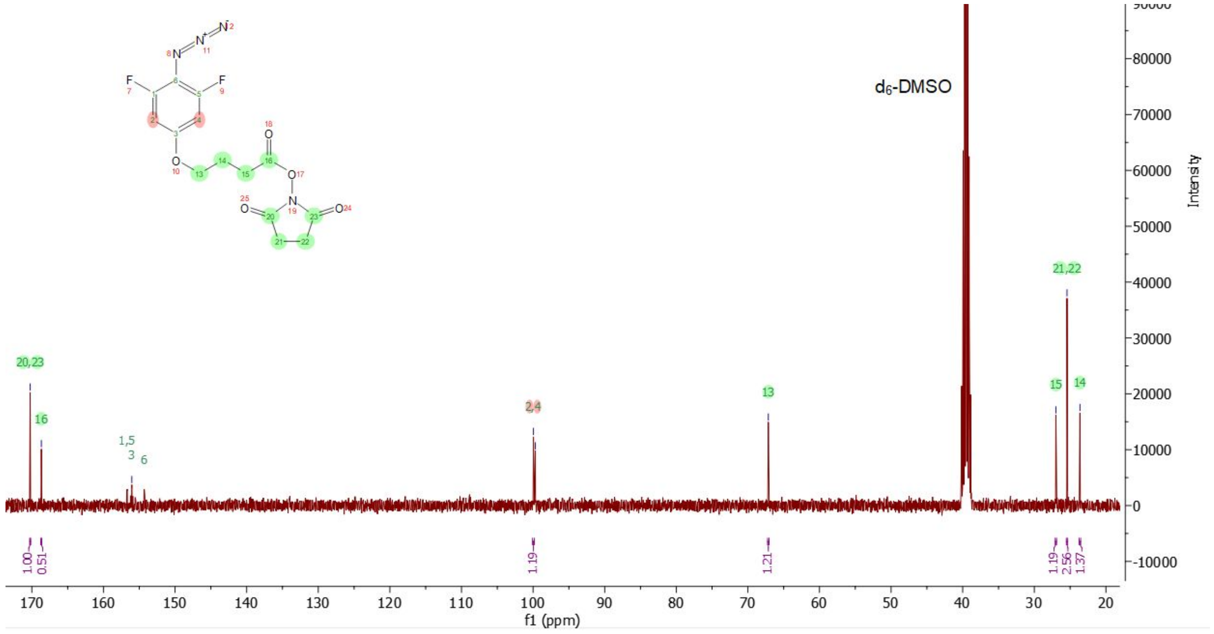

Figure S 23: ^{13}C NMR spectrum of (4-azido-3,5-difluorophenoxy) butanoic acid – N-hydroxysuccinimide ester **5a** (400MHz, DMSO-d_6).

1D ^{1}H, ^{19}F and ^{13}C NMR NMR spectra for synthesis of (4-azido-2,3,5,6-tetrafluorophenoxy) butanoic acid – N-hydroxysuccinimide ester

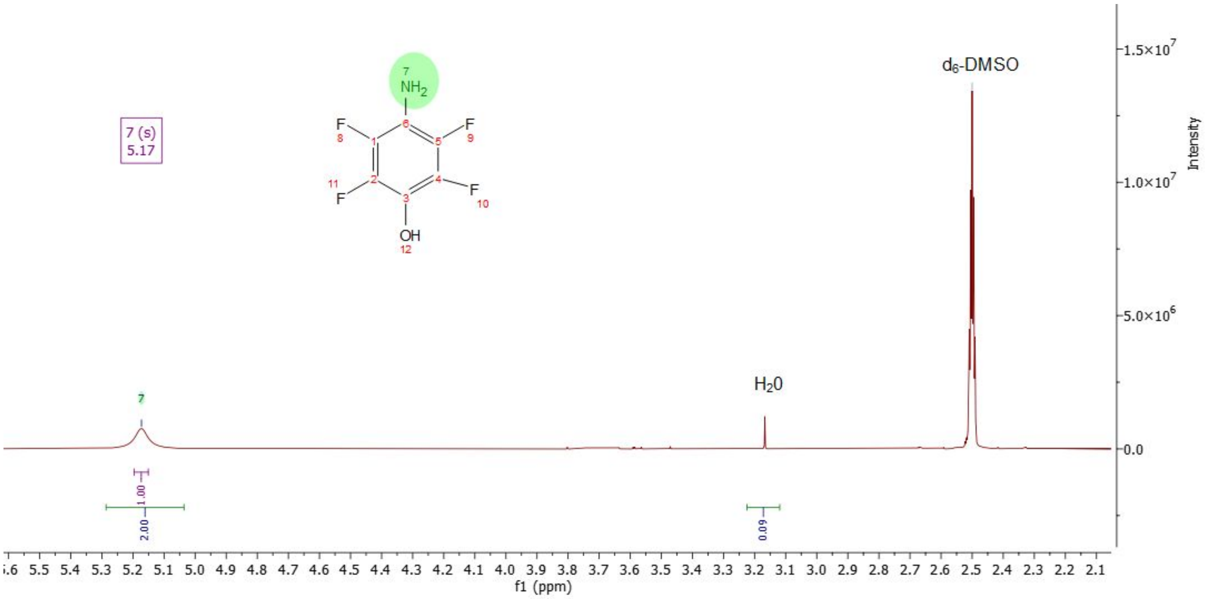

Figure S 24: ^{1}H NMR spectrum of 4-amino-2,3,5,6-tetrafluorophenol **1b** (400MHz, DMSO-d_6). Measurement as received.

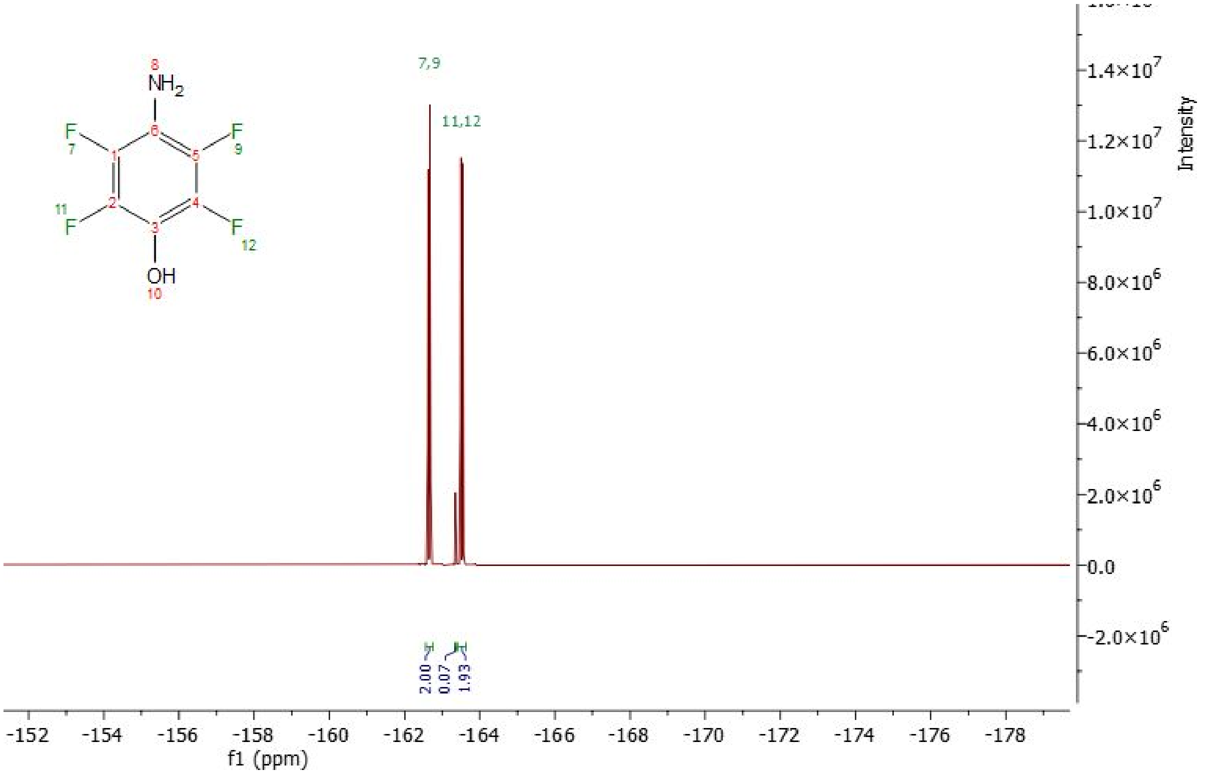

Figure S 25: ^{19}F NMR spectrum of 4-amino-2,3,5,6-tetrafluorophenol **1b** (400MHz, DMSO-d_6). Measurement as received.

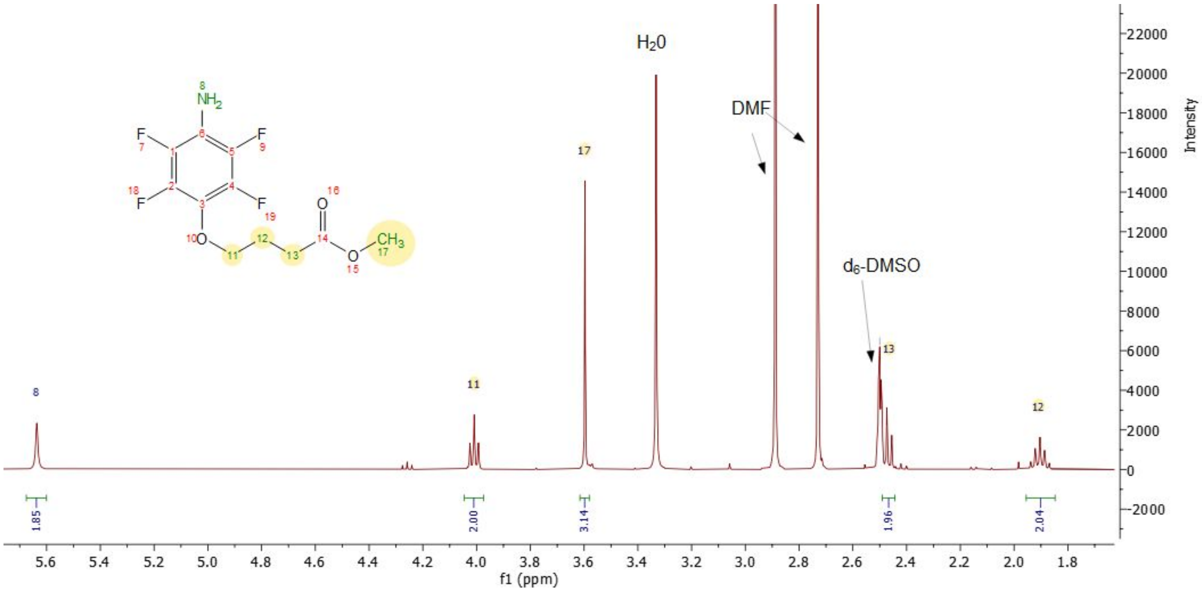

Figure S 26: ^1H NMR spectrum of methyl-4-(4-amino-2,3,5,6-tetrafluorophenoxy) butanoate **2b** (400MHz, DMSO-d_6). Complete functionalization of –OH of starting material determined by ^1H-NMR and the absence of any residual peaks of the starting material after reaction.

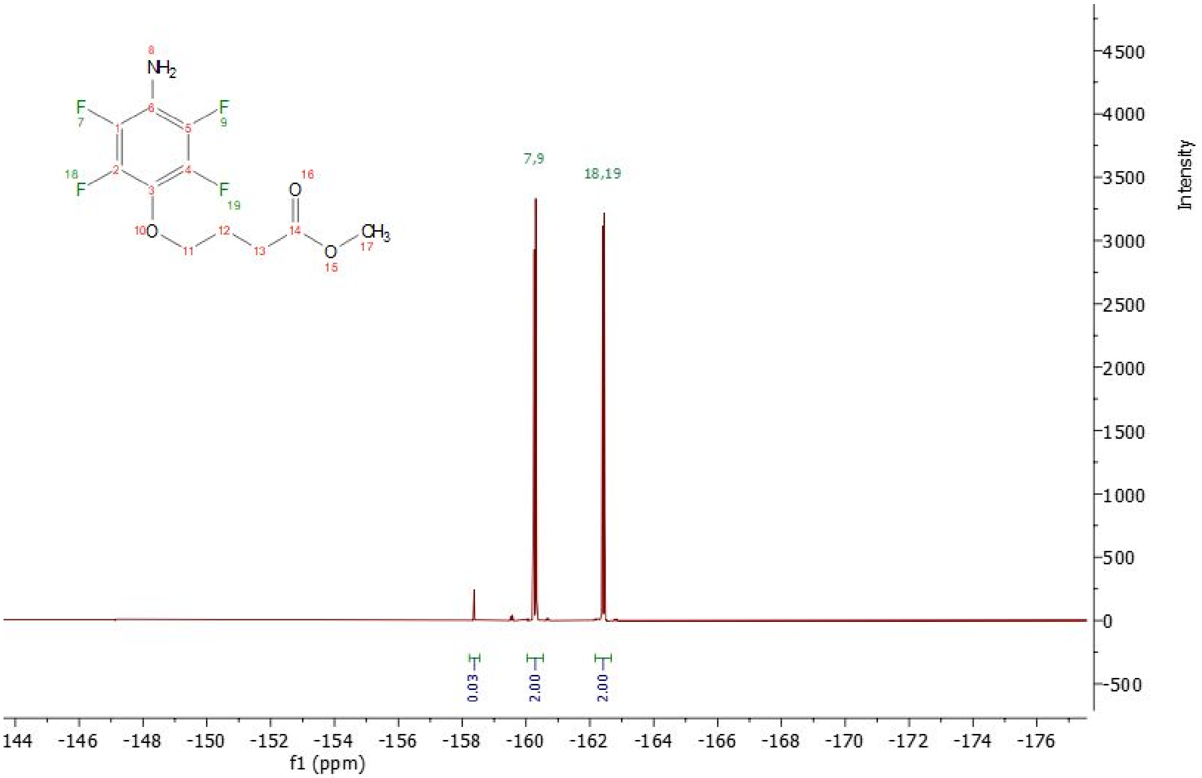

Figure S 27: ^{19}F NMR spectrum of methyl-4-(4-amino-2,3,5,6-tetrafluorophenoxy) butanoate **2b** (400MHz, DMSO-d_6).

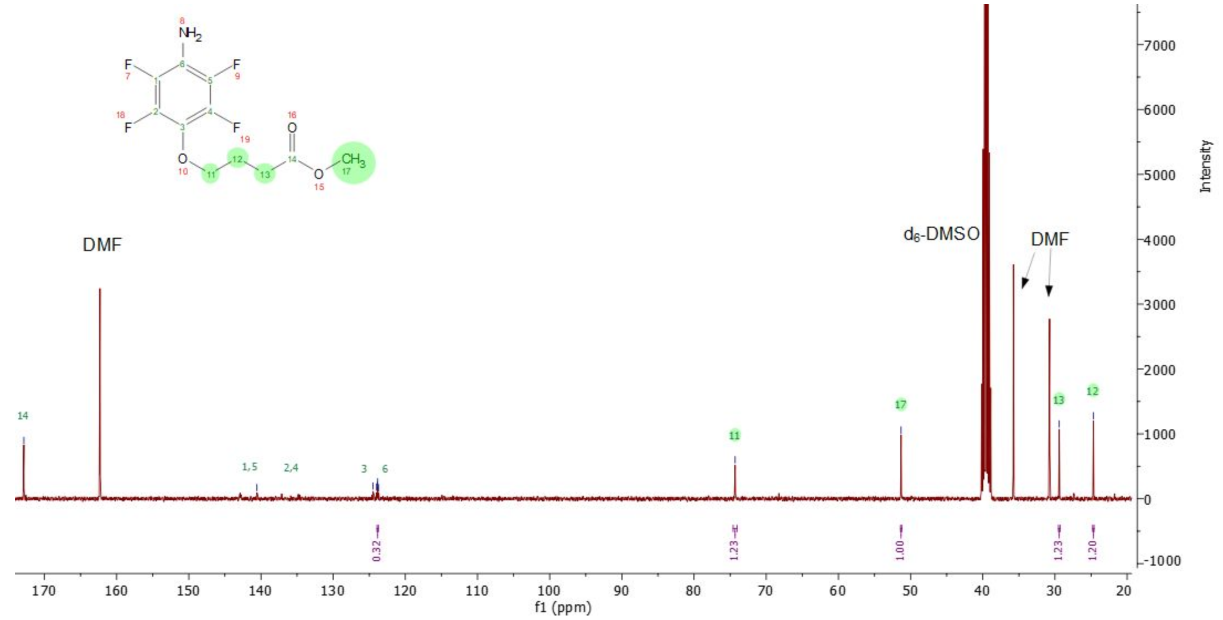

Figure S 28: ^{13}C NMR spectrum of methyl-4-(4-amino-2,3,5,6-tetrafluorophenoxy) butanoate **2b** (400MHz, DMSO-d_6).

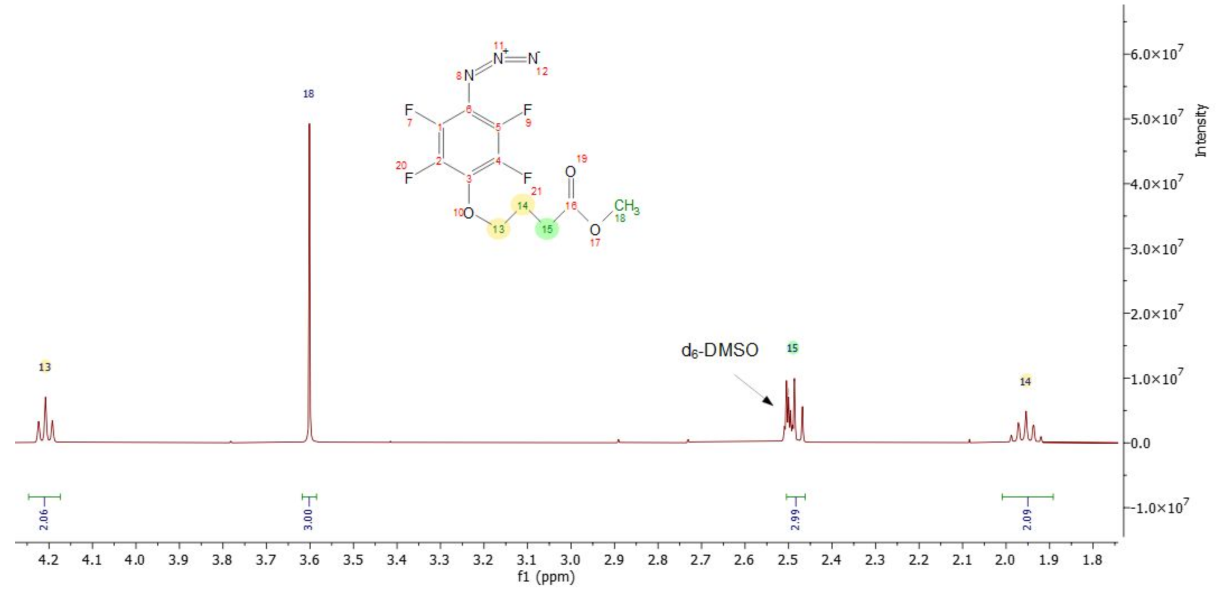

Figure S 29: ^{1}H NMR spectrum of methyl-4-(4-azido-2,3,5,6-tetrafluorophenoxy) butanoate **3b** (400MHz, DMSO-d_6). After column purification.

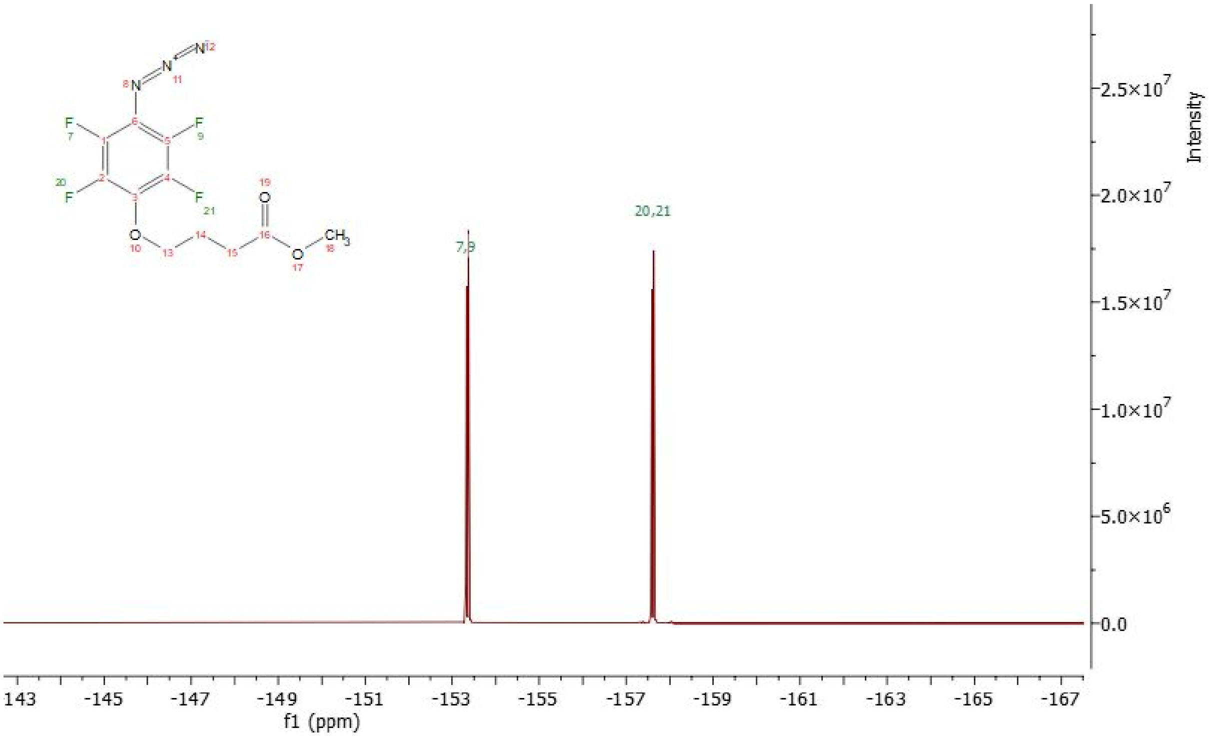

Figure S 30: ^{19}F NMR spectrum of methyl-4-(4-azido-2,3,5,6-tetrafluorophenoxy) butanoate **3b** (400MHz, DMSO-d_6). After column purification.

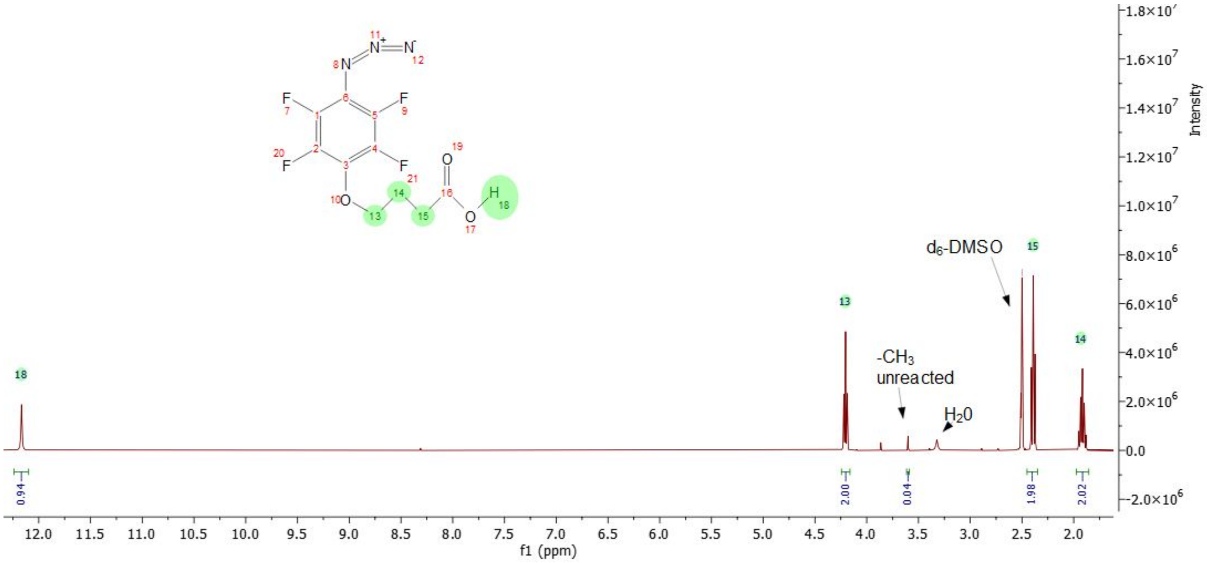

Figure S 31: ^{1}H NMR spectrum of (4-azido-2,3,5,6-tetrafluorophenoxy) butanoic acid **4b** (400MHz, DMSO-d_6). Ester hydrolysis ~98%, calculated by ^{1}H NMR and the residual –CH_3 peak.

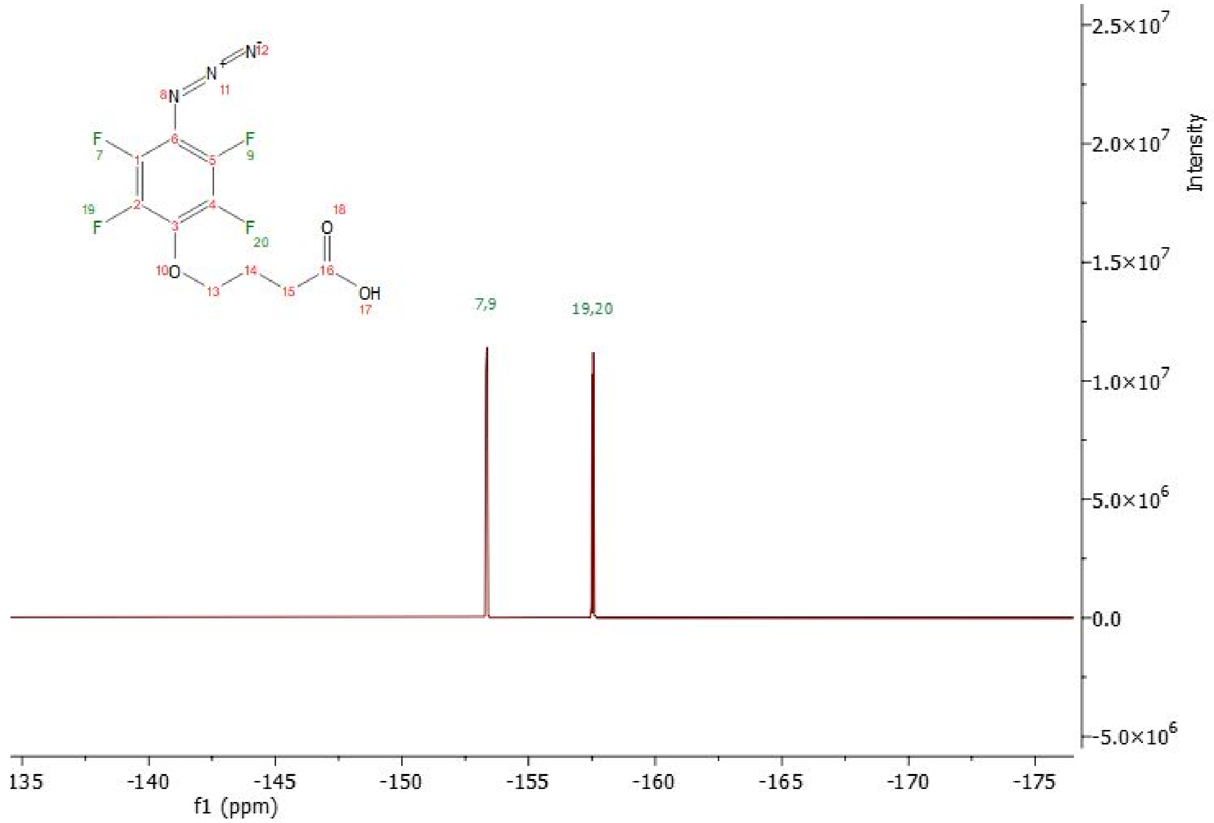

Figure S 32: ^{19}F NMR spectrum of (4-azido-2,3,5,6-tetrafluorophenoxy) butanoic acid **4b** (400MHz, DMSO-d_6).

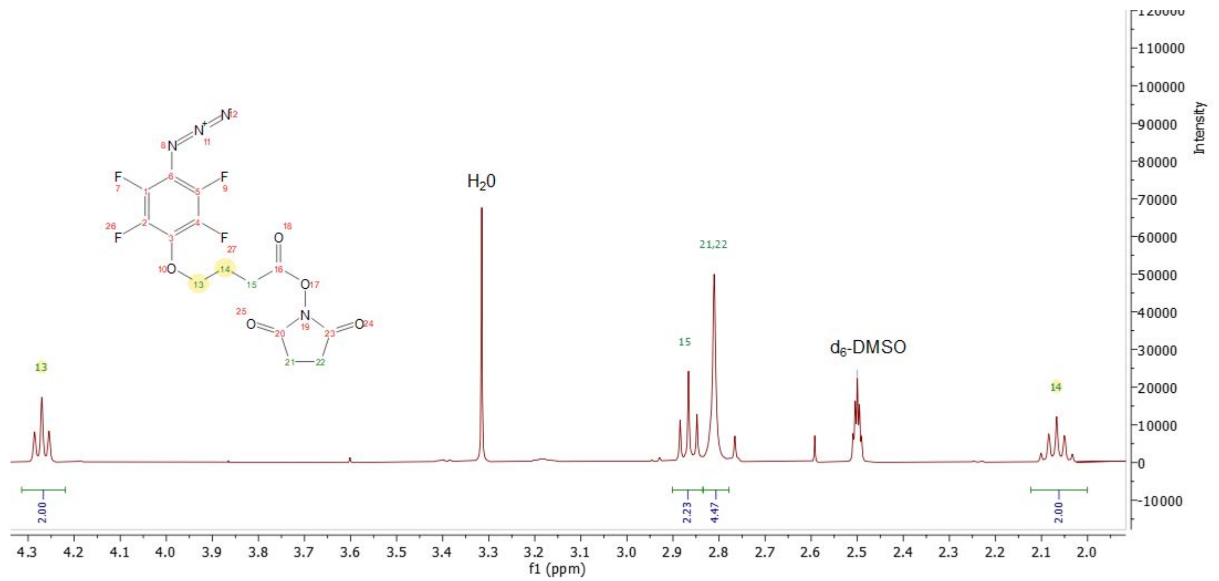

Figure S 33: ^{1}H NMR spectrum of (4-azido-2,3,5,6-tetrafluorophenoxy) butanoic acid – N-hydroxysuccinimide ester **5b** (400MHz, DMSO-d_6). NHS ester formation 96% and 90% purity calculated by ^{1}H NMR.

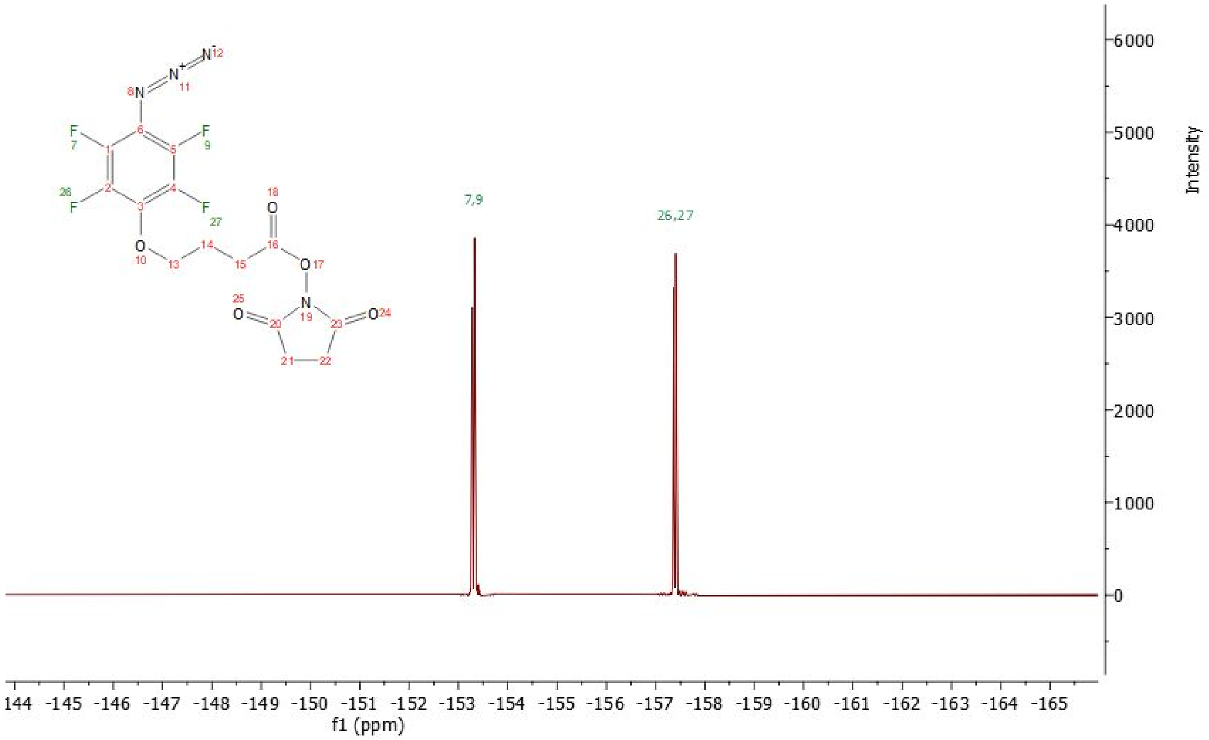

Figure S 34: ^{19}F NMR spectrum of (4-azido-2,3,5,6-tetrafluorophenoxy) butanoic acid – N-hydroxysuccinimide ester **5b** (400MHz, DMSO-d_6).

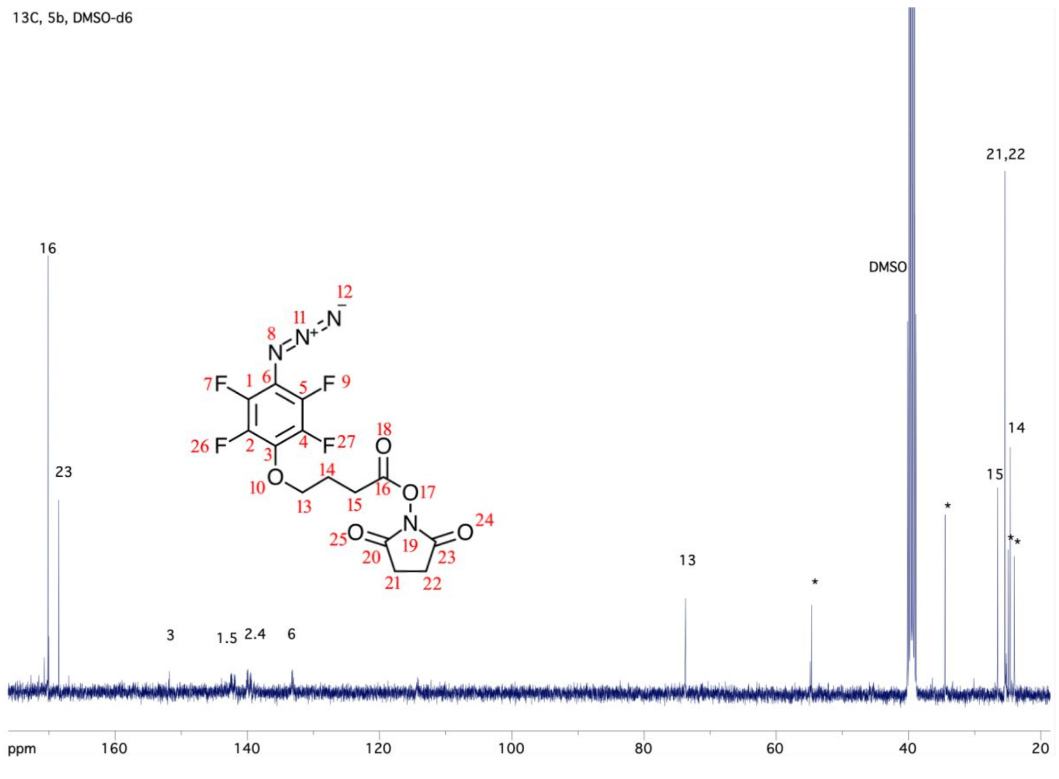

Figure S 35: ^{13}C NMR spectrum of (4-azido-2,3,5,6-tetrafluorophenoxy) butanoic acid – N-hydroxysuccinimide ester **5b** (400MHz, DMSO-d_6). Peaks labeled with * from residual dicyclohexylurea.

1D ^{1}H and ^{13}C NMR NMR spectra for synthesis of (4-azido-phenoxy) butanoic acid – N-hydroxysuccinimide ester

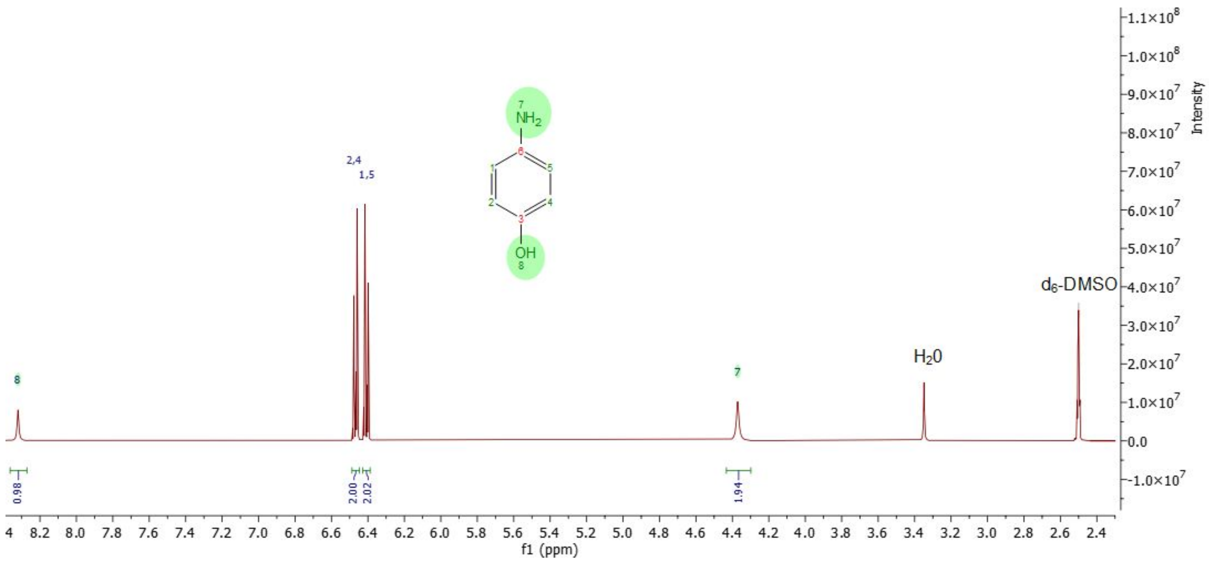

Figure S 36: ^{1}H NMR spectrum of 4-amino-phenol **1c** (400MHz, DMSO-d_6). Measurement as received.

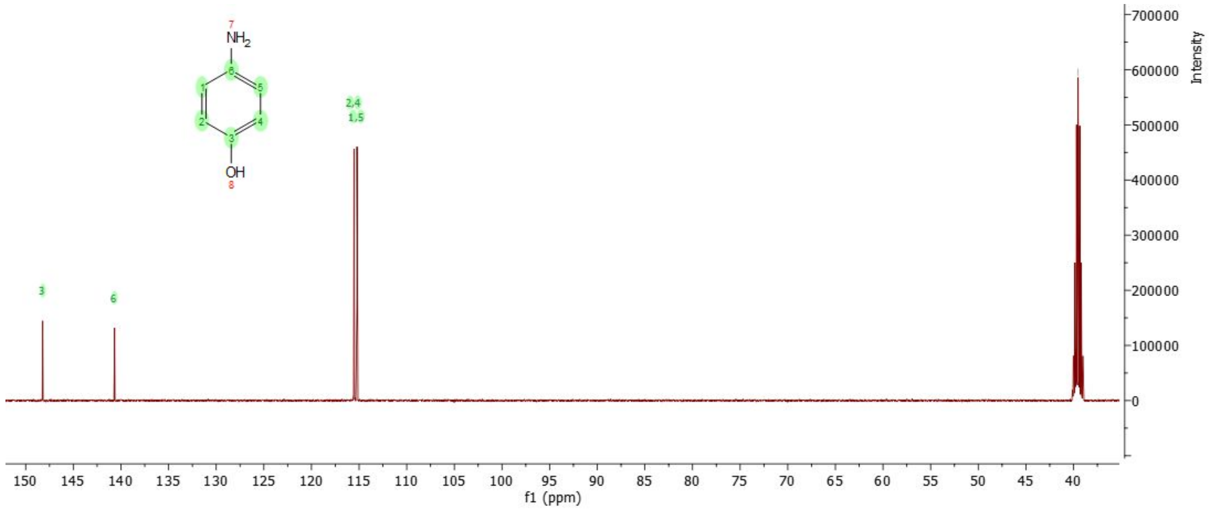

Figure S 37: ^{13}C NMR spectrum of 4-amino-phenol **1c** (400MHz, DMSO-d_6). Measurement as received.

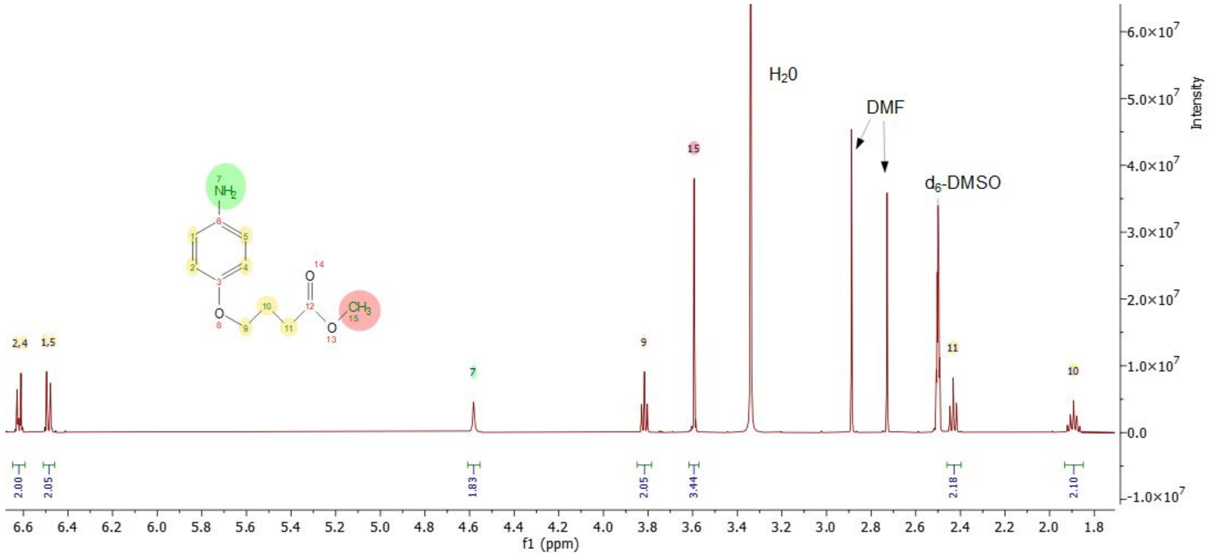

Figure S 38: ^{1}H NMR spectrum of methyl-4-(4-amino-phenoxy) butanoate **2c** (400MHz, DMSO-d_6). Complete functionalization of –OH of starting material determined by ^{1}H-NMR and the absence of any residual peaks of the starting material after reaction.

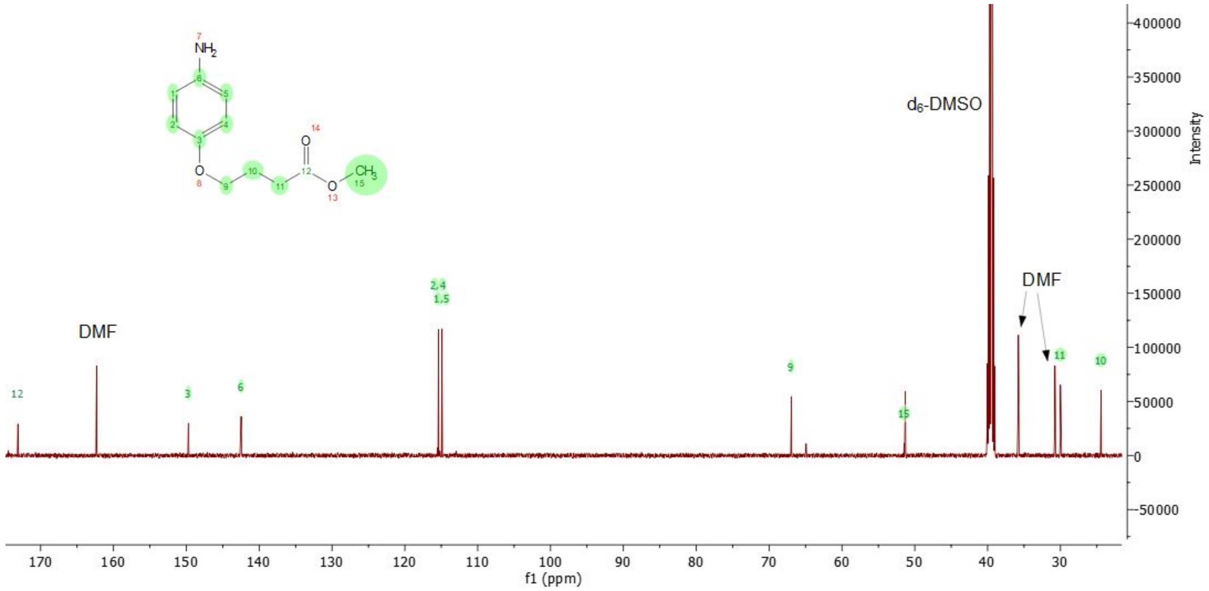

Figure S 39: ^{13}C NMR spectrum of methyl-4-(4-amino-phenoxy) butanoate **2c** (400MHz, DMSO-d_6).

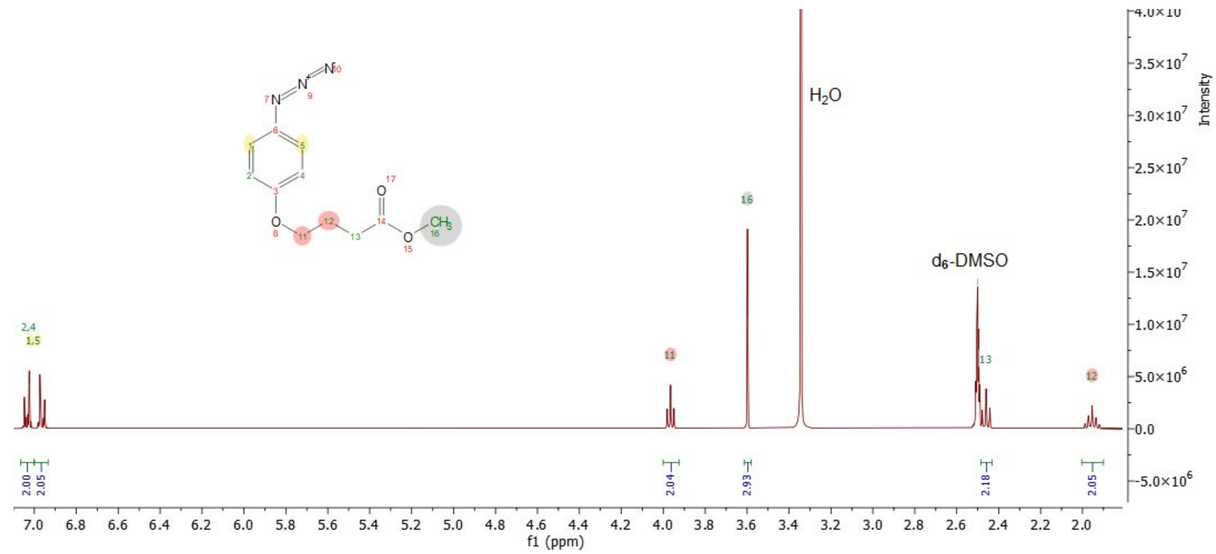

Figure S 40: ^{1}H NMR spectrum of methyl-4-(4-azido-phenoxy) butanoate **3c** (400MHz, DMSO-d_6). After column purification.

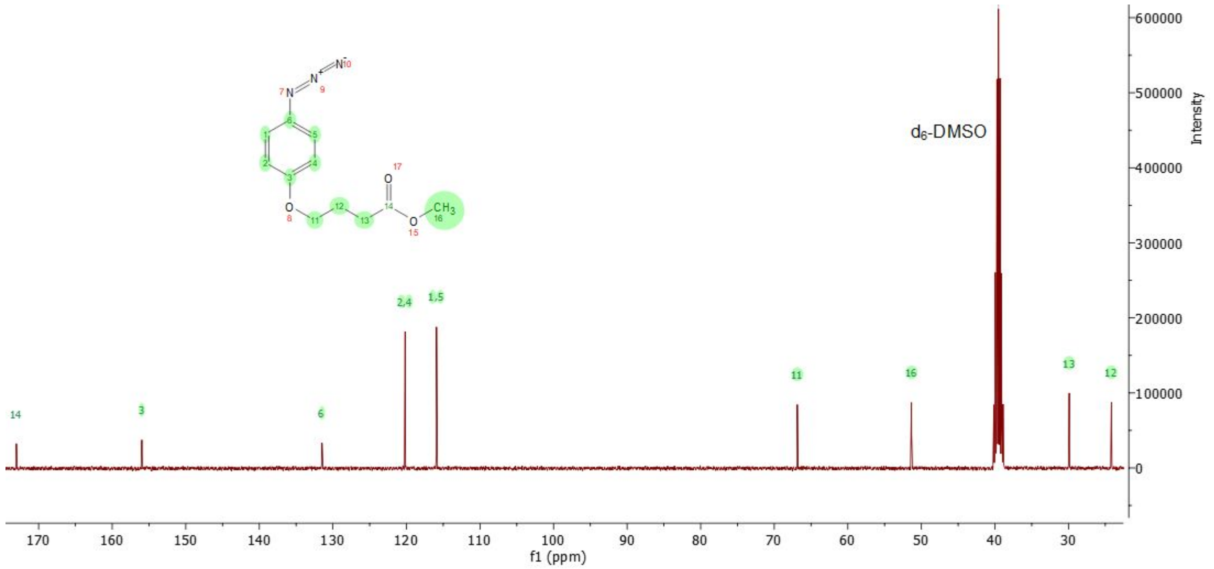

Figure S 41: ^{13}C NMR spectrum of methyl-4-(4-azido-phenoxy) butanoate **3c** (400MHz, DMSO-d_6). After column purification.

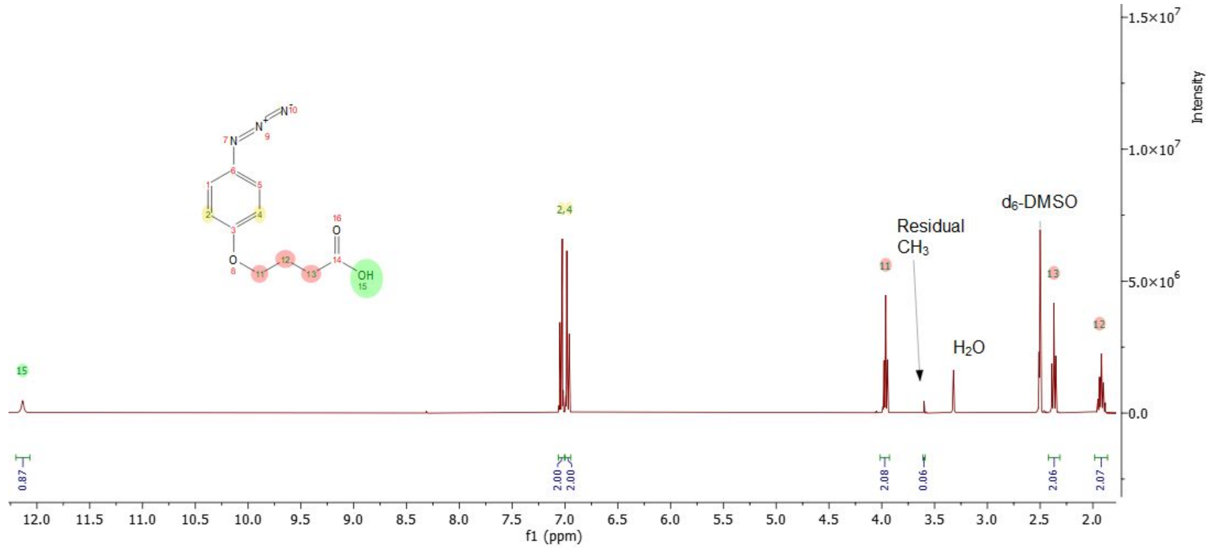

Figure S 42: ^{1}H NMR spectrum of (4-azido-phenoxy) butanoic acid **4c** (400MHz, DMSO-d_6). Ester hydrolysis ~97%, calculated by ^{1}H NMR and the residual –CH_3 peak.

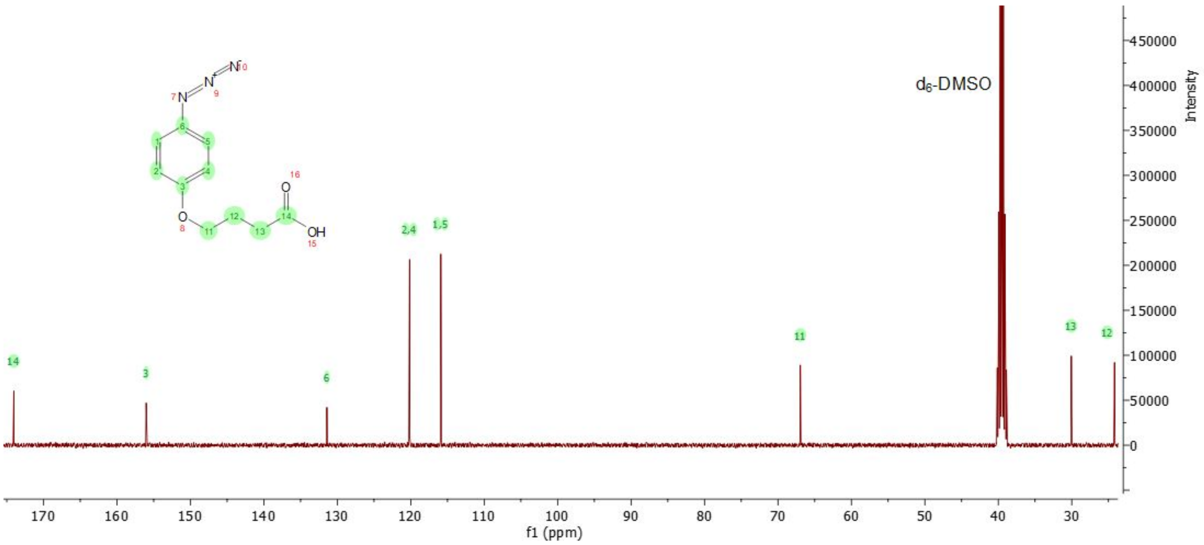

Figure S 43: ^{13}C NMR spectrum of (4-azido-phenoxy) butanoic acid **4c** (400MHz, DMSO-d_6).

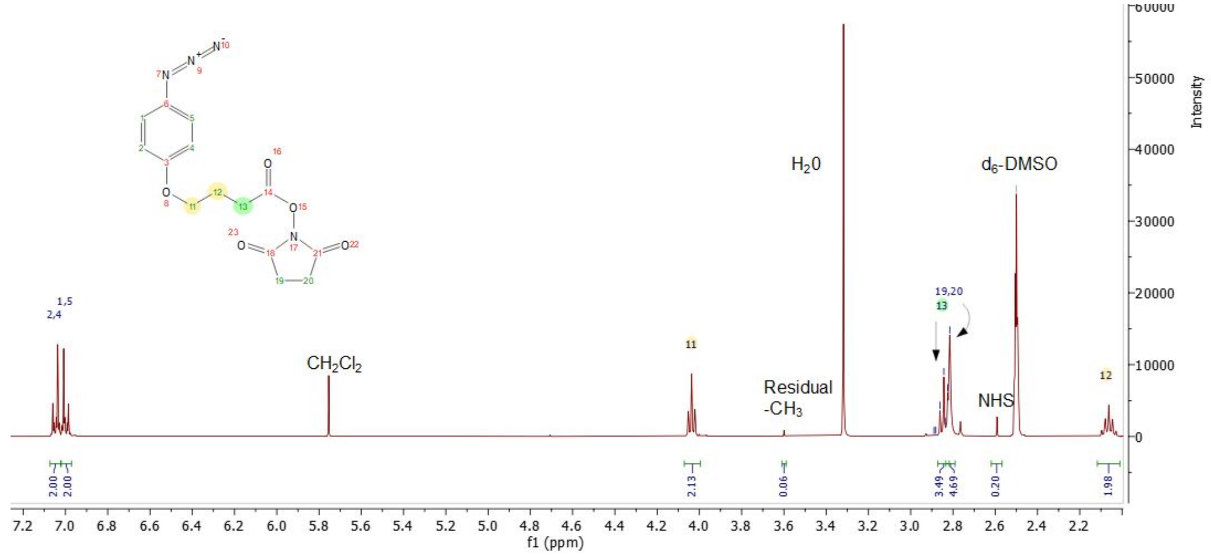

Figure S 44: ^{1}H NMR spectrum of (4-azido-phenoxy) butanoic acid – N-hydroxysuccinimide ester **5c** (400MHz, DMSO-d_6). Complete NHS ester formation and 95.4% purity calculated by ^{1}H NMR.

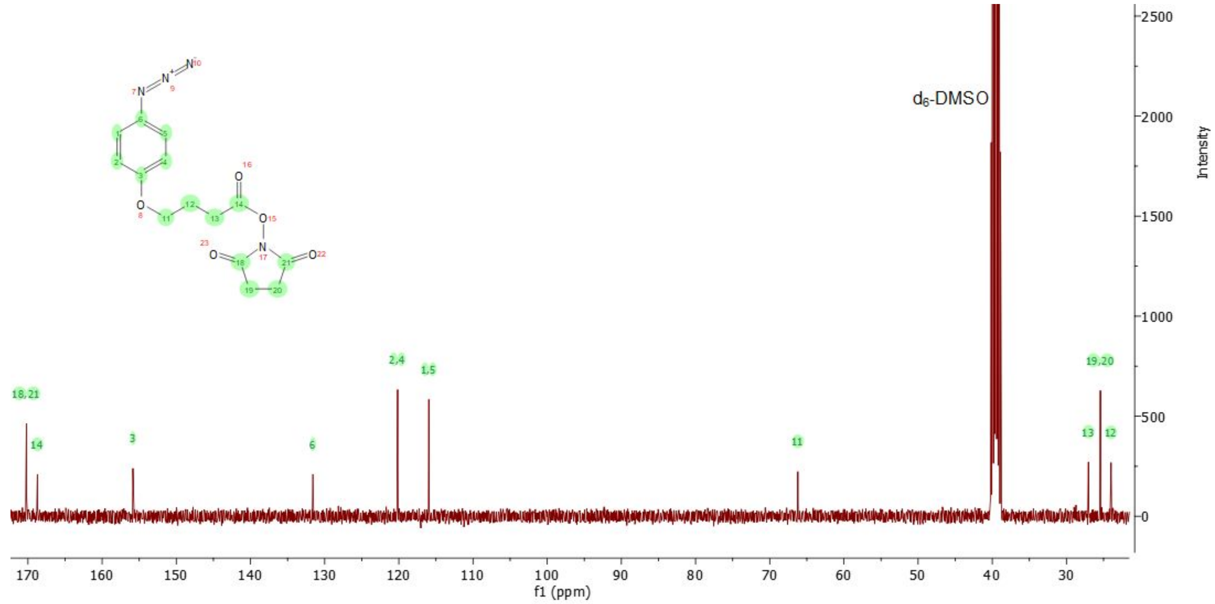

Figure S 45: ^{13}C NMR spectrum of (4-azido-phenoxy) butanoic acid – N-hydroxysuccinimide ester **5c** (400MHz, DMSO-d_6).

FTIR-ATR SPECTRA

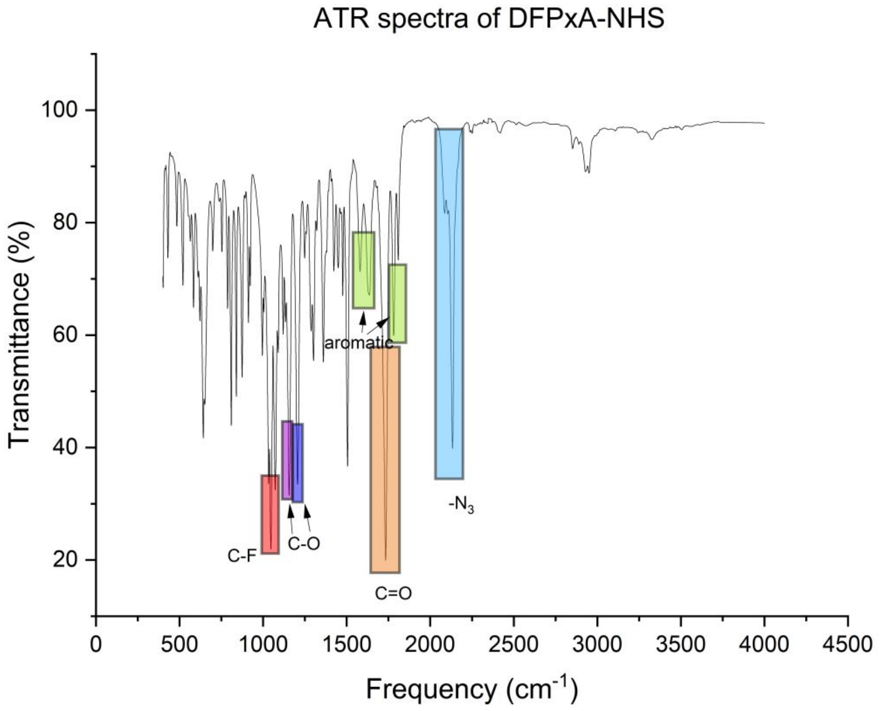

Figure S 46: FTIR-ATR spectrum of (4-azido-3,5-difluorophenoxy) butanoic acid – N-hydroxysuccinimide ester [DFPxA-NHS] **5a**. At 2135 cm^{-1} the strong asymmetric N_3 stretching vibration peak is clearly visible.

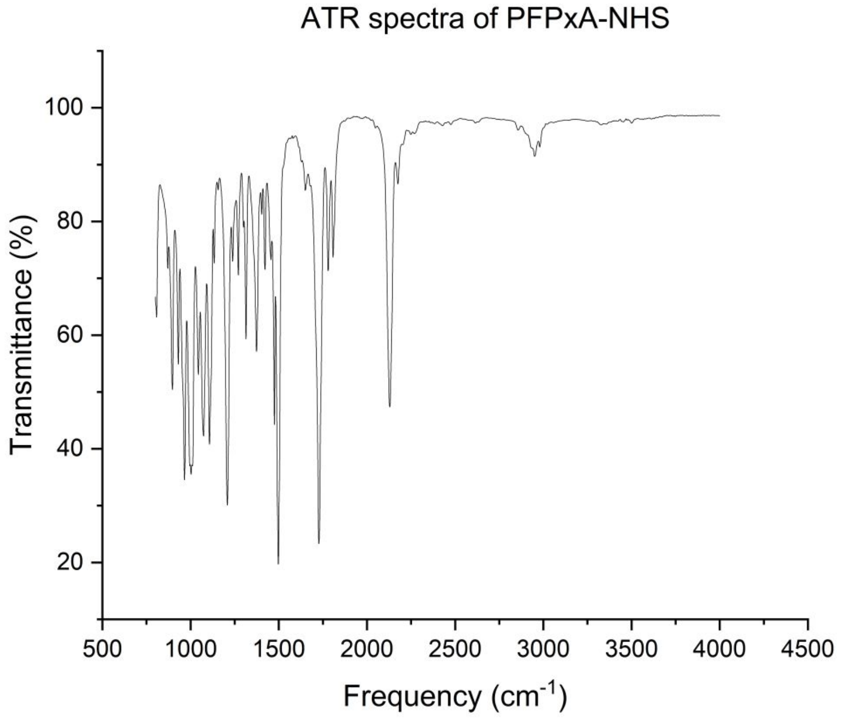

Figure S 47: FTIR-ATR spectrum of (4-azido-2,3,5,6-tetrafluorophenoxy) butanoic acid – N-hydroxysuccinimide ester [PFPxA-NHS] **5b**. At 2129 cm^{-1} the strong asymmetric N_3 stretching vibration peak is clearly visible.

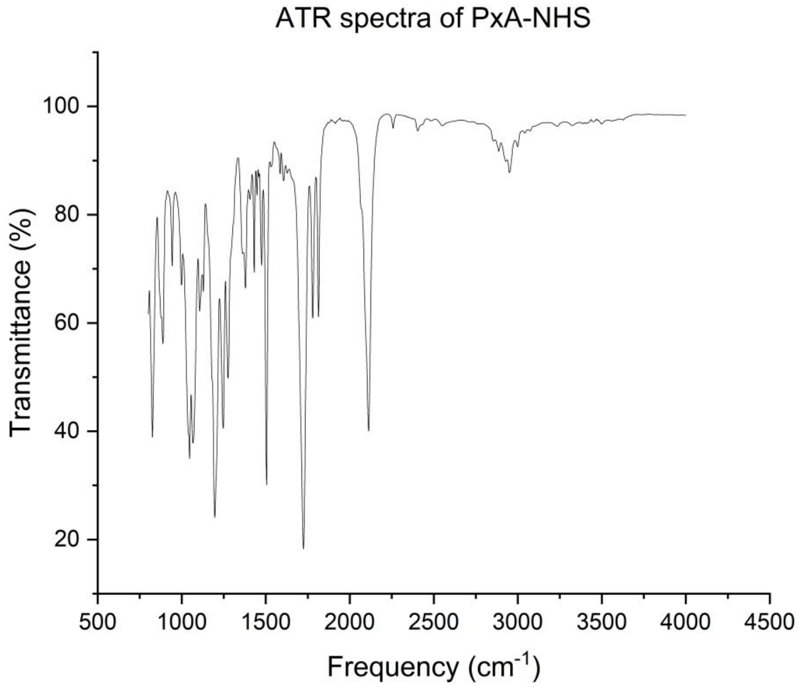

Figure S 48: FTIR-ATR spectrum of (4-azido-phenoxy) butanoic acid – N-hydroxysuccinimide ester [PxA-NHS] **5c**. At 2114 cm^{-1} the strong asymmetric N_3 stretching vibration peak is clearly visible.

REFERENCES

(1) Gritsan, N. P.; Gudmundsdóttir, A. D.; Tigelaar, D.; Zhu, Z.; Karney, W. L.; Hadad, C. M.; Platz, M. S. A Laser Flash Photolysis and Quantum Chemical Study of the Fluorinated Derivatives of Singlet Phenylnitrene. *J Am Chem Soc* **2001**, *123* (9), 1951–1962. https://doi.org/10.1021/ja9944305.

(2) Staudinger, H.; Meyer, J.; Chim Acta, H. The Chemistry of Azido Group. *Tetrahedron Lett* **1971**, *338*, 437.

(3) Poe, R.; Schnapp, K.; Young, M. J. T.; Grayzar, J.; Platz, M. S. Chemistry and Kinetics of Singlet (Pentafluorophenyl)Nitrene. *J Am Chem Soc* **1992**, *114* (13), 5054–5067. https://doi.org/10.1021/ja00039a016.

(4) L'abbé, G. Decomposition and Addition Reactions of Organic Azides. *Chem Rev* **1969**, *69* (3), 345–363. https://doi.org/10.1021/cr60259a004.

(5) Cardillo, P.; Gigante, L.; Lunghi, A.; Zanirato, P. Revisiting the Thermal Decomposition of Five Ortho-Substituted Phenyl Azides by Calorimetric Techniques. *J Therm Anal Calorim* **2010**, *100* (1), 191–198. https://doi.org/10.1007/s10973-009-0572-8.
